# Faster and lower-dose X-ray reflectivity measurements enabled by physics-informed modeling and artificial intelligence co-refinement

**DOI:** 10.1107/S2053273322008051

**Published:** 2022-10-01

**Authors:** David Mareček, Julian Oberreiter, Andrew Nelson, Stefan Kowarik

**Affiliations:** a Physikalische und Theoretische Chemie, Universität Graz, Heinrichstraße 28, Graz, 8010, Austria; b ANSTO, Locked Bag 2001, Kirrawee DC, NSW 2232, Australia; DESY, Hamburg, Germany

**Keywords:** neutron reflectivity, X-ray reflectivity, neural networks, co-refinement, *in situ* measurement

## Abstract

An approach is presented for analysis of real-time X-ray reflectivity (XRR) process data not just as a function of the magnitude of the reciprocal-space vector *q*, as is commonly done, but as a function of both *q* and time. The real-space structures extracted from the XRR curves are restricted to be solutions of a physics-informed growth model and use state-of-the-art convolutional neural networks (CNNs) and differential evolution fitting to co-refine multiple time-dependent XRR curves *R*(*q*, *t*) of a thin film growth experiment. Thereby it becomes possible to correctly analyze XRR data with a fidelity corresponding to standard fits of individual XRR curves, even if they are sparsely sampled, with a sevenfold reduction of XRR data points, or if the data are noisy due to a 200-fold reduction in counting times. The approach of using a CNN analysis and of including prior information through a kinetic model is not limited to growth studies but can be easily extended to other kinetic X-ray or neutron reflectivity data to enable faster measurements with less beam damage.

## Abstract 2

An analysis approach for co-refinement of X-ray reflectivity -p0measurement is presented, which works with sparsely sampled or noisy data for seven- to 200-fold speed increases.

## 1. Introduction

X-ray reflectivity (XRR) and neutron reflectivity (NR) are well known techniques for studying ultra-thin layers (Tolan, 1999 ▸; Holý *et al.*, 1999 ▸; Braslau *et al.*, 1988 ▸; Skoda *et al.*, 2017 ▸; Russell, 1990 ▸; Kowarik *et al.*, 2006 ▸; Pietsch *et al.*, 2004 ▸). Due to the penetrating nature of X-rays and neutrons, the measurements can be taken in various environments, such as in a vacuum, or in liquids or gases. This has led to numerous applications in many fields that profit from *in situ* and real-time measurements, such as the solid–electrolyte interface growth during the process of li­thia­tion in lithium-ion batteries (Liu *et al.*, 2016 ▸; Cao *et al.*, 2016 ▸) and studies of layer-by-layer growth (Kowarik *et al.*, 2006 ▸; Hanke *et al.*, 2012 ▸; Joress *et al.*, 2018 ▸; Krause *et al.*, 2019 ▸; Bommel *et al.*, 2014 ▸). Real-time measurements usually consist of repeated reflectivity scans at defined time intervals, and each measurement results in a single reflectivity curve that represents the growth process at a certain point in time. From these scans, structural film properties including thickness, roughness and scattering length density (SLD) can be extracted as a function of time *t*, although the analysis is non-trivial and fitting the reflectivity 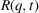 only as a function of *q* wastes information available from data at adjacent time steps. An important challenge for XRR and NR curves is the reconstruction of real-space sample structures from reciprocal-space scattering data and the reliability of this procedure. Using the Parratt formalism (Parratt, 1954 ▸) to calculate reflectivity curves allows one to easily obtain the shape of the reflectivity curve for a given thin layer structure. However, the inverse reconstruction of sample parameters from an XRR curve is highly non-trivial (López García & Rivero, 2021 ▸) and multiple non-unique solutions are possible. Results are usually obtained by iterative least mean squares (LMS) fitting algorithms or advanced genetic algorithms, which are available in various XRR and NR programs (Nelson, 2006 ▸; Nelson & Prescott, 2019 ▸; Björck & Andersson, 2007 ▸; Danauskas *et al.*, 2008 ▸; Lazzari, 2002 ▸).

In contrast to the traditional, manual fitting processes, artificial intelligence techniques in the form of artificial neural networks (NNs) (Bishop, 1994 ▸) have become available recently for fast, accurate fits of XRR or NR curves without human input (Greco *et al.*, 2019 ▸, 2021 ▸; Liu *et al.*, 2019 ▸; Park *et al.*, 2017 ▸; Vecsei *et al.*, 2019 ▸; Chen *et al.*, 2021 ▸; Kim & Lee, 2021 ▸; Mironov *et al.*, 2021 ▸; Doucet *et al.*, 2021 ▸; Andrejevic *et al.*, 2022 ▸; Loaiza & Raza, 2021 ▸). The convolutional neural network (CNN) approach for inverting XRR data has become popular due to better computer performance resulting from relatively cheap modern graphics and central processing units (GPUs and CPUs), as well as the accessibility in recent years of free CNN programming frameworks such as *TensorFlow* (https://www.tensorflow.org/) and *PyTorch* (https://pytorch.org/). A drawback of the CNN approach is that it needs a large volume of training data for CNN training; however, in the case of XRR and NR, these training data can be easily simulated. A CNN trained on synthetic data can then be applied to experimental data including time series of XRR curves. Owing to the comparatively large number of data points, time-series measurements can profit from automated CNN analysis and co-refinement.

In real-time growth, most of the parameters of a thin film structure change continuously and are dependent on each other; for example, many individual thickness parameters for each curve can be replaced by a growth rate. With a corresponding growth model, we can re-parametrize (Campbell *et al.*, 2018 ▸) the structural models of the thin film at each time step with very few parameters rather than using individual parameter sets for each time step. Moreover, the XRR fits of individual time steps are lacking the improvement of parameter estimates that co-refinement (McCluskey *et al.*, 2019 ▸) with previous or following curves can bring, and therefore the prediction is noisier than one obtained by using an underlying growth model. In this paper we report an implementation of a growth model, whose parameters are determined with the *refnx* software (Nelson & Prescott, 2019 ▸) in a growth model co-refinement of the experimental growth data. Secondly, we trained a CNN with synthetic data generated via the growth model and then used the CNN to predict the structural evolution from the experimental growth data. We show that we can significantly speed up the measurement and reduce the X-ray exposure of the sample through growth model co-refinement and CNN approaches, because they allow one to only use sparse sampling and shorten the exposure times. This achievement is enabled through (i) the incorporation of prior knowledge in the form of a growth model, (ii) re-parametrization of the fit problem, and (iii) co-refinement or CNN image recognition of multiple XRR curves at once.

## 2. Methods

### 2.1. Growth model

Using a (rate-equation) model for kinetic processes is crucial for our goal of co-refinement of real-time XRR data sets, and for our example of crystalline thin film deposition a growth model is needed. Crystal growth of highly perfect crystalline layers ideally proceeds in a layer-by-layer fashion, but also deviations from a layer-by-layer type of growth and roughening are common. Numerous models have been developed over recent decades which can describe this layer-by-layer growth of thin films (Cohen *et al.*, 1989 ▸; Braun *et al.*, 2003 ▸; Trofimov *et al.*, 1997 ▸). These models are quite general and can all fit a variety of growth scenarios from layer-by-layer growth to layer-plus-island growth or rough 3D growth. Here we employ a model used by Woll *et al.* (2011 ▸) for organic molecular thin films, even though we note that the above-mentioned models could also be used. This model generates the surface roughness and thickness evolutions with realistic behavior, such as oscillating roughness for layer-by-layer growth. The growth rate in the model is described via the parameter *G_n_* for the *n*th layer. A parameter θ_*n*,cr_ defines the critical layer coverage of the *n*th layer, where the *n*+1-th layer starts to nucleate on the top of the *n*th layer. Once nucleated, the feeding zone ξ_*n*_ then is the size of the zone on top of the *n*th layer, where molecules contribute to the growth of the *n*+1-th layer. Molecules outside the feeding zone will diffuse over the edge into the *n*th layer.

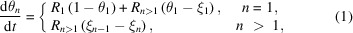

where *R_n_* is the growth rate of the *n*th layer and the feeding zone size is given as

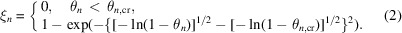




On the basis of this model, the thickness and roughness of the growing thin film can be calculated at each moment in time, and together with the SLD, used to generate the corresponding XRR curves. The SLD parameter is set as a fixed value in our model even though different structural phases in the first monolayers slightly modify the SLD (Kowarik *et al.*, 2006 ▸; Frank *et al.*, 2014 ▸). Here, we intentionally use a simple model of a fixed SLD for thin films and describe partially filled, growing layers via an oscillating film roughness during initial layer-by-layer growth. With the layer-by-layer growth model, the evolution of the thin film can be defined with ten parameters. Four growth rate parameters *G_n_* were used to account for different sticking coefficients on the substrate and the first layers, where *n* = 1, 2, 3 stands for the growth rate of the first, second and third layers, while *G*_4_ is used as the growth rate for all the following layers. The next parameter to be defined is θ_cr_ for each layer. To reduce the parameter space we calculated θ_*n*,cr_ from the following equation:

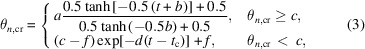

where *a* and *b* parametrize the time evolution of θ_*n*_,_cr_ with growth time *t*. When the θ_*n*_,_cr_ value reaches a threshold, represented by a crossover parameter *c*, the function for θ_*n*_,_cr_ is switched to an exponentially decaying function. Here, the parameter *d* is the steepness of exponential decay, *f* is the asymptotic value for large *t*, and *t*_c_ is the time when the θ_*n*_,_cr_ value reaches the crossover value. All ten parameters were randomly but uniformly distributed to generate a synthetic data set for NN training and used as training labels. Note that the above growth model and parametrization of θ_cr_ restrict the possible growth scenarios we can fit and might lead to wrong fit results if the real scenario is not covered. However, the parameter ranges are chosen to be wide enough to include 3D growth and roughening from the very first layer, to layer-by layer growth followed by roughening as well as layer-by-layer growth with continuing roughness oscillations over the whole thickness range. The ten parameters are collectively denoted as growth model parameters in the following.

### 2.2. XRR curve simulation

We simulated XRR layer-by-layer growth using an adaptation of the optical matrix method (Abelès, 1950 ▸; Heavens, 1960 ▸), which is computationally more efficient than the recursive Parratt formalism (Parratt, 1954 ▸). For this purpose, parts of the *Refl1D* (Kienzle *et al.*, 2022 ▸) source code were used. We assumed that our thin film sample consists of three thin film layers: two for the substrate (silicon and native oxide) plus the deposited thin film. The properties of Si/SiO_2_ substrates are known from our prior work and thus we used a constant roughness for the SiO_2_ and Si layers of 1 and 2.5 Å, respectively, and a native oxide thickness of 10 Å. Furthermore, the SLDs of those layers were assumed to be constant with values of 17.8 × 10^−6^ and 20 × 10^−6^ Å^−2^, respectively (Greco *et al.*, 2019 ▸). The XRR curves were simulated in a *q* range between 0.01 and 0.14 Å^−1^ at 109 equally spaced points.

For the following, we appended all individual XRR curves corresponding to a given growth scenario into a single XRR time-series matrix *R*(*q*, *t*). Due to the shape of the experimental di­indeno­perylene (DIP) data, *R*(*q*, *t*) contains 109 *q* points and 80 time steps; we also created a synthetic data set with the same dimensions. Each one of the curves corresponds to a certain thickness, roughness and SLD of the film layer during the growth. The fixed SLD and the evolution of the thickness d(*t*) and roughness σ(*t*) are later on in the text collectively referred to as a thin film growth scenario. The growth of the simulated thin film started from a thickness of 0 Å and the final thickness was varied from 180 to 450 Å using the growth rate parameters (*G*_1_, *G*_2_, *G*_3_, *G*_4_). The final roughness of the film was allowed to vary from 8 to 40 Å. The SLD was set in a range from 7 × 10^−6^ to 18.5 × 10^−6^ Å^−2^. We simulated 25 000 *R*(*q*, *t*) data sets to get a sufficient quantity of data for the CNN training. The simulated *R*(*q*, *t*) data were split into training and validation data sets, where the validation data set contained 10% of the generated data, *i.e.* 2500 simulated data sets *R*(*q*, *t*). The data generation took 90 min on 20 independent CPU threads; this procedure can be faster if *refnx* is used. We took the natural logarithm of all generated data sets and then normalized them to unity before using them as an input for the CNN. This method of normalization is widely used, including in previous work by us; however, we note that there are alternatives for normalizing reflectivity data (Loaiza & Raza, 2021 ▸; Doucet *et al.*, 2021 ▸; Kim *et al.*, 2021 ▸). In this way, we reduced the value spread of the input data set, because the CNN training is faster for input and output data in the range of 0 to 1. Therefore, all growth model parameters which are the output of the CNN were also normalized to unity.

### 2.3. Neural network

**Figure 1:**
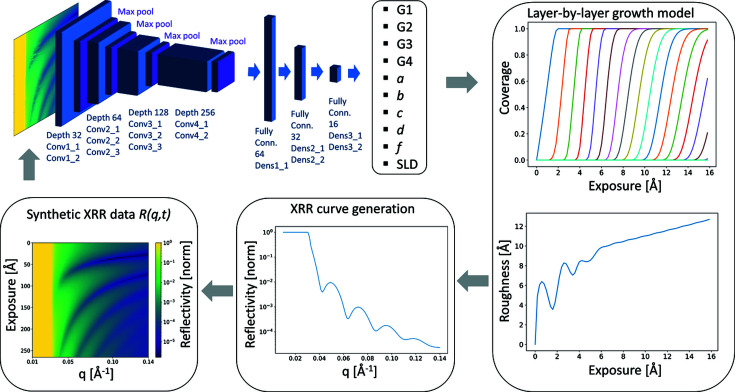
From a time-dependent XRR data set 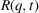 a CNN predicts ten parameters of a time-dependent layer-by-layer growth model. From these parameters, the thin film growth scenario with thickness and roughness evolution is calculated. For different growth scenarios, we create simulated XRR and synthetic 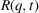 data sets that can be used to train the CNN before applying it to real data.

We utilized 2D CNNs, commonly used for image recognition, to predict growth model parameters from the 2D data set *R*(*q*, *t*). We employed a smaller version of the standard VGG16 CNN which is a common architecture used for image recognition (Simonyan & Zisserman, 2015 ▸). In this architecture, 2D data are processed from a set of input neurons to a set of output neurons through hidden layers of neurons. The input layer of the CNN takes the *R*(*q*, *t*) data. The architecture of our CNN is graphically depicted in Fig. 1 ▸. As an activation function, a simple ReLU (rectified linear function) unit was chosen for all layers. The optimization algorithm employed in this work is adaptive moment estimation (ADAM) (Kingma & Ba, 2015 ▸). Our code was written in Python 3.7 with the use of the *TensorFlow* software library. The CNN was trained for 3000 epochs (4 h) on a GPU (Nvidia GeForce RTX 2080 Ti) and Intel Core i5-9600K CPU.

### 2.4. Experimental data

As an experimental example, we used the previously published (Kowarik *et al.*, 2006 ▸) real-time XRR data set related to growth of the organic semiconductor molecule DIP via organic molecular beam deposition on an Si/SiO_2_ wafer at 403 K substrate temperature. The experiments were per­formed at beamline ID10B at the ESRF in Grenoble, France, in a small ultra-high vacuum chamber, equipped with a Be window, effusion cells and thickness monitor, at X-ray wavelengths of 0.903 Å. The original experimental data set was measured with 52 *q* points per curve and interpolated to 109 points per curve in the same *q* range. For our CNN approach, the experimentally obtained *R*(*q*, *t*) was used as CNN input, from which the CNN predicted growth model parameters and the corresponding thin film growth scenario. For the CNN performance evaluation on the experimental data, the thin film growth scenario determined by the model was compared with a manual LMS fit using the *Parratt32* software (Goedel *et al.*, 1999 ▸). The output of *Parratt32* is the thickness, roughness and SLD for every curve, which is more than 240 parameters. The only varying parameters were parameters corresponding to the thin DIP layer, and the *q* range was the same as for the CNN, from 0.01 to 0.14 Å^−1^. *Refnx* (Nelson & Prescott, 2019 ▸) was used to co-refine all XRR curves, with the growth model being incorporated into the analysis, reducing the number of fit parameters to 10. In this last approach the varying growth model parameters are used to calculate the thickness/roughness of the system at each of the time steps.

## 3. Results

**Figure 2:**
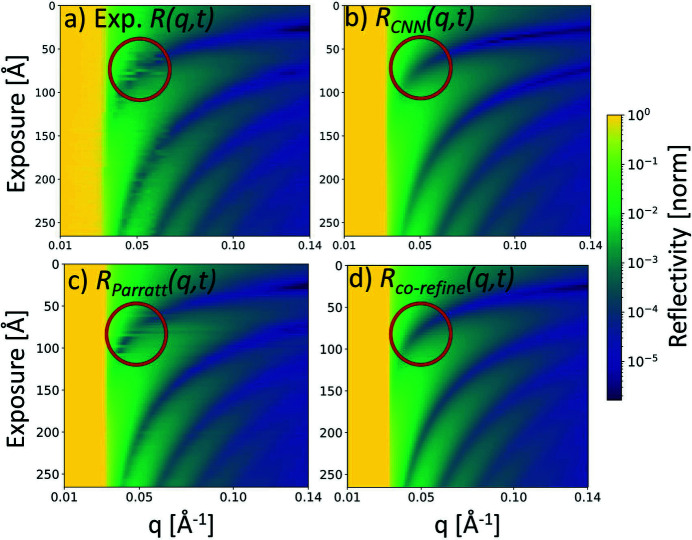
The experimental 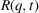 in (*a*) is compared with different fitted XRR curves. (*b*) shows the CNN prediction with a χ^2^ deviation value of 5.0; a manual *Parratt32* fit (*c*) has a lower deviation of 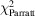 = 1.7 as it also fits experimental noise (red circle). The growth model co-refinement (*d*) yields 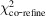 = 9.2.

We used our CNN to predict the growth model parameters from the experimental *R*(*q*, *t*) shown in Fig. 2 ▸(*a*). Then these parameters were used to generate a new fitted *R*_CNN_(*q*, *t*) [Fig. 2 ▸(*b*)], and we compared the two *R*(*q*, *t*)’s by calculating a χ^2^ value as a measure of goodness of fit, with χ^2^ defined as

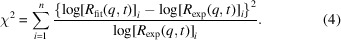




From the comparison in Figs. 2 ▸(*a*) and 2 ▸(*b*), it is obvious that the Kiessig fringes in the experiment and in *R*_CNN_(*q*, *t*) are at the same position. Moreover, the damping of oscillations increases at higher *q* and *t* due to increased roughness in both 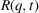 ’s. The reflectivity range and minimum reflectivity values agree very well in the two images. The χ^2^ deviation between the experiment and *R*_CNN_(*q*, *t*) was calculated as 5.0. For the *Parratt32* fit where we fit curves individually (individual fit), we found 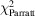 = 1.7. Finally, with an automatic growth model co-refinement using *refnx* we obtained 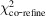 = 9.2. The fitted XRR curves *R*_Parratt_(*q*, *t*) and *R*_co-refine_(*q*, *t*) are shown in Figs. 2 ▸(*c*) and 2 ▸(*d*) and also are very similar to the experimental data. All the χ^2^ errors were calculated by the same method, without use of *Parratt32* or *refnx* implemented error calculations. The individual *Parratt32* curve fit adapts to spurious experimental noise features (red circle) which partly explains the lower χ^2^ value of the individual curve *Parratt32* fit compared with the CNN fit. The results are plotted as a function of *q* and exposure. The exposure is *t* *j*, where *t* is the time and *j* is the molecular flux. In an experimental setup, the molecular flux does not need to be always constant and it is straightforward to implement these changes when the exposure is used as the axis. Note that the *Parratt32* manual fit is a slow process (hours) where the user selects bounds and starting points in the fit of thickness, roughness and SLD of the DIP layer for every curve. The growth model co-refinement approach is significantly faster (30 min) than the *Parratt32* manual fit but still much slower than a CNN prediction, which takes up to 55 ms, when not counting the 5.5 h of CNN data generation and training time.

**Figure 3:**
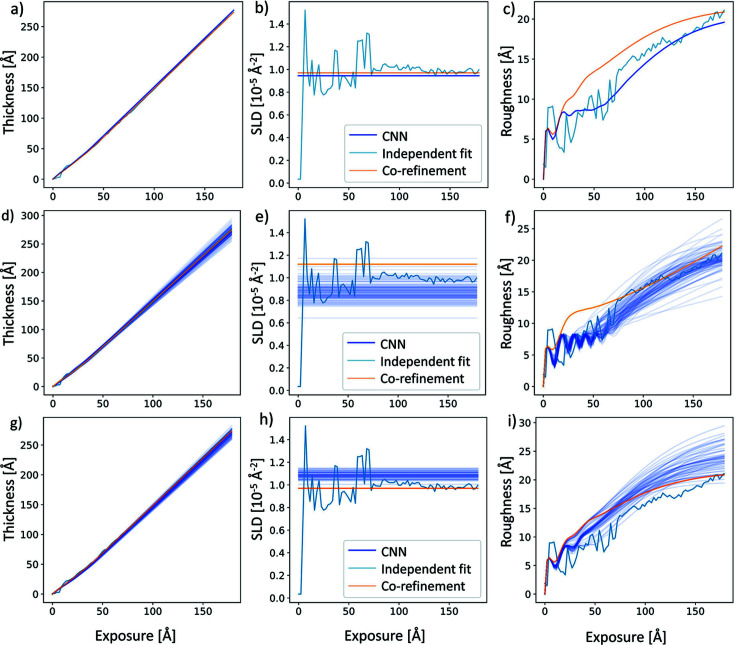
(*a*) Thickness, (*b*) SLD and (*c*) roughness predictions derived from the experimental XRR data 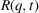 in Fig. 2 ▸ agree reasonably well between standard individual curve fits, growth model based CNN and co-refinement results. (*d*)–(*f*) For a reduced data set of only 200 reflectivity points in 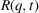 , the CNN and the growth model co-refinement fit still fit the data set correctly. A set of CNN prediction curves are shown for different, random selections of 200 points out of the full data set, giving an indication of the CNN prediction variability. (*g*)–(*i*) For noisy data, the growth model co-refinement needs a count rate of only ∼1000 counts in the total reflection region to allow for adequate reconstruction, while the CNN can fit data even with ∼300 counts in the total reflection region. Again, CNN predictions of a set of curves with different simulated photon shot noise are shown to indicate the variability of CNN predictions.

Besides the comparison of raw experimental data and the various fits of 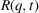 , we also evaluated the thin film growth scenarios obtained with the CNN, independent 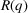 fits and the growth model co-refinement [Figs. 3 ▸(*a*)–3 ▸(*c*)]. In the plots of the thickness evolution during growth [Fig. 3 ▸(*a*)] and the SLD [Fig. 3 ▸(*b*)] the CNN prediction, independent fit and growth model co-refinement closely align. The roughness comparison in Fig. 3 ▸(*c*) shows that all fits are roughly comparable with some deviations for the growth model co-refinement method. The oscillations of the roughness visible in the CNN fit at the beginning of the growth are due to the layer-by-layer growth as filled layers are smoother and half-filled layers during layer-by-layer growth are rougher. Overall, the growth model based on the CNN predictions and the growth model co-refinement can explain the real-time XRR curves with far fewer parameters and produce results that come close to manual fits in terms of the χ^2^ metric of the fit quality and match well the results of the thin film growth scenarios.

### 3.1. Reducing the number of data points in a measurement – sparse sampling

In the following, we demonstrate the high performance of the CNN and the growth model co-refinement approach on a sparsely sampled data set with a very low quantity of data. The 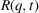 of DIP has been reduced using a dropout layer from *Keras* (Ketkar, 2017 ▸) to remove a certain percentage of the experimental data points by setting them to zero. These points can therefore be skipped in a faster measurement. For the CNN to work with reduced data sets, it is necessary to further train the CNN so that it learns to ignore unavailable data points. We retrained the previously optimized CNN with a new data set of 120 000 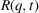 ’s which had a varying degree of dropout reduction from 0 to 99.9%. The CNN was retrained for 30 epochs without any sign of overfitting. The experimental 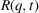 with a reduced number of data points was then used as an input for the retrained CNN.

To demonstrate the accuracy of predictions with reduced data sets, we compare the CNN prediction and growth model co-refinement from only 200 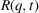 data points and the baseline individual curve fit from the full data set in Figs. 3 ▸(*d*)–3 ▸(*f*). We find that the growth model predictions/fits are close to the benchmark of individual curve fits of the full data set. Note that the CNN predictions are shown as a set of curves, each of which corresponds to a different set of randomly selected sets of 200 data points. This illustrates the fairly narrow spread of the predicted results and demonstrates that the result does not strongly depend on a specific set of 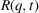 points. The growth model co-refinement is also able to fit reduced 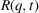 XRR data sets down to 200 reflectivity points and is very similar to CNN performance.

**Figure 4:**
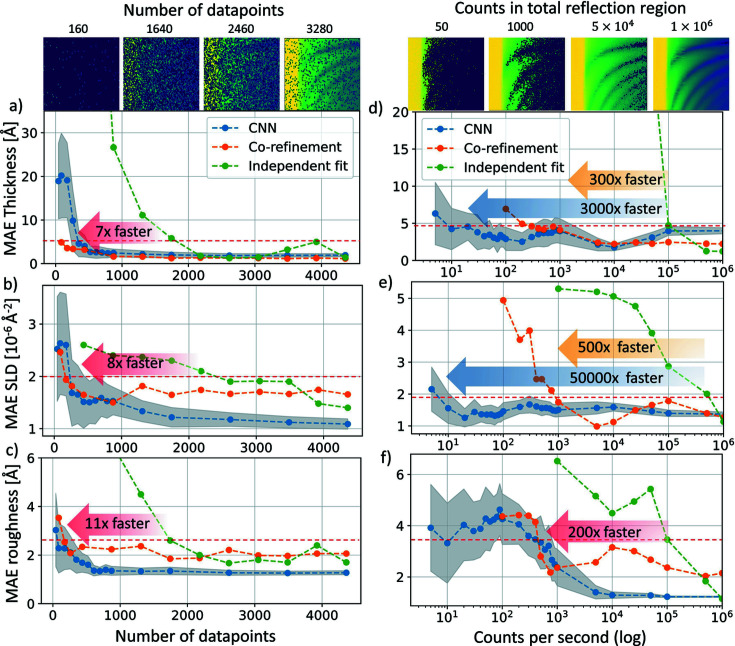
(*a*)–(*c*) The mean absolute error of the thickness, roughness and SLD increases when fewer data points are available for fitting. The CNN and the growth model co-refinement can analyze sparsely sampled data with a 6–11× lower number of measured reflectivity values compared with the individual fit. Panels (*d*)–(*f*) illustrate the effect of simulating increasing amounts of photon shot noise for the 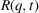 data, which would occur for shorter integration times or weaker X-ray sources. The growth model co-refinement is accurate for noise levels more than two orders of magnitude higher than for the independent fit of individual XRR curves. The CNN can predict correct parameters with even higher noise levels than the co-refinement approach.

In Figs. 4 ▸(*a*)–4 ▸(*c*) we show how the fit results degrade when systematically reducing the number of XRR data points entering the fit. This allows us to quantify the gains in speed and/or reduction in X-ray dose that are possible by reducing the XRR data-set size. We compare independent fits of all the individual curves using the batch fit mode of *refnx*, a growth model co-refinement of all curves at once and the CNN analysis of all curves. As three figures of merit, we choose the mean absolute error (MAE) for thickness *d*(*t*), density SLD(*t*) and roughness σ(*t*) in Figs. 4 ▸(*a*)–4 ▸(*c*) calculated for each of the above three analysis strategies in relation to our manual fit of the whole data set. A reasonable threshold for an acceptable MAE was set to the value indicated by the horizontal lines in Fig. 4 ▸, which corresponds to a doubling of the lowest MAE value for the full data set. We find that the growth model co-refinement and CNN predictions provide almost identical results as the error stays below our limit down to 200 data points, meaning that each XRR curve contains only two or three data points. The independent fit of each XRR curve by itself starts to fail earlier, at 1700–2200 points in the data set. This is understandable, because two to three data points do not constitute an XRR curve that can be fitted, and between 20 and 30 points are needed for fitting each of the 80 time steps of the growth. Also note that, while the growth model co-refinement and the CNN both perform well for sparsely sampled data sets, the batch fit takes 30 min to fit the data set and CNN can make a prediction in ∼55 ms. This faster prediction time from a CNN enables us to randomly select 100 different sets for each point in the curve and run the CNN prediction 100 times, so that we can give a confidence interval for the MAE in the CNN analysis.

### 3.2. Reduced counting time or lower X-ray flux

As a second strategy to speed up the measurement and reduce the X-ray dose, we also investigated the effects of reduced count rate per data point instead of reducing the number of data points. Through a shorter integration time, or alternatively lower incident X-ray flux, the photon shot noise increases. This can be modeled by replacing the count rate 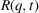 at a given *q* point with a random value of counted photons *x* selected according to a Poissonian probability distribution:

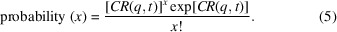

Here, *C* is the maximum number of counts in the total reflection region. Since the normalized XRR intensities 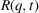 only range from 0 to 1, they must be scaled by the count rate *C* before calculating the photon shot noise. This assumes constant integration times and disregards the footprint correction needed for small sample sizes, but both these effects can be easily accounted for if needed in a particular experiment. A reflectivity value 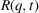 is then replaced by 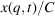 . We introduced the Poisson-distributed noise into our training data and created a data set of 500 000 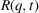 in the range of *C* from 5 to 1 × 10^8^. With the new noisy data set, we retrained our CNN for 20 epochs.

Next, we introduced the Poisson-distributed noise into the experimental data and generated 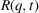 where the range of *C* was from 5 to 10^6^ counts in the total reflection region. We used the retrained CNN to predict the thin film growth scenario from the noisy experimental 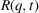 , finding good CNN prediction performance even for extremely low X-ray flux. Next, we fitted noisy data with the growth model co-refinement of all curves. In Figs. 3 ▸(*g*)–3 ▸(*i*) we compare CNN prediction from noisy data with *C* = 300 and the growth model co-refinement with *C* = 1000 counts at the total reflectivity edge. Note that the 100 CNN prediction curves show each result from a different realization of shot noise to illustrate the variance in CNN predictions introduced by shot noise. The results are compared with our independent fit of the experimental 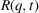 without added noise as reference. We get reasonable results for thickness, SLD and roughness, but note that the CNN prediction with only 300 counts overestimates the final roughness. This is not surprising, because the data at high *q*/low reflectivity will be significantly degraded because of the reduced count rate. For example, if the direct beam rate is 1000 Hz, and the background is 1 Hz, then one will not be able to go below *R* = 0.001.

In Figs. 4 ▸(*d*)–4 ▸(*f*) we quantify the performance of the CNN fit, growth model co-refinement and independent curve fits on data with different levels of noise. The point of reference is our manual fit of the full experimental data set without added noise. Again, the individual curve fit performs worst, and introduction of a physics-based growth model in either the co-refinement or the CNN fit significantly increases the robustness to noise and lowers the MAE. The growth model co-refinement can fit noisier data with a 200× to 500× reduction in counting time/X-ray flux over the individual curve fit of noisy data. The exact gain depends on the parameter, with roughness being the hardest to predict, offering only a 200× gain, and thickness being the easiest to predict and offering a 500× gain. Interestingly, the CNN outperforms both the individual curve fit and the growth model co-refinement, and can predict thickness from 3000× noisier data than the individual fit and SLD from up to 50 000× noisier data than the individual fit. In Table 1 ▸, we summarize the speed-up provided by the growth model implementation into the fitting algorithms. We used the same range in which the thin film parameters were allowed to vary for the growth model co-refinement and CNN. For the independent fit of each individual curve, we were not able to use exactly the same parameters but we used comparable thickness, roughness and SLD parameter ranges. Performance gains through using re-parametrization and co-refinement with the CNN are shown to be greater than sevenfold for data reduction and more than 200-fold for noisy data sets from shorter integration times or lower flux.

## 4. Discussion

The approach presented here enables a more than two orders of magnitude reduction in X-ray/neutron photon dose and faster experiments due to the implementation of a physics-based growth model and analysis of all real-time XRR data sets at once. It has already been shown that the prediction of CNN for a single noisy XRR curve is possible (Greco *et al.*, 2021 ▸) and an analysis of intentionally high-noise NR was published recently (Aoki *et al.*, 2021 ▸; Doucet *et al.*, 2021 ▸). All these studies show that an NN can perform well on noisy XRR or NR data. However, the re-parametrization and co-refinement of multiple XRR curves used here enable us to achieve correct prediction from an even lower quantity of data than reported before. This performance increase can be obtained either by the implementation of the growth model into a co-refinement approach or by using a CNN. The growth model co-refinement is better for single experiments because it does not need any training data or CNN training time. However, the analysis of the data set is significantly slower at ∼30 min compared with 55 ms with a CNN. The growth model co-refinement could be sped up by implementing a simpler growth model, but our example of a rate equation/differential equation based temporal model is not untypical for processes measured in real time. Moreover, we expect that some further speed-ups may be achievable by improving the ordinary differential equation solving step in Python. For the CNN case, implementing a growth model for training data generation and training of the CNN take 5.5 h, which is slower than a single growth model co-refinement.

The CNN analysis has advantages if a growth study encompasses repeated measurement under varying conditions and necessitates several fits. Also, the CNN predictions lie closer to results from a manual fit in Fig. 3 ▸ than growth model co-refinement. Due to its prediction speed, the CNN could be used as a process analytical technique that enables online analysis or even feedback during processing. Lastly, while the performance of growth model co-refinement and the CNN is similar on sparsely sampled data, the CNN has advantages when analyzing noisy data. The choice of analysis strategy therefore depends strongly on the experimental setup and the number of experiments to be analyzed. Using a weak source with low flux or short integration times results in noisy data and would favor the use of a CNN approach. In applications where the motor movement times of a diffractometer are limiting the time resolution, our sparse sampling approach with fewer data points and fewer motor movements is a viable alternative for speeding up measurements. Both growth model co-refinement and the CNN approach perform similarly with respect to the possible reduction in data points needed and fit errors in this case. Note that both sparse sampling approaches presented here can also deal not just with data collected at random *q* points but also with data collected at one or several fixed *q* points as a function of time. Examples of such growth studies include measurements of the anti-Bragg oscillations at *q* = 1/2*q*_Bragg_, which both our CNN and growth model co-refinement can analyze. However, a single *q* point measured at 80 time steps is below our lower limit of 200 data points for a reasonable accuracy (see Table 1 ▸), so that it is advisable to measure also at other *q* points beyond the anti-Bragg point, *e.g.* at *q* = 1/3*q*_Bragg_ and 1/4*q*_Bragg_ (Kowarik *et al.*, 2009 ▸).

The CNN and *refnx* co-refinement approach allow one to implement models not just for growth processes but also for other time-dependent XRR problems. Kinetic models can, for example, be included as prior information in the CNN via a simulation of the corresponding training data with the kinetic model, the parameters of which become training labels. Alternatively, the kinetic model can be directly incorporated into a *refnx* co-refinement. Note that a CNN can learn about physically sensible solutions not just via the choice of training data, as presented here, but also via a careful choice of a physics-informed loss function, as has recently been demonstrated (Raissi *et al.*, 2019 ▸). Through their flexibility, the CNN and the growth model co-refinement lend themselves to numerous applications in fields that profit from *in situ* and real-time measurements, such as electrochemical changes at interfaces, but the co-refinement approach is also useful for data acquired *e.g.* with different contrasts in NR.

## 5. Conclusion

In this article we have shown the possibilities of the NN and growth model fit approaches for fitting *in situ* XRR measurements using co-refinement of multiple XRR curves. Prior knowledge is implemented in the analysis in the form of a kinetic growth model. This model re-parametrizes the structural evolution with far fewer parameters than independent structural film properties at each time step. The CNN and batch fit directly predict growth model parameters without the need for independent single XRR curve fitting. The co-refinement of many XRR curves enables predictions for sparsely sampled data sets that have insufficient information for single curve fits or single curve NN predictions. We tested our CNN and growth model co-refinement approaches on experimentally measured data where we reduced the number of reflectivity points from 4160 measured reflectivity data points to 200 randomly distributed data points that were sufficient to make correct thin film parameter predictions. This significantly undercuts the minimum number of 1700 data points needed for individual curve fitting. By combining fast data analysis and accurate prediction from a sparse data set, we can go beyond the state of the art in XRR measurements and effectively speed up XRR measurements by almost an order of magnitude. This allows one to analyze sensitive samples without beam damage or increase the throughput in experiments.

Moreover, we tested our NN and growth model co-refinement on data with high photon shot noise, as occurs for short data acquisition times or weak X-ray sources. We showed that our CNN can predict correct structural film properties even from extremely noisy data. In comparison with a conventional independent XRR curve fit, the exposure of the sample can be three orders of magnitude lower and the CNN prediction still corresponds to a real thin film growth scenario. This result will allow significantly faster measurements to be made at synchrotron facilities with high flux or enable the use of lower-brightness laboratory sources for real-time measurements.

The supporting information associated with this article is also available in a GitHub repository: https://github.com/kowarik-lab/XRR-model-based-co-refinement-CNN-refnx.

## Supplementary Material

Jupyter notebooks with the training data generation for the CNN, training of the CNN and the CNN prediction from the full data set, sparsely sampled data set and noisy data set. DOI: 10.1107/S2053273322008051/yr5088sup1.pdf

Co-refinement script with implemented growth model. DOI: 10.1107/S2053273322008051/yr5088sup2.pdf

A file with trained CNN models. DOI: 10.1107/S2053273322008051/yr5088sup3.zip

The experimental data with manually obtained labels. DOI: 10.1107/S2053273322008051/yr5088sup4.zip

A small training data set with the labels. DOI: 10.1107/S2053273322008051/yr5088sup5.zip

Scripts for the training data generation. DOI: 10.1107/S2053273322008051/yr5088sup6.zip

## Figures and Tables

**Table 1:** Limits of data reduction and artificial noise for an acceptable performance of the individual curve fit, growth model co-refinement and CNN predictions

	Sparse data set Minimum number of data points gain: *x* times			Noisy data Minimum count rate at total reflectivity edge gain: *x* times		
	Thickness	SLD	Roughness	Thickness	SLD	Roughness
Benchmark: individual fit	1700	1700	2200	1 × 10^5^	1 × 10^5^	5 × 10^5^
Growth model co-refinement	200/8×	200/8×	200/11×	300/300×	500/200×	1000/500×
CNN	240/7×	200/8×	200/11×	30/3000×	500/200×	10/5 × 10^4^×
