# Supplementary material for: Faster and lower-dose X-ray reflectivity measurements enabled by physics-informed modeling and artificial intelligence co-refinement
Source: J Appl Crystallogr. 2022 Oct 1;55(Pt 5):1305–13. doi: 10.1107/S2053273322008051 (PMC9533750; doi:10.1107/S2053273322008051)
Supplement: Supplementary file 1 [file j-55-01305-sup1.pdf]

JOURNAL OF
APPLIED
CRYSTALLOGRAPHY

Volume 55 (2022)

Supporting information for article:

Faster and lower-dose X-ray reflectivity measurements enabled by physics-informed modeling and artificial intelligence co-refinement

David Mareček, Julian Oberreiter, Andrew Nelson and Stefan Kowarik

<!DOCTYPE html>

CNN parameter prediction from noisy experimental data

We load the experimental data set from "ExperimentalData" file. We reshape and normalize them. We apply noise to the experimental data set (cell 6). We load the CNN model retrained with the noisy data and use it to predict the parameters from noisy data. We renormalise predicted parameters (cell 12) and put them back into the growth model. Based on the parameters we reproduce the thin film growth scenario (thickness and roughness evolution). In the end, we generate new XRR curves based on the predicted film growth scenario (cell 15).

In [1]:
```
import os

#os.environ["CUDA_VISIBLE_DEVICES"]="/gpu:0"

os.environ['TF_CPP_MIN_LOG_LEVEL'] = '1'
os.environ["CUDA_VISIBLE_DEVICES"]="1"

import tensorflow as tf
import keras
```

In [2]:
```
import matplotlib.pylab as plt
import numpy as np
import pickle
import matplotlib

from matplotlib import image
from matplotlib import pyplot
```

In [3]:
```
%matplotlib inline
```

In [4]:
```
from keras.models import load_model, Sequential
from keras.layers import Dense, Dropout, Activation, Dropout, Flatten, BatchNormalization
from keras import metrics
from keras.callbacks import ModelCheckpoint, Callback, ReduceLROnPlateau, CSVLogger
from keras import optimizers
from keras.layers.convolutional import Convolution2D, MaxPooling2D, ZeroPadding2D, Conv2D, AveragePooling2D
from keras.preprocessing.image import ImageDataGenerator
```

In [ ]:
```

```

In [5]:
```
exp_0 = np.load("ExperimentalData/Data_DIP_403K.dat", allow_pickle = True)
exp_1 = np.load("ExperimentalData/Labels_DIP_403K.dat", allow_pickle = True)

print(exp_0.shape,np.min(exp_0))
exp_new = exp_0[1:,:]
print(exp_new.shape)
for i in range(80):
    for j in range(109):
        if exp_new[i,j] >1:
            exp_new[i,j] = 1


origin = tf.reshape(exp_new, [1,80,109,1])
```

```
(81, 109) 1.8177991864864553e-06
(80, 109)
```

In [6]:
```
#NOISE
#cps=100000
target=exp_new
target=np.reshape(target, [8720])

from scipy.stats import poisson
from numpy import inf

noisyr_a = []

A = 1
cps = 5e4

for i in range(A):
    noisyr = poisson.rvs(mu=target*cps, size=8720)/cps
    noisyr = (np.log(noisyr) +20)/(20)
```

```
    noisyr[noisyr == -inf] = 0
    noisyr_a.append(noisyr)

exp_noise = tf.reshape(noisyr_a, [A,80,109,1])

plt.imshow(exp_noise[A-1,:,:,:])
plt.colorbar()
plt.savefig('Noise'+str(cps)+'.svg', format='svg', dpi=600)
plt.clim(1, 0.26)
plt.show()
```

```
/tmp/ipykernel_92111/993230009.py:16: RuntimeWarning: divide by zero encountered in log
  noisyr = (np.log(noisyr) +20)/(20)
```

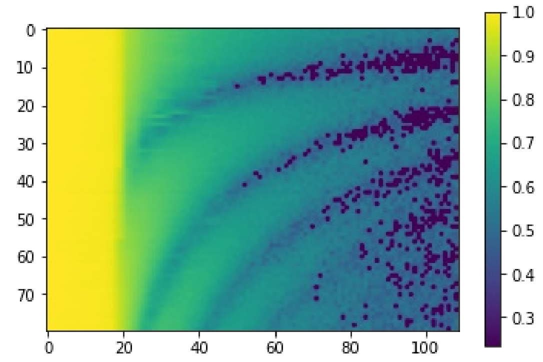

In [7]:

```
model = load_model('conv_models/20220123_Noise_adagrad10')  #Noisy
```

In [8]:

```
print(exp_new)
print(exp_new.shape)
```

```
[[1.00000000e+00 1.00000000e+00 1.00000000e+00 ... 9.86600280e-05
  9.49243619e-05 9.33801098e-05]
 [9.85083065e-01 9.80482403e-01 9.75350253e-01 ... 9.37923487e-05
  9.05024970e-05 8.91397193e-05]
 [9.96211299e-01 9.92058570e-01 9.87423621e-01 ... 7.22183172e-05
  6.89906736e-05 6.76632093e-05]
 ...
 [9.93256188e-01 9.94183522e-01 9.95224164e-01 ... 3.14493386e-05
  3.13600599e-05 3.13221996e-05]
 [9.99999993e-01 9.89167865e-01 9.77163667e-01 ... 3.03059528e-05
  2.94385907e-05 2.90775811e-05]
 [9.91398035e-01 9.95035376e-01 9.99128980e-01 ... 2.94854218e-05
  2.79022571e-05 2.72555312e-05]]
(80, 109)
```

In [9]:

```
labels_predicted = model.predict(exp_noise)
curve_number = np.linspace(0, 80, 80)
```

model = tf.keras.models.load_model('my_model_image')

pip install tqdm

In [10]:

```
from numpy import exp, array, insert
from math import log, sqrt, e


import numpy as np
from scipy import integrate

import matplotlib.pyplot as plt
import matplotlib as mpl
from matplotlib.widgets import CheckButtons, Cursor

from tqdm import tqdm

print('Module imports finished')

##############################################################################
###############       Trofimov-model for layer coverages         ###############
##############################################################################
```

```
def trofimov(X, t, *args):
    """
    X: list of differential equations to solve
    t: independent variable
    args: Xc, R123
        Xc: list of theta criticals
        R123 = R1,R2,R3,R: growth rates for different layers
    """

    def Xi(Theta, ThetaC):
        """ calculate Xi_n for Theta_n and ThetaC_n """

        if Theta > ThetaC:
            if Theta >= 1: return 1 #catch rounding errors, math domain error
            if Theta <= 0: return 0
            if ThetaC >= 1: return 1 #catch rounding errors, math domain error
            if ThetaC <= 0: return 0
            expo1 = sqrt(-log(1-Theta))
            expo2 = sqrt(-log(1-ThetaC))
            return 1 - exp(-(expo1-expo2)*(expo1-expo2))
        else: return 0

    Xc, R123 = args
    R1, R2, R3, R = R123
    N = len(Xc)

    # Rate equations for different rates for the first four ML
    eqn1 = 0
    if X[0] < 0.99999999:
        eqn1 = R1 * (1-X[0]) + R2 * (X[0] - Xi(X[0],Xc[0]))

    eqn2 = R2 * (Xi(X[0],Xc[0]) - X[1]) + R * (X[1] - Xi(X[1],Xc[1]))

    eqn3 = R3 * (Xi(X[1],Xc[1]) - X[2]) + R * (X[2] - Xi(X[2],Xc[2]))

    eqn = [R*(Xi(X[n-2],Xc[n-2]) - Xi(X[n-1],Xc[n-1])) for n in range(4,N+1)]

    return array([eqn1, eqn2, eqn3] + eqn)


################################################################################
###############     Functions for calculations      ###############
################################################################################

def calc_all(t, R123, ThetaC):
    """ Calculate coverages, thickness, roughness, reflectivity """

    X0 = [0]*N                          # layer-coverages for t=0

    X = integrate.odeint(trofimov, X0, t, args=(ThetaC,R123), mxstep=500)[:]
    coverages = np.transpose(X)     # Coverages in a.u.
    thickness = sum(coverages)      # Total film thickness in a.u.
    # Film roughness in [ML]
    roughness = np.sqrt(abs(sum([(coverages[i] - coverages[i+1])
                              *(i+1 - thickness)**2 for i in range(N-1)])))

    return X, coverages, thickness, roughness
```

Module imports finished

In [11]:

```
label_pred = labels_predicted[:,:]
```

In [12]:

```
G1=label_pred[:,0]+0.1 #renormalized

G2 = label_pred[:,1] + 0.2

G3 = label_pred[:,2] + 0.2

G4 = label_pred[:,3]/0.55


a=label_pred[:,4]/1.5

b=(label_pred[:,5]*1.3)-0.5

c=label_pred[:,6]/2

d = (label_pred[:,7]*7.5)-7.1

g = label_pred[:,8]/9

sld=label_pred[:,9]*2e15
```

```python
x = np.linspace(0, 35, 35)
t = np.arange(0, 16, 0.2).tolist()
z1 = np.zeros((35,0))
N=35
roughness_m=np.zeros((80,0))
thickness_m=np.zeros((80,0))
label=[]

for i in range(A):
    f = 0
    z = a[i]*(((0.5*np.tanh(-0.5*(x+d[i]))+0.5))/((0.5*np.tanh(-0.5*d[i])+0.5)))
    for j in range (35):
        if z[j] > c[i]:
            z[j] = z[j]
            f = j
        else:
            z[j] = (c[i]-g[i])*e**(-b[i]*x[j-f])+g[i]

    z = z.reshape(N,1)
    z1 = np.append(z1, z, axis=1)


#multiple grow example
for i in range(A):
    gr = [G1[i], G2[i], G3[i], G4[i]]
    thetacrit = z1[:,i]
    out = calc_all(t,gr,thetacrit)
    Roug = out[3].reshape(80,1)
    thick = out[2].reshape(80,1)
    thickness_m = np.append(thickness_m, thick, axis=1)
    roughness_m = np.append(roughness_m, Roug, axis=1)
    lab = G1[i], G2[i], G3[i], G4[i], a[i], b[i], c[i], d[i], sld[i]
    label.append(lab)
```

In [13]:

```python
# print(thickness_m)
# print(roughness_m)
# print(sld)
# from numpy import savetxt
```

In [14]:

```python
import numpy as np
from data_handling import make_reflectivity_curves
import matplotlib.pyplot as plt
```

In [15]:

```python
q_values = np.linspace(0.01, 0.14, 109)
q_values = q_values * 1e10

n_samples = 1
training_data_output = np.zeros([len(q_values), 0])
```

In [16]:

```python
from numpy import append
for i in tqdm(range (1)):


    for x in range (0,80):

        thickness = np.array([thickness_m[x,i]*16.6*1e-10, 10.86*1e-10, 0*1e-10])
        repetitions = n_samples
        thicknesses = np.tile(thickness, (repetitions, 1))
        roughness = np.array([roughness_m[x,i]*16.6*1e-10, 5.612*1e-10, 1*1e-11])
        repetitions = n_samples
        roughnesses = np.tile(roughness, (repetitions, 1))

        SLD = np.array([sld[i], 1.903*1e15, 1.977*1e+15]) ###(oxide,bulk)
        repetitions = n_samples
        SLDs = np.tile(SLD, (repetitions, 1))
        training_reflectivity = make_reflectivity_curves(
                q_values, thicknesses, roughnesses, SLDs, n_samples)

        training_data_output = append(training_data_output, training_reflectivity, axis=1)
```

```
100%|██████████| 1/1 [00:00<00:00,  2.42it/s]
```

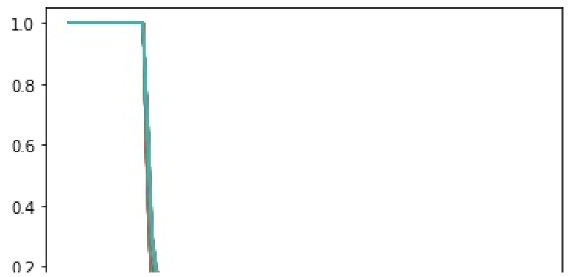

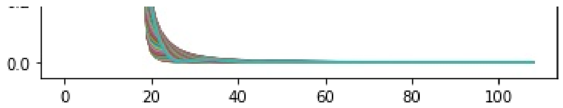

In [17]:

```
T_v_n = training_data_output
T_v = np.log(training_data_output)


Training_data_v = (T_v+18.420680743952367)/(4.440892098500625e-16+18.420680743952367)
Training_data_v_n = (T_v_n-np.min(T_v_n))/(np.max(T_v_n)-np.min(T_v_n))

Training_data_v_nn = e**(Training_data_v)

T_va = np.transpose(Training_data_v)
T_va_n = np.transpose(T_v_n)
Training_data_v_nn = np.transpose(Training_data_v_nn)

print(T_va.shape)

T_v = tf.reshape(T_va, [1, 80, 109,1])

print(np.max(T_v_n), np.min(T_v_n))
```

```
(80, 109)
1.0000000000000004 1.9744213050869773e-07
```

In [18]:

```
T_xv = tf.reshape(np.log(origin), [1, 80, 109])
f, axarr = plt.subplots(1,2, figsize=(12,3.7))

pos0 = axarr[1].imshow(T_xv[-1,:,:], extent=[0.01,0.14,265.6,0], aspect="auto")
pos1 = axarr[0].imshow(T_v[-1,:,:], extent=[0.01,0.14,265.6,0], aspect="auto")

plt.colorbar(pos1, ax=axarr[1])
plt.colorbar(pos1, ax=axarr[0])

axarr[1].set_title('Experimental data')
axarr[0].set_title('Prediction')

plt.show()
print(label_pred[0,:])
```

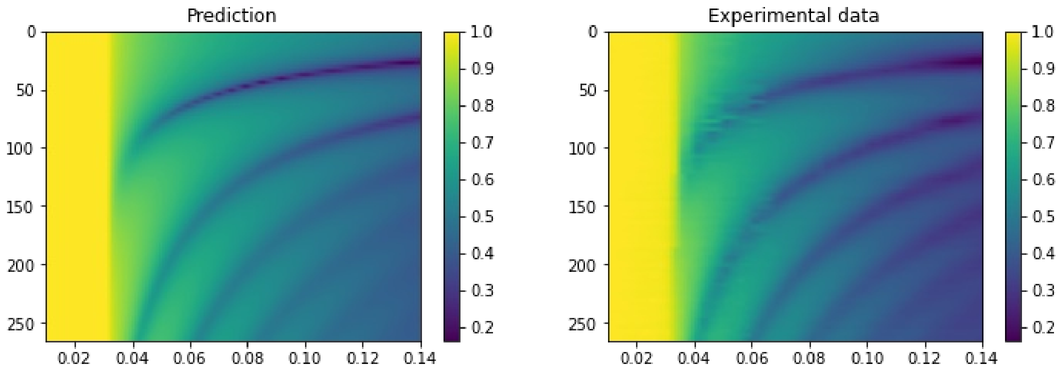


```
[0.9320894  0.49077207 0.5947011  0.58034945 0.4050942  0.87682843
 0.34435174 0.36245757 0.34967402 0.53787196]
```

In [ ]:

In [ ]:

Loading [MathJax]/jax/output/CommonHTML/fonts/TeX/fontdata.js

<!DOCTYPE html>

CNN parameter prediction from sparsely sampled experimental data

We load the experimental data set from "ExperimentalData" file. We reshape and normalize them. We define "drop" function and apply it to the experimental data set (cell 6). We load the CNN model retrained with the dropout data and use it to predict the sparsely sampled dataset parameters. We renormalise predicted parameters (cell 11) and put them back into the growth model. Based on the parameters we reproduce the thin film growth scenario (thickness and roughness evolution). In the end, we generate new XRR curves based on the predicted film growth scenario (cell 15).

In [1]:

```
import os

os.environ['TF_CPP_MIN_LOG_LEVEL'] = '1'
os.environ["CUDA_VISIBLE_DEVICES"]="0"

import tensorflow as tf
import keras
%matplotlib inline
```

In [2]:

```
import matplotlib.pylab as plt
import numpy as np
import pickle
import matplotlib

from matplotlib import image
from matplotlib import pyplot
```

In [3]:

```
from tensorflow import keras

from keras.models import load_model, Sequential
from keras.layers import Dense, Dropout, Activation, Dropout, Flatten, BatchNormalization
from keras import metrics
from keras.callbacks import ModelCheckpoint, Callback, ReduceLROnPlateau, CSVLogger
from keras import optimizers
from keras.layers.convolutional import Convolution2D, MaxPooling2D, ZeroPadding2D, Conv2D, AveragePooling2D
from keras.preprocessing.image import ImageDataGenerator
```

In [ ]:

```

```

In [4]:

```
exp_0 = np.load("ExperimentalData/Data_DIP_403K.dat", allow_pickle = True)
exp_1 = np.load("ExperimentalData/Labels_DIP_403K.dat", allow_pickle = True)

exp_new = exp_0[1:,:]
print(exp_new.shape)

exp_log = np.log(exp_new)

exp_norm = (exp_log+20)/(20)
exp_norm_reshape = tf.reshape(exp_norm, [1,80,109,1])

plt.rcParams['font.size'] = '14'
plt.figure(figsize=(7, 5), dpi=80)
plt.plot(exp_0[0,:], (exp_new[75,:]*1e12))
plt.xlabel('q[1/Å]')
plt.ylabel('Normalized counts [Å]')
plt.show()
```

```
(80, 109)
```

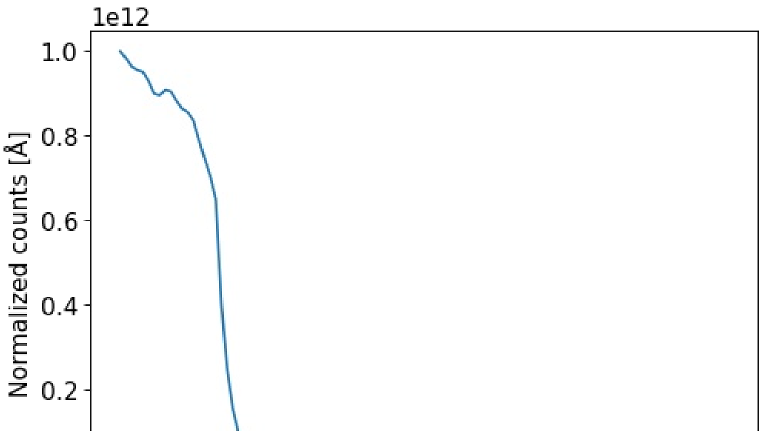

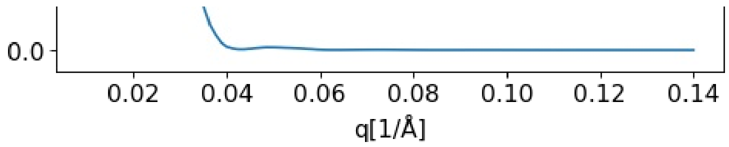

In [5]:
```
def drop(x,drp):
    layer = tf.keras.layers.Dropout(drp, input_shape=(2,))
    outputs = layer(x, training=True)
    return outputs
```

In [6]:
```
drp=0.8
T_drop = drop(exp_norm_reshape, drp)

plt.imshow(T_drop[0,:,:], extent=[0.01,0.14,265.6,0], aspect="auto")
plt.xlabel('q[1/Å]')
plt.ylabel('Thickness [Å]')
plt.savefig('or_drop0999.png')
plt.show()
```

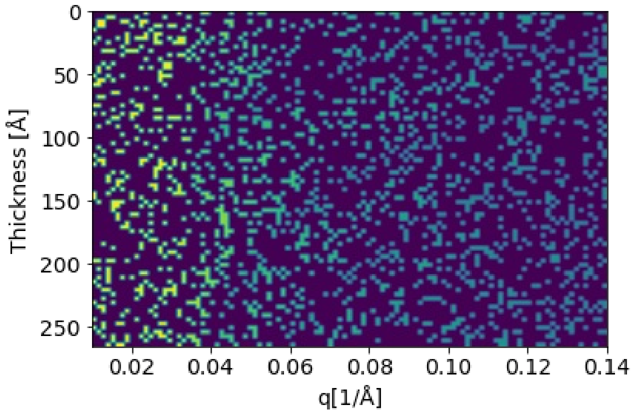

In [7]:
```
model = load_model('conv_models/20211109_drop_adam2.h5')
```

In [8]:
```
labels_predicted = model.predict(T_drop)
curve_number = np.linspace(0, 80, 80)
print(labels_predicted)
```

```
[[0.966018   0.5320316  0.6316903  0.75328535 0.6653125  0.6108159
  0.66849583 0.24517378 0.6877373  0.5977231 ]]
```

In [9]:
```
from numpy import exp, array, insert
from math import log, sqrt, e


import numpy as np
from scipy import integrate

import matplotlib.pyplot as plt
import matplotlib as mpl
from matplotlib.widgets import CheckButtons, Cursor

from tqdm import tqdm

print('Module imports finished')

###############################################################################
###############      Trofimov-model for layer coverages         ###############
###############################################################################

def trofimov(X, t, *args):
    """
    X: list of differential equations to solve
    t: independent variable
    args: Xc, R123
        Xc: list of theta criticals
        R123 = R1,R2,R3,R: growth rates for different layers
    """
```

```
    def Xi(Theta, ThetaC):
        """ calculate Xi_n for Theta_n and ThetaC_n """

        if Theta > ThetaC:
            if Theta >= 1: return 1 #catch rounding errors, math domain error
            if Theta <= 0: return 0
            if ThetaC >= 1: return 1 #catch rounding errors, math domain error
            if ThetaC <= 0: return 0
            expo1 = sqrt(-log(1-Theta))
            expo2 = sqrt(-log(1-ThetaC))
            return 1 - exp(-(expo1-expo2)*(expo1-expo2))
        else: return 0

    Xc, R123 = args
    R1, R2, R3, R = R123
    N = len(Xc)

    # Rate equations for different rates for the first four ML
    eqn1 = 0
    if X[0] < 0.99999999:
        eqn1 = R1 * (1-X[0]) + R2 * (X[0] - Xi(X[0],Xc[0]))

    eqn2 = R2 * (Xi(X[0],Xc[0]) - X[1]) + R * (X[1] - Xi(X[1],Xc[1]))

    eqn3 = R3 * (Xi(X[1],Xc[1]) - X[2]) + R * (X[2] - Xi(X[2],Xc[2]))

    eqn = [R*(Xi(X[n-2],Xc[n-2]) - Xi(X[n-1],Xc[n-1])) for n in range(4,N+1)]

    return array([eqn1, eqn2, eqn3] + eqn)


################################################################################
###############     Functions for calculations      ###############
################################################################################

def calc_all(t, R123, ThetaC):
    """ Calculate coverages, thickness, roughness, reflectivity """

    X0 = [0]*N                        # layer-coverages for t=0

    X = integrate.odeint(trofimov, X0, t, args=(ThetaC,R123), mxstep=500)[:]
    coverages = np.transpose(X)     # Coverages in a.u.
    thickness = sum(coverages)      # Total film thickness in a.u.
    # Film roughness in [ML]
    roughness = np.sqrt(abs(sum([(coverages[i] - coverages[i+1])
                          *(i+1 - thickness)**2 for i in range(N-1)])))

    return X, coverages, thickness, roughness
```

Module imports finished

In [10]:

```
label_pred = labels_predicted[-10:,:]
print(label_pred)
```

[[0.966018   0.5320316 0.6316903 0.75328535 0.6653125 0.6108159
  0.66849583 0.24517378 0.6877373 0.5977231]]

In [11]:

```
G1=label_pred[:,0]+0.1 #renormalized

G2 = label_pred[:,1] + 0.2

G3 = label_pred[:,2] + 0.2

G4 = label_pred[:,3]/0.7


a=label_pred[:,4]/1.5

b=(label_pred[:,5]*1.5)-0.5

c=label_pred[:,6]/3

d = (label_pred[:,7]*7)-6.1

g = label_pred[:,8]/20

print("d is ", d)

sld=label_pred[:,9]*1.5e15

x = np.linspace(0, 35, 35)
t = np.arange(0, 16, 0.2).tolist()
```

```
z1 = np.zeros((35,0))
N=35
roughness_m=np.zeros((80,0))
thickness_m=np.zeros((80,0))
label=[]

for i in tqdm(range(1)):
    f = 0
    z = a[i]*(((0.5*np.tanh(-0.5*(x+d[i]))+0.5))/((0.5*np.tanh(-0.5*d[i])+0.5)))
    for j in range (35):
        if z[j] > c[i]:
            z[j] = z[j]
            f = j
        else:
            z[j] = (c[i]-g[i])*e**(-b[i]*x[j-f])+g[i]

    z = z.reshape(N,1)
    z1 = np.append(z1, z, axis=1)

plt.plot(x,z, '.')
plt.show()

for i in tqdm(range(1)):
    gr = [G1[i], G2[i], G3[i], G4[i]]
    thetacrit = z1[:,i]
    out = calc_all(t,gr,thetacrit)
    Roug = out[3].reshape(80,1)
    thick = out[2].reshape(80,1)
    thickness_m = np.append(thickness_m, thick, axis=1)
    roughness_m = np.append(roughness_m, Roug, axis=1)
    lab = G1[i], G2[i], G3[i], G4[i], a[i], b[i], c[i], d[i], sld[i]
    label.append(lab)
```

```
d is  [-4.3837833]
```

```
100%|██████████| 1/1 [00:00<00:00, 1253.90it/s]
```

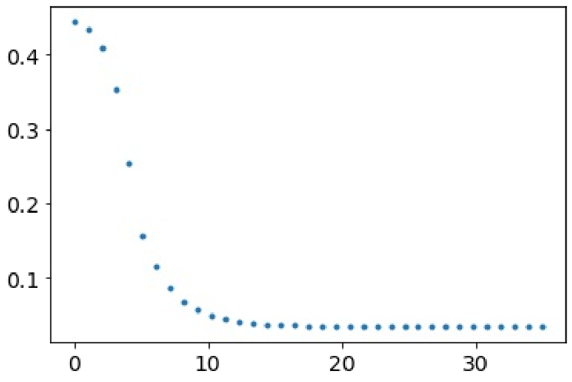


```
100%|██████████| 1/1 [00:07<00:00,  7.00s/it]
```

In [12]:

```
# print(thickness_m)
# print(roughness_m)
# print(sld)
# from numpy import savetxt
# np.savetxt("thickness"+str(drp)+".dat", thickness_m, delimiter =",")
# np.savetxt("roughness"+str(drp)+".dat", roughness_m, delimiter =",")
# np.savetxt("sld"+str(drp)+".dat", sld, delimiter =",")
```

In [13]:

```
import numpy as np
from data_handling import make_reflectivity_curves
import matplotlib.pyplot as plt
```

In [14]:

```
q_values = np.linspace(0.01, 0.14, 109)
q_values = q_values * 1e10

n_samples = 1
training_data_output = np.zeros([len(q_values), 0])
```

In [15]:

```
from numpy import append
for i in tqdm(range (1)):


    for x in range (0,80):
```

```
        thickness = np.array([thickness_m[x,i]*16.6*1e-10, 10.86*1e-10, 0*1e-10])
        repetitions = n_samples
        thicknesses = np.tile(thickness, (repetitions, 1))

        roughness = np.array([roughness_m[x,i]*16.6*1e-10, 5.612*1e-10, 1*1e-11])
        repetitions = n_samples
        roughnesses = np.tile(roughness, (repetitions, 1))

        SLD = np.array([sld[i], 1.903*1e15, 1.977*1e+15])
        repetitions = n_samples
        SLDs = np.tile(SLD, (repetitions, 1))

        training_reflectivity = make_reflectivity_curves(
                q_values, thicknesses, roughnesses, SLDs, n_samples)

        training_data_output = append(training_data_output, training_reflectivity, axis=1)
```

```
100%|██████████| 1/1 [00:00<00:00,  3.14it/s]
```

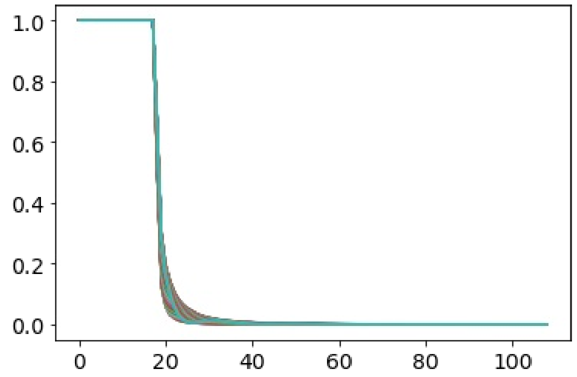

In [16]:

```
T_v_n = training_data_output
T_v = np.log(training_data_output)

Training_data_v = (T_v+20)/(20)
Training_data_v_n = (T_v_n-np.min(T_v_n))/(np.max(T_v_n)-np.min(T_v_n))

Training_data_v_nn = e**(Training_data_v)

T_va = np.transpose(Training_data_v)
T_va_n = np.transpose(T_v_n)
Training_data_v_nn = np.transpose(Training_data_v_nn)

print(T_va.shape)

T_v = tf.reshape(T_va, [1, 80, 109,1])

print(np.max(T_v_n), np.min(T_v_n))
```

```
(80, 109)
1.0000000000000004 2.9938779538050106e-06
```

In [17]:

```
T_xv = tf.reshape(exp_norm_reshape[-10:,:], [1, 80, 109])
#subplot(r,c) provide the no. of rows and columns
f, axarr = plt.subplots(1,2, figsize=(12,3.7))

pos0 = axarr[1].imshow(T_xv[-1,:,:], extent=[0.01,0.14,265.6,0], aspect="auto")
plt.colorbar(pos0, ax=axarr[1])

pos1 = axarr[0].imshow(T_v[-1,:,:], extent=[0.01,0.14,265.6,0], aspect="auto")
plt.colorbar(pos1, ax=axarr[0])
axarr[1].set_title('Experimental data')
axarr[0].set_title('Prediction')

plt.show()
print(label_pred[-10:,:])
```

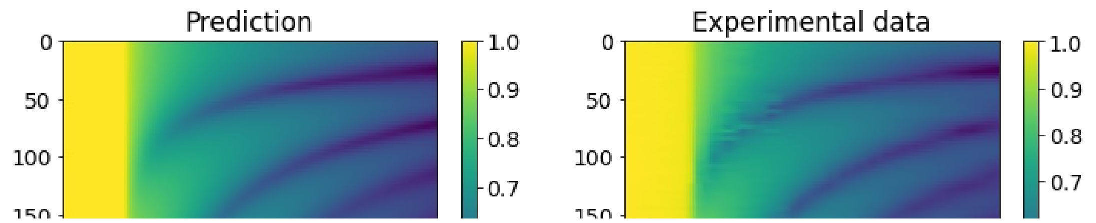

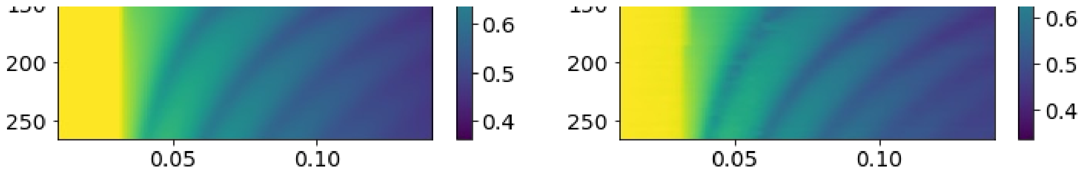


```
[[0.966018   0.5320316  0.6316903  0.75328535 0.6653125  0.6108159
  0.66849583 0.24517378 0.6877373  0.5977231 ]]
```

Loading [MathJax]/jax/output/CommonHTML/fonts/TeX/fontdata.js

<!DOCTYPE html>

CNN parameter prediction from the experimental data

We load the experimental data set from "ExperimentalData" file. We reshape and normalize them. We load the CNN model trained on the synthetic data and use it to predict the parameters. We renormalise predicted parameters (cell 12) and put them back into the growth model. Based on the parameters we reproduce the thin film growth scenario (thickness and roughness evolution). In the end, we generate new XRR curves based on the predicted thin film growth scenario (cell 16).

In [1]:

```
import os

os.environ['TF_CPP_MIN_LOG_LEVEL'] = '1'
os.environ["CUDA_VISIBLE_DEVICES"]="0"

import tensorflow as tf
import keras
%matplotlib inline
```

In [2]:

```
import matplotlib.pylab as plt
import numpy as np
import pickle
import matplotlib

from matplotlib import image
from matplotlib import pyplot
```

In [3]:

```
from tensorflow import keras

from keras.models import load_model, Sequential
from keras.layers import Dense, Dropout, Activation, Dropout, Flatten, BatchNormalization
from keras import metrics
from keras.callbacks import ModelCheckpoint, Callback, ReduceLROnPlateau, CSVLogger
from keras import optimizers
from keras.layers.convolutional import Convolution2D, MaxPooling2D, ZeroPadding2D, Conv2D, AveragePooling2D
from keras.preprocessing.image import ImageDataGenerator
```

In [ ]:

```

```

In [4]:

```
exp_0 = np.load("ExperimentalData/Data_DIP_403K.dat", allow_pickle = True)
exp_1 = np.load("ExperimentalData/Labels_DIP_403K.dat", allow_pickle = True)

exp_new = exp_0[1:,:]
print(exp_new.shape)

exp_log = np.log(exp_new)

exp_norm = (exp_log+20)/(20)
exp_norm_reshape = tf.reshape(exp_norm, [1,80,109,1])

plt.rcParams['font.size'] = '14'
plt.figure(figsize=(7, 5), dpi=80)
plt.plot(exp_0[0,:], (exp_new[75,:]*1e12))
plt.xlabel('q[1/Å]')
plt.ylabel('Normalized counts [Å]')
plt.show()
```

```
(80, 109)
```

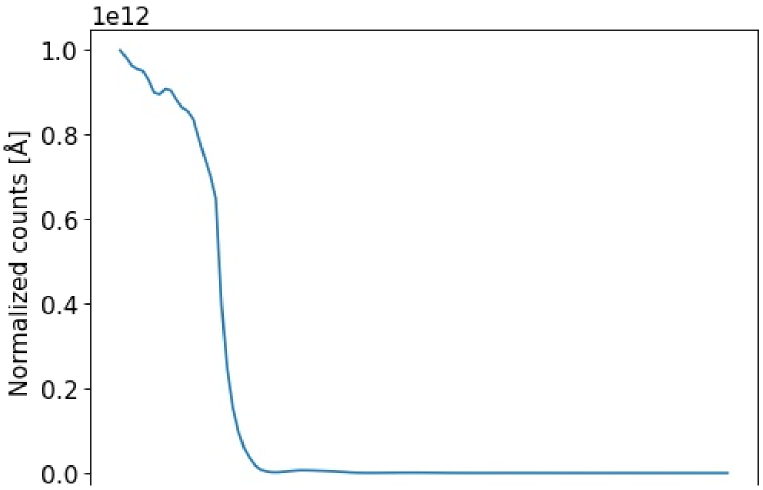

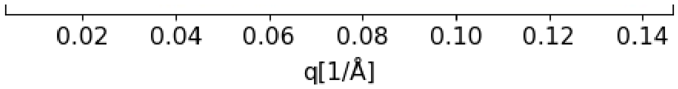

In [5]:

```
def drop(x,drp):
    layer = tf.keras.layers.Dropout(drp, input_shape=(2,))
    outputs = layer(x, training=True)
    return outputs
```

In [6]:

```
drp=0
T_drop = drop(exp_norm_reshape, drp)

plt.imshow(T_drop[0,:,:], extent=[0.01,0.14,265.6,0], aspect="auto")
plt.xlabel('q[1/Å]')
plt.ylabel('Thickness [Å]')
plt.savefig('or_drop0999.png')
plt.show()
```

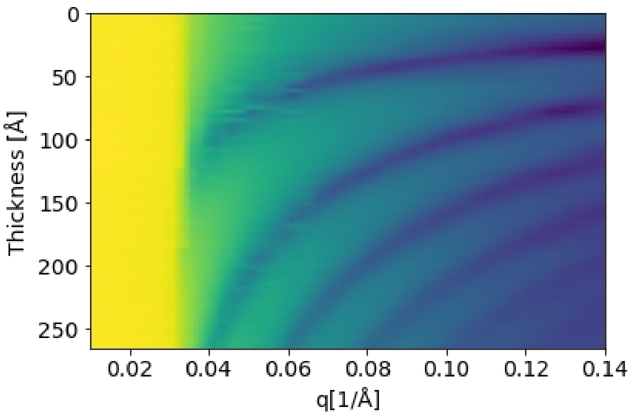

In [7]:

```
model = load_model('conv_models/20211021_exp_adagrad')
```

In [8]:

```
labels_predicted = model.predict(T_drop)
curve_number = np.linspace(0, 80, 80)
print(labels_predicted)
```

```
[[0.8138727  0.7360466  0.8208365  0.7702775  0.49645594 0.54666185
  0.60701007 0.02078663 0.82352495 0.50935036]]
```

In [9]:

```
from numpy import exp, array, insert
from math import log, sqrt, e


import numpy as np
from scipy import integrate

import matplotlib.pyplot as plt
import matplotlib as mpl
from matplotlib.widgets import CheckButtons, Cursor

from tqdm import tqdm

print('Module imports finished')

################################################################################
###############      Trofimov-model for layer coverages         ###############
################################################################################

def trofimov(X, t, *args):
    """
    X: list of differential equations to solve
    t: independent variable
    args: Xc, R123
        Xc: list of theta criticals
        R123 = R1,R2,R3,R: growth rates for different layers
    """

    def Xi(Theta, ThetaC):
```

```python
        """ calculate Xi_n for Theta_n and ThetaC_n """

        if Theta > ThetaC:
            if Theta >= 1: return 1 #catch rounding errors, math domain error
            if Theta <= 0: return 0
            if ThetaC >= 1: return 1 #catch rounding errors, math domain error
            if ThetaC <= 0: return 0
            expo1 = sqrt(-log(1-Theta))
            expo2 = sqrt(-log(1-ThetaC))
            return 1 - exp(-(expo1-expo2)*(expo1-expo2))
        else: return 0

    Xc, R123 = args
    R1, R2, R3, R = R123
    N = len(Xc)

    # Rate equations for different rates for the first four ML
    eqn1 = 0
    if X[0] < 0.99999999:
        eqn1 = R1 * (1-X[0]) + R2 * (X[0] - Xi(X[0],Xc[0]))

    eqn2 = R2 * (Xi(X[0],Xc[0]) - X[1]) + R * (X[1] - Xi(X[1],Xc[1]))

    eqn3 = R3 * (Xi(X[1],Xc[1]) - X[2]) + R * (X[2] - Xi(X[2],Xc[2]))

    eqn = [R*(Xi(X[n-2],Xc[n-2]) - Xi(X[n-1],Xc[n-1])) for n in range(4,N+1)]

    return array([eqn1, eqn2, eqn3] + eqn)


################################################################################
###############     Functions for calculations      ###############
################################################################################

def calc_all(t, R123, ThetaC):
    """ Calculate coverages, thickness, roughness, reflectivity """

    X0 = [0]*N                         # layer-coverages for t=0

    X = integrate.odeint(trofimov, X0, t, args=(ThetaC,R123), mxstep=500)[:]
    coverages = np.transpose(X)     # Coverages in a.u.
    thickness = sum(coverages)      # Total film thickness in a.u.
    # Film roughness in [ML]
    roughness = np.sqrt(abs(sum([(coverages[i] - coverages[i+1])
                          *(i+1 - thickness)**2 for i in range(N-1)])))

    return X, coverages, thickness, roughness
```

```
Module imports finished
```

In [10]:

```python
label_pred = labels_predicted[-10:,:]

print(label_pred)
```

```
[[0.8138727  0.7360466  0.8208365  0.7702775  0.49645594 0.54666185
  0.60701007 0.02078663 0.82352495 0.50935036]]
```

In [11]:

```python
G1=label_pred[:,0]+0.1 #renormalized

G2 = label_pred[:,1] + 0.2

G3 = label_pred[:,2] + 0.2

G4 = label_pred[:,3]/0.7

a=label_pred[:,4]/1.5

b=(label_pred[:,5]*1.5)-0.5

c=label_pred[:,6]/3

d = (label_pred[:,7]*7)-6.1

g = label_pred[:,8]/20

sld=label_pred[:,9]*1.5e15

x = np.linspace(0, 35, 35)
t = np.arange(0, 16, 0.2).tolist()
z1 = np.zeros((35,0))
N=35
roughness_m=np.zeros((80,0))
```

```
thickness_m=np.zeros((80,0))
label=[]

for i in tqdm(range(1)):
    f = 0
    z = a[i]*(((0.5*np.tanh(-0.5*(x+d[i]))+0.5))/((0.5*np.tanh(-0.5*d[i])+0.5)))
    for j in range (35):
        if z[j] > c[i]:
            z[j] = z[j]
            f = j
        else:
            z[j] = (c[i]-g[i])*e**(-b[i]*x[j-f])+g[i]

    z = z.reshape(N,1)
    z1 = np.append(z1, z, axis=1)

plt.plot(x,z, '.')
plt.show()

for i in tqdm(range(1)):
    gr = [G1[i], G2[i], G3[i], G4[i]]
    thetacrit = z1[:,i]
    out = calc_all(t,gr,thetacrit)
    Roug = out[3].reshape(80,1)
    thick = out[2].reshape(80,1)
    thickness_m = np.append(thickness_m, thick, axis=1)
    roughness_m = np.append(roughness_m, Roug, axis=1)
    lab = G1[i], G2[i], G3[i], G4[i], a[i], b[i], c[i], d[i], sld[i]
    label.append(lab)
```

```
100%|██████████| 1/1 [00:00<00:00, 1606.40it/s]
```

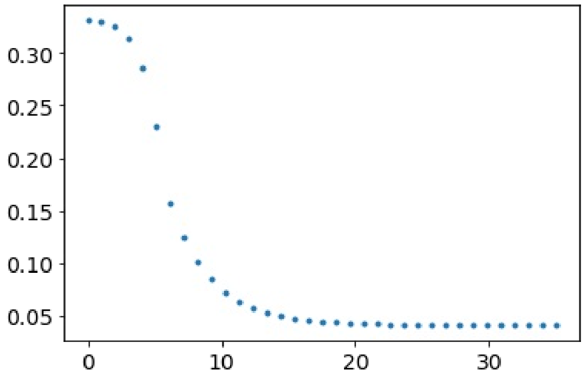


```
100%|██████████| 1/1 [00:01<00:00,  1.82s/it]
```

In [12]:
```
# print(thickness_m)
# print(roughness_m)
# print(sld)
```

In [13]:
```
import numpy as np
from data_handling import make_reflectivity_curves
import matplotlib.pyplot as plt
```

In [14]:
```
q_values = np.linspace(0.01, 0.14, 109)
q_values = q_values * 1e10

n_samples = 1
training_data_output = np.zeros([len(q_values), 0])
```

In [15]:
```
from numpy import append
for i in tqdm(range (1)):


    for x in range (0,80):

        thickness = np.array([thickness_m[x,i]*16.6*1e-10, 10.86*1e-10, 0*1e-10])
        repetitions = n_samples
        thicknesses = np.tile(thickness, (repetitions, 1))

        roughness = np.array([roughness_m[x,i]*16.6*1e-10, 5.612*1e-10, 1*1e-11])
        repetitions = n_samples
        roughnesses = np.tile(roughness, (repetitions, 1))

        SLD = np.array([sld[i], 1.903*1e15, 1.977*1e+15])
```

```
        repetitions = n_samples
        SLDs = np.tile(SLD, (repetitions, 1))

        training_reflectivity = make_reflectivity_curves(
                q_values, thicknesses, roughnesses, SLDs, n_samples)

        training_data_output = append(training_data_output, training_reflectivity, axis=1)
```

```
100%|██████████| 1/1 [00:00<00:00,  2.45it/s]
```

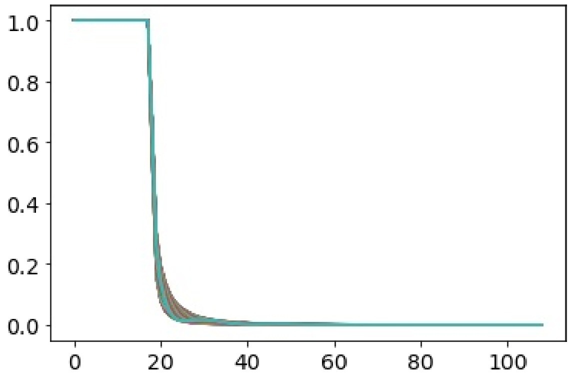

In [16]:

```
T_v_n = training_data_output
T_v = np.log(training_data_output)

Training_data_v = (T_v+20)/(20)
Training_data_v_n = (T_v_n-np.min(T_v_n))/(np.max(T_v_n)-np.min(T_v_n))

Training_data_v_nn = e**(Training_data_v)

T_va = np.transpose(Training_data_v)
T_va_n = np.transpose(T_v_n)
Training_data_v_nn = np.transpose(Training_data_v_nn)

print(T_va.shape)

T_v = tf.reshape(T_va, [1, 80, 109,1])

print(np.max(T_v_n), np.min(T_v_n))
```

```
(80, 109)
1.0000000000000004 8.638981116756268e-06
```

In [17]:

```
T_xv = tf.reshape(exp_norm_reshape[-10:,:], [1, 80, 109])
f, axarr = plt.subplots(1,2, figsize=(12,3.7))

pos0 = axarr[1].imshow(T_xv[-1,:,:], extent=[0.01,0.14,265.6,0], aspect="auto")
pos1 = axarr[0].imshow(T_v[-1,:,:], extent=[0.01,0.14,265.6,0], aspect="auto")

plt.colorbar(pos1, ax=axarr[1])
plt.colorbar(pos1, ax=axarr[0])

axarr[1].set_title('Experimental data')
axarr[0].set_title('Prediction')

plt.show()
print(label_pred[0,:])
```

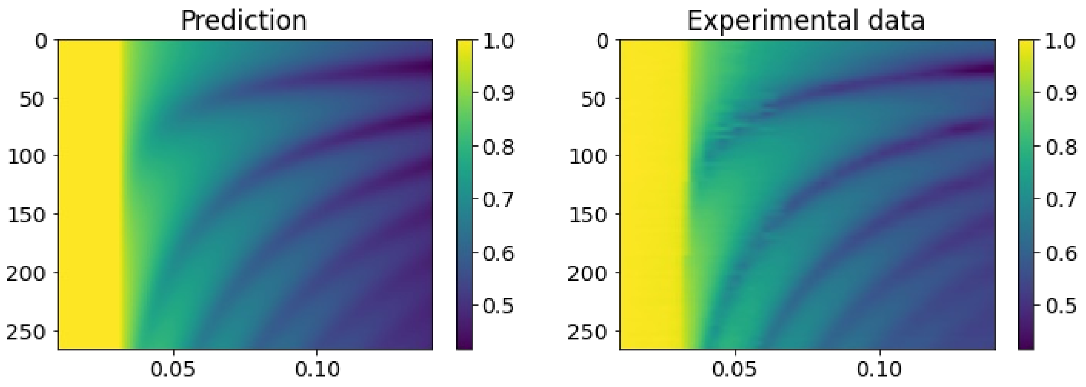


```
[0.8138727  0.7360466  0.8208365  0.7702775  0.49645594 0.54666185
```

```
0.60701007 0.02078663 0.82352495 0.50935036]
```

```
In [ ]:
```

Loading [MathJax]/jax/output/CommonHTML/fonts/TeX/fontdata.js

<!DOCTYPE html>

Implementation of the artificial noise into the dataset

In this script, we load the normalised generated data and corresponding labels (already normalized). We apply a various noise levels (cps) and safe data and the labels.

In [1]:

```
import os
os.environ['TF_CPP_MIN_LOG_LEVEL'] = '1'
os.environ["CUDA_VISIBLE_DEVICES"]="0"

import tensorflow as tf
print("Num GPUs Available: ", len(tf.config.experimental.list_physical_devices('GPU')))
import keras
%matplotlib inline
```

```
Num GPUs Available:  1
```

In [2]:

```
import matplotlib.pylab as plt
import numpy as np
import pickle
from tqdm import tqdm
import random

from matplotlib import image
from matplotlib import pyplot
from matplotlib.pyplot import figure
```

In [ ]:

```

```

In [3]:

```
T = np.load("data_Norm.npy")

T = np.reshape(T, [50, 80*109])

Labels = np.load('Label_Norm.npy', allow_pickle=True)

print(T.shape)
print(Labels.shape)

label50=[]
for i in tqdm(range (1)):
    label50=np.append(label50,Labels)

label50 = np.reshape(label50, [50, 10])
print(label50.shape)


np.save("training_Label_Noise.npy", label50, allow_pickle=True)
```

```
(50, 8720)
(50, 10)
100%|██████████| 1/1 [00:00<00:00, 7145.32it/s]
(50, 10)
```

In [4]:

```
target=T
#print(target)

from scipy.stats import poisson
from numpy import inf


#generate random values from Poisson distribution with mean=3 and sample size=1
poisson.rvs(mu=3, size=1)

#noisytarget reflectivity values
ntarget = []

numberofimages = 50
noisyr =[]
```

```
for a in tqdm(range (numberofimages)):
    cps = random.choice([10,20,50,100,200,400,1000,2000,4000,10000,20000,40000,100000,200000,400000,1000000,2000
    noisyr = poisson.rvs(mu=target[a,:]*cps, size=8720)/cps
    noisyr = (np.log(noisyr) +20)/(20)
    #noisyr[noisyr == -inf] = 0
    ntarget.append(noisyr)
```

```
  0%|          | 0/50 [00:00<?, ?it/s]/tmp/ipykernel_80297/1903181417.py:19: RuntimeWarning: divide by zero encou
ntered in log
  noisyr = (np.log(noisyr) +20)/(20)
100%|██████████| 50/50 [00:00<00:00, 802.53it/s]
```

In [5]:

```
ntarget_a = np.asarray(ntarget)
print(ntarget_a.shape)

ntarget_a = np.reshape(ntarget_a, [50,80,109])

np.save("training_data_Noise.npy", ntarget_a, allow_pickle=True)
```

```
(50, 8720)
```

In [6]:

```
plt.imshow(ntarget_a[10,:,:])
```

Out[6]:

```
<matplotlib.image.AxesImage at 0x7fc18b361f40>
```

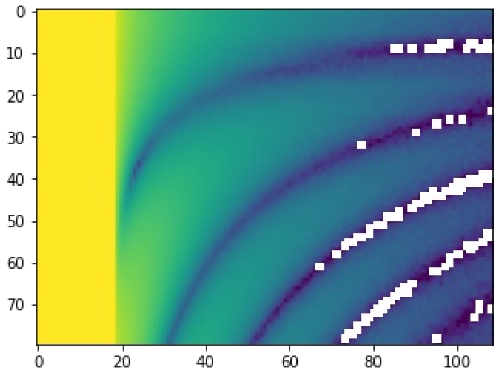

In [ ]:

Loading [MathJax]/jax/output/CommonHTML/fonts/TeX/fontdata.js

<!DOCTYPE html>

Implementation of the dropout into the dataset

Loading the normalized generated data and corresponding labels (normalized). Normalization of the data after the application of the dropout function. Saving the data.

In [1]:

```
import os
os.environ['TF_CPP_MIN_LOG_LEVEL'] = '1'
os.environ["CUDA_VISIBLE_DEVICES"]="0"

import tensorflow as tf
print("Num GPUs Available: ", len(tf.config.experimental.list_physical_devices('GPU')))
import keras
%matplotlib inline
```

```
Num GPUs Available:  1
```

In [2]:

```
import matplotlib.pylab as plt
import numpy as np
import pickle
from tqdm import tqdm
import random

from matplotlib import image
from matplotlib import pyplot
from matplotlib.pyplot import figure
```

In [ ]:

```
```

In [3]:

```
T = np.load("data_Norm.npy")

T = np.reshape(T, [50, 80*109])



Labels = np.load('Label_Norm.npy', allow_pickle=True)


label50=[]
for i in tqdm(range (1)):
    label50=np.append(label50,Labels)


label50 = np.reshape(label50, [50, 10])
print(label50.shape)


np.save("training_Label_Drop.npy", label50, allow_pickle=True)
```

```
100%|██████████| 1/1 [00:00<00:00, 3731.59it/s]
(50, 10)
```

In [4]:

```
target=T
#print(target)

from scipy.stats import poisson
from numpy import inf


#drop function
def drop(x,drp):
    layer = tf.keras.layers.Dropout(drp, input_shape=(2,))
    outputs = layer(x, training=True)
    outputs = outputs/(1/(1-drp)) #data normalization after dropout
    return outputs

#noisytarget reflectivity values
dtarget = []
numberofimages = 50
noisyr =[]
```

```python
for a in tqdm(range (numberofimages)):
    drp = random.uniform(0, 1)
    dropr = drop(target[a,:], drp)
    dtarget.append(dropr)
```

```
100%|██████████| 50/50 [00:00<00:00, 64.59it/s]
```

In [5]:
```python
dtarget_a = np.array(dtarget)
dtarget_a = np.reshape(dtarget_a, [numberofimages,80,109])

np.save("training_data_Drop.npy", dtarget_a, allow_pickle=True)
```

In [6]:
```python
plt.imshow(np.log(dtarget_a[10,:,:]))
```

```
/tmp/ipykernel_80267/2042752549.py:1: RuntimeWarning: divide by zero encountered in log
  plt.imshow(np.log(dtarget_a[10,:,:]))
```

Out[6]: <matplotlib.image.AxesImage at 0x7f04382a2400>

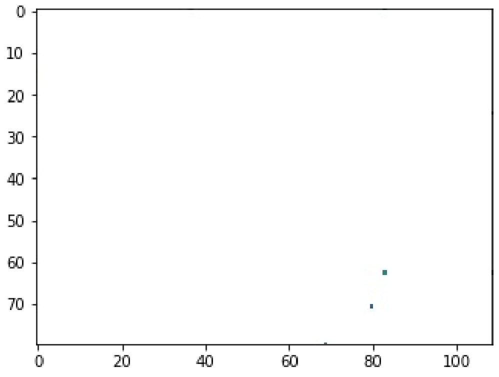

In [ ]:

Loading [MathJax]/jax/output/CommonHTML/fonts/TeX/fontdata.js

<!DOCTYPE html>

Retraining of the CNN on the noisy data

Here we load already normalized data with corresponding labels. We load the pre-trained CNN model and retrain it with the noisy data. We retrain the CNN for 20 epochs with adam optimizer and 20 epochs with adagrad optimizer.

To make the notebook simple, we use only a small amount of data (50 R(q,t)) to train the CNN. We load data from a "Training data" folder.

In [1]:

```
import os

os.environ['TF_CPP_MIN_LOG_LEVEL'] = '1'

os.environ["CUDA_VISIBLE_DEVICES"]="0"

import tensorflow as tf
import keras
gpus = tf.config.experimental.list_physical_devices("GPU")
tf.config.experimental.set_memory_growth(gpus[0], True)
%matplotlib inline
```

Retraining of the CNN with noisy data

In [2]:

```
import matplotlib.pylab as plt
import numpy as np
import pickle

from matplotlib import image
from matplotlib import pyplot
from matplotlib.pyplot import figure
```

In [3]:

```
T = np.load("training_data_Noise.npy", allow_pickle=True)
T_x = np.reshape(T, [50, 80, 109, 1])

Labels = np.load('training_Label_Noise.npy', allow_pickle=True)
Labels = tf.reshape(Labels, [50,10])
```

In [4]:

```
print(Labels[4,:])
```

```
tf.Tensor(
[0.85052572 0.32058857 0.74053516 0.90165227 0.69421226 0.46078153
 0.48397093 0.87050691 0.0686595  0.49003454], shape=(10,), dtype=float64)
```

In [5]:

```
#plt.plot(q_values,T_x[::,90,150])
#plt.show()
plt.imshow(T_x[3,:,:,:])
plt.ylabel('q_value')
plt.xlabel('curve number')
plt.show()
plt.plot(T_x[3,79,:,:])
plt.show()
```

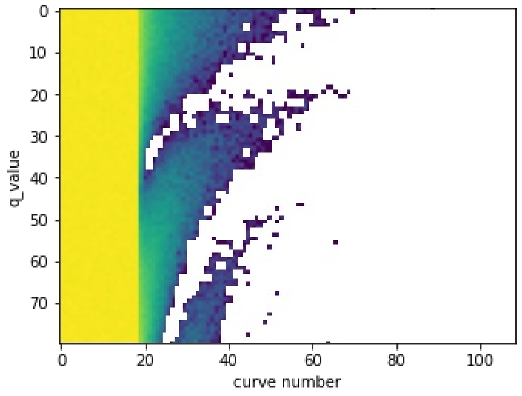

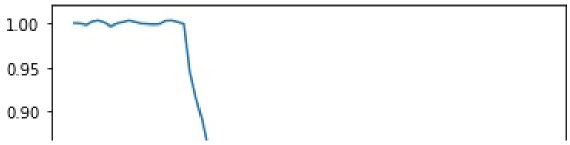

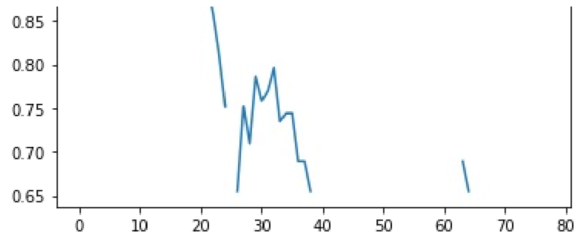

In [6]:
```python
from tensorflow import keras

from tensorflow.keras.models import Sequential
from keras.models import load_model
from keras.layers import Dense, Dropout, Activation, Dropout, Flatten, BatchNormalization
from keras import metrics
from keras.callbacks import ModelCheckpoint, Callback, ReduceLROnPlateau, CSVLogger
from keras import optimizers
from keras.layers.convolutional import Convolution2D, MaxPooling2D, ZeroPadding2D, Conv2D, AveragePooling2D
from keras.preprocessing.image import ImageDataGenerator
from tensorflow.keras.optimizers import Adam
from tensorflow.keras.callbacks import History
```

In [7]:
```python
def y_absolute_error(ind):
    def abs_err(y_true, y_pred):
        absolute_error = keras.backend.mean(abs(y_true[ind] - y_pred[ind]), axis=0)
        return absolute_error

    return abs_err
```

In [8]:
```python
model=load_model('conv_models/20211021_exp_adagrad')

model.summary()
```

```
Model: "sequential_1"
_________________________________________________________________
Layer (type)                 Output Shape              Param #
=================================================================
conv2d_12 (Conv2D)           (None, 79, 108, 32)       160
_________________________________________________________________
batch_normalization_12 (Batc (None, 79, 108, 32)       128
_________________________________________________________________
conv2d_13 (Conv2D)           (None, 78, 107, 32)       4128
_________________________________________________________________
batch_normalization_13 (Batc (None, 78, 107, 32)       128
_________________________________________________________________
conv2d_14 (Conv2D)           (None, 77, 106, 32)       4128
_________________________________________________________________
max_pooling2d_4 (MaxPooling2 (None, 38, 53, 32)        0
_________________________________________________________________
batch_normalization_14 (Batc (None, 38, 53, 32)        128
_________________________________________________________________
conv2d_15 (Conv2D)           (None, 37, 52, 64)        8256
_________________________________________________________________
batch_normalization_15 (Batc (None, 37, 52, 64)        256
_________________________________________________________________
conv2d_16 (Conv2D)           (None, 36, 51, 64)        16448
_________________________________________________________________
batch_normalization_16 (Batc (None, 36, 51, 64)        256
_________________________________________________________________
conv2d_17 (Conv2D)           (None, 35, 50, 64)        16448
_________________________________________________________________
max_pooling2d_5 (MaxPooling2 (None, 17, 25, 64)        0
_________________________________________________________________
batch_normalization_17 (Batc (None, 17, 25, 64)        256
_________________________________________________________________
conv2d_18 (Conv2D)           (None, 16, 24, 128)       32896
_________________________________________________________________
batch_normalization_18 (Batc (None, 16, 24, 128)       512
_________________________________________________________________
conv2d_19 (Conv2D)           (None, 15, 23, 128)       65664
_________________________________________________________________
batch_normalization_19 (Batc (None, 15, 23, 128)       512
_________________________________________________________________
conv2d_20 (Conv2D)           (None, 14, 22, 128)       65664
_________________________________________________________________
max_pooling2d_6 (MaxPooling2 (None, 7, 11, 128)        0
_________________________________________________________________
batch_normalization_20 (Batc (None, 7, 11, 128)        512
```

```
_________________________________________________________________
conv2d_21 (Conv2D)           (None, 6, 10, 256)        131328
_________________________________________________________________
batch_normalization_21 (Batc (None, 6, 10, 256)        1024
_________________________________________________________________
conv2d_22 (Conv2D)           (None, 5, 9, 256)         262400
_________________________________________________________________
batch_normalization_22 (Batc (None, 5, 9, 256)         1024
_________________________________________________________________
conv2d_23 (Conv2D)           (None, 4, 8, 256)         262400
_________________________________________________________________
max_pooling2d_7 (MaxPooling2 (None, 2, 4, 256)         0
_________________________________________________________________
batch_normalization_23 (Batc (None, 2, 4, 256)         1024
_________________________________________________________________
flatten_1 (Flatten)          (None, 2048)              0
_________________________________________________________________
dense_7 (Dense)              (None, 64)                131136
_________________________________________________________________
dense_8 (Dense)              (None, 32)                2080
_________________________________________________________________
dense_9 (Dense)              (None, 32)                1056
_________________________________________________________________
dense_10 (Dense)             (None, 16)                528
_________________________________________________________________
dense_11 (Dense)             (None, 16)                272
_________________________________________________________________
dense_12 (Dense)             (None, 10)                170
=================================================================
Total params: 1,010,922
Trainable params: 1,008,042
Non-trainable params: 2,880
_________________________________________________________________
```

In [ ]:

In [9]:

```python
model.compile(optimizer= 'adam',
              loss='mean_squared_error',
              metrics=['mean_absolute_error'])

model.fit(T_x, #X_training,
                    Labels, #thickness_training,
                    epochs=20,
                    batch_size=512,
                    verbose=1,
                    validation_split=0.2
                   )

score, acc = model.evaluate(T_x, Labels)

print('Test score:', score)
print('Test accuracy:', acc)
```

```
Epoch 1/20
2022-05-26 12:14:48.320058: W tensorflow/core/common_runtime/bfc_allocator.cc:248] Allocator (GPU_0_bfc) ran out of memory trying to allocate 373.02MiB with freed_by_count=0. The caller indicates that this is not a failure, but may mean that there could be performance gains if more memory were available.
2022-05-26 12:14:48.329993: W tensorflow/core/common_runtime/bfc_allocator.cc:248] Allocator (GPU_0_bfc) ran out of memory trying to allocate 373.02MiB with freed_by_count=0. The caller indicates that this is not a failure, but may mean that there could be performance gains if more memory were available.
2022-05-26 12:14:48.350446: W tensorflow/core/common_runtime/bfc_allocator.cc:248] Allocator (GPU_0_bfc) ran out of memory trying to allocate 373.02MiB with freed_by_count=0. The caller indicates that this is not a failure, but may mean that there could be performance gains if more memory were available.
2022-05-26 12:14:48.370932: W tensorflow/core/common_runtime/bfc_allocator.cc:248] Allocator (GPU_0_bfc) ran out of memory trying to allocate 373.02MiB with freed_by_count=0. The caller indicates that this is not a failure, but may mean that there could be performance gains if more memory were available.

1/1 [==============================] - ETA: 0s - loss: 0.0690 - mean_absolute_error: 0.2180

2022-05-26 12:14:48.602154: W tensorflow/core/common_runtime/bfc_allocator.cc:248] Allocator (GPU_0_bfc) ran out of memory trying to allocate 380.85MiB with freed_by_count=0. The caller indicates that this is not a failure, but may mean that there could be performance gains if more memory were available.
2022-05-26 12:14:48.644478: W tensorflow/core/common_runtime/bfc_allocator.cc:248] Allocator (GPU_0_bfc) ran out of memory trying to allocate 381.75MiB with freed_by_count=0. The caller indicates that this is not a failure, but may mean that there could be performance gains if more memory were available.
1/1 [==============================] - 4s 4s/step - loss: 0.0690 - mean_absolute_error: 0.2180 - val_loss: 0.0718 - val_mean_absolute_error: 0.2111
Epoch 2/20
```

```
1/1 [==============================] - 0s 107ms/step - loss: 0.0633 - mean_absolute_error: 0.2087 - val_loss: 0.0
684 - val_mean_absolute_error: 0.2060
Epoch 3/20
1/1 [==============================] - 0s 105ms/step - loss: 0.0602 - mean_absolute_error: 0.2002 - val_loss: 0.0
637 - val_mean_absolute_error: 0.1989
Epoch 4/20
1/1 [==============================] - 0s 105ms/step - loss: 0.0584 - mean_absolute_error: 0.1957 - val_loss: 0.0
596 - val_mean_absolute_error: 0.1943
Epoch 5/20
1/1 [==============================] - 0s 105ms/step - loss: 0.0571 - mean_absolute_error: 0.1928 - val_loss: 0.0
576 - val_mean_absolute_error: 0.1926
Epoch 6/20
1/1 [==============================] - 0s 104ms/step - loss: 0.0553 - mean_absolute_error: 0.1905 - val_loss: 0.0
634 - val_mean_absolute_error: 0.2026
Epoch 7/20
1/1 [==============================] - 0s 105ms/step - loss: 0.0521 - mean_absolute_error: 0.1862 - val_loss: 0.0
706 - val_mean_absolute_error: 0.2147
Epoch 8/20
1/1 [==============================] - 0s 104ms/step - loss: 0.0471 - mean_absolute_error: 0.1779 - val_loss: 0.0
689 - val_mean_absolute_error: 0.2163
Epoch 9/20
1/1 [==============================] - 0s 106ms/step - loss: 0.0427 - mean_absolute_error: 0.1707 - val_loss: 0.0
624 - val_mean_absolute_error: 0.2048
Epoch 10/20
1/1 [==============================] - 0s 105ms/step - loss: 0.0428 - mean_absolute_error: 0.1708 - val_loss: 0.0
613 - val_mean_absolute_error: 0.2004
Epoch 11/20
1/1 [==============================] - 0s 104ms/step - loss: 0.0418 - mean_absolute_error: 0.1683 - val_loss: 0.0
634 - val_mean_absolute_error: 0.2023
Epoch 12/20
1/1 [==============================] - 0s 109ms/step - loss: 0.0388 - mean_absolute_error: 0.1622 - val_loss: 0.0
679 - val_mean_absolute_error: 0.2086
Epoch 13/20
1/1 [==============================] - 0s 109ms/step - loss: 0.0379 - mean_absolute_error: 0.1607 - val_loss: 0.0
724 - val_mean_absolute_error: 0.2147
Epoch 14/20
1/1 [==============================] - 0s 107ms/step - loss: 0.0390 - mean_absolute_error: 0.1639 - val_loss: 0.0
752 - val_mean_absolute_error: 0.2192
Epoch 15/20
1/1 [==============================] - 0s 108ms/step - loss: 0.0393 - mean_absolute_error: 0.1645 - val_loss: 0.0
770 - val_mean_absolute_error: 0.2218
Epoch 16/20
1/1 [==============================] - 0s 109ms/step - loss: 0.0384 - mean_absolute_error: 0.1619 - val_loss: 0.0
784 - val_mean_absolute_error: 0.2228
Epoch 17/20
1/1 [==============================] - 0s 105ms/step - loss: 0.0368 - mean_absolute_error: 0.1580 - val_loss: 0.0
791 - val_mean_absolute_error: 0.2231
Epoch 18/20
1/1 [==============================] - 0s 108ms/step - loss: 0.0355 - mean_absolute_error: 0.1557 - val_loss: 0.0
792 - val_mean_absolute_error: 0.2229
Epoch 19/20
1/1 [==============================] - 0s 106ms/step - loss: 0.0354 - mean_absolute_error: 0.1563 - val_loss: 0.0
795 - val_mean_absolute_error: 0.2229
Epoch 20/20
1/1 [==============================] - 0s 109ms/step - loss: 0.0362 - mean_absolute_error: 0.1582 - val_loss: 0.0
793 - val_mean_absolute_error: 0.2225
1/2 [==============>...............] - ETA: 0s - loss: 0.0725 - mean_absolute_error: 0.2170
```

```
2022-05-26 12:14:52.084944: W tensorflow/core/common_runtime/bfc_allocator.cc:314] Garbage collection: deallocate
free memory regions (i.e., allocations) so that we can re-allocate a larger region to avoid OOM due to memory fra
gmentation. If you see this message frequently, you are running near the threshold of the available device memory
and re-allocation may incur great performance overhead. You may try smaller batch sizes to observe the performanc
e impact. Set TF_ENABLE_GPU_GARBAGE_COLLECTION=false if you'd like to disable this feature.
```

```
2/2 [==============================] - 1s 198ms/step - loss: 0.0736 - mean_absolute_error: 0.2162
Test score: 0.07363776117563248
Test accuracy: 0.21619747579097748
```

In [10]:

```
model.compile(optimizer= 'adagrad',
              loss='mean_squared_error',
              metrics=['mean_absolute_error'])

model.fit(T_x, #X_training,
        Labels, #thickness_training,
        epochs=20,
        batch_size=512,
        verbose=1,
```

```
        validation_split=0.2
        #validation_data=(X_val, thickness_val),
        )

score, acc = model.evaluate(T_x, Labels)

print('Test score:', score)
print('Test accuracy:', acc)
```

```
Epoch 1/20
1/1 [==============================] - 2s 2s/step - loss: 0.0369 - mean_absolute_error: 0.1596 - val_loss: 0.0791
- val_mean_absolute_error: 0.2223
Epoch 2/20
1/1 [==============================] - 0s 106ms/step - loss: 0.0369 - mean_absolute_error: 0.1595 - val_loss: 0.0
806 - val_mean_absolute_error: 0.2234
Epoch 3/20
1/1 [==============================] - 0s 105ms/step - loss: 0.0369 - mean_absolute_error: 0.1595 - val_loss: 0.0
800 - val_mean_absolute_error: 0.2234
Epoch 4/20
1/1 [==============================] - 0s 105ms/step - loss: 0.0368 - mean_absolute_error: 0.1594 - val_loss: 0.0
795 - val_mean_absolute_error: 0.2231
Epoch 5/20
1/1 [==============================] - 0s 106ms/step - loss: 0.0368 - mean_absolute_error: 0.1593 - val_loss: 0.0
788 - val_mean_absolute_error: 0.2229
Epoch 6/20
1/1 [==============================] - 0s 105ms/step - loss: 0.0368 - mean_absolute_error: 0.1593 - val_loss: 0.0
776 - val_mean_absolute_error: 0.2219
Epoch 7/20
1/1 [==============================] - 0s 105ms/step - loss: 0.0367 - mean_absolute_error: 0.1592 - val_loss: 0.0
755 - val_mean_absolute_error: 0.2200
Epoch 8/20
1/1 [==============================] - 0s 105ms/step - loss: 0.0367 - mean_absolute_error: 0.1592 - val_loss: 0.0
735 - val_mean_absolute_error: 0.2181
Epoch 9/20
1/1 [==============================] - 0s 106ms/step - loss: 0.0367 - mean_absolute_error: 0.1591 - val_loss: 0.0
706 - val_mean_absolute_error: 0.2152
Epoch 10/20
1/1 [==============================] - 0s 103ms/step - loss: 0.0367 - mean_absolute_error: 0.1590 - val_loss: 0.0
679 - val_mean_absolute_error: 0.2130
Epoch 11/20
1/1 [==============================] - 0s 106ms/step - loss: 0.0366 - mean_absolute_error: 0.1590 - val_loss: 0.0
660 - val_mean_absolute_error: 0.2104
Epoch 12/20
1/1 [==============================] - 0s 105ms/step - loss: 0.0366 - mean_absolute_error: 0.1589 - val_loss: 0.0
636 - val_mean_absolute_error: 0.2069
Epoch 13/20
1/1 [==============================] - 0s 103ms/step - loss: 0.0366 - mean_absolute_error: 0.1589 - val_loss: 0.0
619 - val_mean_absolute_error: 0.2040
Epoch 14/20
1/1 [==============================] - 0s 104ms/step - loss: 0.0366 - mean_absolute_error: 0.1588 - val_loss: 0.0
596 - val_mean_absolute_error: 0.2009
Epoch 15/20
1/1 [==============================] - 0s 104ms/step - loss: 0.0365 - mean_absolute_error: 0.1588 - val_loss: 0.0
580 - val_mean_absolute_error: 0.1983
Epoch 16/20
1/1 [==============================] - 0s 108ms/step - loss: 0.0365 - mean_absolute_error: 0.1587 - val_loss: 0.0
565 - val_mean_absolute_error: 0.1962
Epoch 17/20
1/1 [==============================] - 0s 108ms/step - loss: 0.0365 - mean_absolute_error: 0.1586 - val_loss: 0.0
551 - val_mean_absolute_error: 0.1938
Epoch 18/20
1/1 [==============================] - 0s 115ms/step - loss: 0.0365 - mean_absolute_error: 0.1586 - val_loss: 0.0
538 - val_mean_absolute_error: 0.1913
Epoch 19/20
1/1 [==============================] - 0s 107ms/step - loss: 0.0364 - mean_absolute_error: 0.1585 - val_loss: 0.0
527 - val_mean_absolute_error: 0.1890
Epoch 20/20
1/1 [==============================] - 0s 103ms/step - loss: 0.0364 - mean_absolute_error: 0.1585 - val_loss: 0.0
514 - val_mean_absolute_error: 0.1859
2/2 [==============================] - 0s 14ms/step - loss: 0.0555 - mean_absolute_error: 0.1944
Test score: 0.055526405572891235
Test accuracy: 0.1944005936384201
```

In [11]:

```
#model.save('convolutionalmodels/Noise_adagrad')
```

Loading [MathJax]/jax/output/CommonHTML/fonts/TeX/fontdata.js

<!DOCTYPE html>

Retraining of the CNN on the dropout data

Loading the sparsely sampled data and corresponding labels. Normalization of the data and reshaping them to be suitable for the CNN training.

Later we load the trained CNN model on the full data set and retrain the model with the sparsely sampled data. We retrain CNN with adam optimiser for 20 epochs and with adagrad for next 20 epochs.

In [1]:

```
import os

os.environ['TF_CPP_MIN_LOG_LEVEL'] = '1'

os.environ["CUDA_VISIBLE_DEVICES"]="1"

import tensorflow as tf
import keras
gpus = tf.config.experimental.list_physical_devices("GPU")
tf.config.experimental.set_memory_growth(gpus[0], True)

%matplotlib inline
```

Retraining of the CNN with noisy data

In [2]:

```
import matplotlib.pylab as plt
import numpy as np
import pickle

from matplotlib import image
from matplotlib import pyplot
from matplotlib.pyplot import figure
```

In [3]:

```
T = np.load("training_data_Drop.npy")
T_x = np.reshape(T, [50, 80, 109, 1])


Labels = np.load('training_Label_Drop.npy', allow_pickle=True)
Labels_a = tf.reshape(Labels, [50,10])

print(T_x.shape)
print(Labels.shape)
```

```
(50, 80, 109, 1)
(50, 10)
```

In [4]:

```
print(Labels[4,:])
```

```
[0.85052572 0.32058857 0.74053516 0.90165227 0.69421226 0.46078153
 0.48397093 0.87050691 0.0686595  0.49003454]
```

In [5]:

```
plt.imshow(T[3,:,:])
plt.ylabel('q_value')
plt.xlabel('curve number')
plt.show()
plt.plot(T[38,79,:])
plt.show()
```

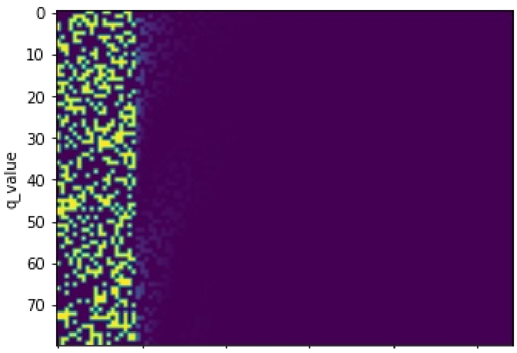

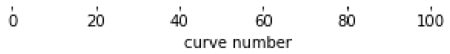

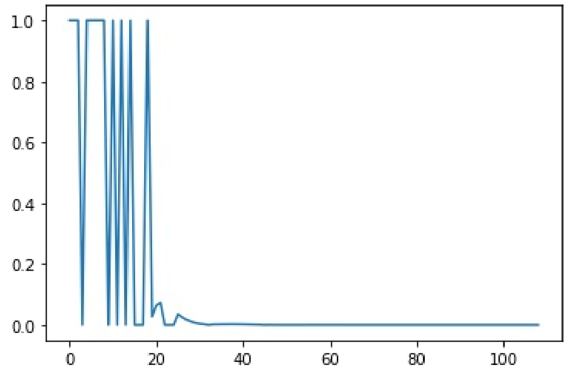

In [6]:

```
from tensorflow import keras

from tensorflow.keras.models import Sequential
from keras.models import load_model
from keras.layers import Dense, Dropout, Activation, Dropout, Flatten, BatchNormalization
from keras import metrics
from keras.callbacks import ModelCheckpoint, Callback, ReduceLROnPlateau, CSVLogger
from keras import optimizers
from keras.layers.convolutional import Convolution2D, MaxPooling2D, ZeroPadding2D, Conv2D, AveragePooling2D
from keras.preprocessing.image import ImageDataGenerator
from tensorflow.keras.optimizers import Adam
from tensorflow.keras.callbacks import History
```

In [7]:

```
def y_absolute_error(ind):
    def abs_err(y_true, y_pred):
        absolute_error = keras.backend.mean(abs(y_true[ind] - y_pred[ind]), axis=0)
        return absolute_error

    return abs_err
```

In [8]:

```
model=load_model('conv_models/20211021_exp_adagrad')

model.summary()
```

```
Model: "sequential_1"
_________________________________________________________________
Layer (type)                 Output Shape              Param #
=================================================================
conv2d_12 (Conv2D)           (None, 79, 108, 32)       160
_________________________________________________________________
batch_normalization_12 (Batc (None, 79, 108, 32)       128
_________________________________________________________________
conv2d_13 (Conv2D)           (None, 78, 107, 32)       4128
_________________________________________________________________
batch_normalization_13 (Batc (None, 78, 107, 32)       128
_________________________________________________________________
conv2d_14 (Conv2D)           (None, 77, 106, 32)       4128
_________________________________________________________________
max_pooling2d_4 (MaxPooling2 (None, 38, 53, 32)        0
_________________________________________________________________
batch_normalization_14 (Batc (None, 38, 53, 32)        128
_________________________________________________________________
conv2d_15 (Conv2D)           (None, 37, 52, 64)        8256
_________________________________________________________________
batch_normalization_15 (Batc (None, 37, 52, 64)        256
_________________________________________________________________
conv2d_16 (Conv2D)           (None, 36, 51, 64)        16448
_________________________________________________________________
batch_normalization_16 (Batc (None, 36, 51, 64)        256
_________________________________________________________________
conv2d_17 (Conv2D)           (None, 35, 50, 64)        16448
_________________________________________________________________
max_pooling2d_5 (MaxPooling2 (None, 17, 25, 64)        0
_________________________________________________________________
batch_normalization_17 (Batc (None, 17, 25, 64)        256
_________________________________________________________________
conv2d_18 (Conv2D)           (None, 16, 24, 128)       32896
_________________________________________________________________
batch_normalization_18 (Batc (None, 16, 24, 128)       512
_________________________________________________________________
conv2d_19 (Conv2D)           (None, 15, 23, 128)       65664
```

```
batch_normalization_19 (Batc (None, 15, 23, 128)       512
_________________________________________________________________
conv2d_20 (Conv2D)           (None, 14, 22, 128)       65664
_________________________________________________________________
max_pooling2d_6 (MaxPooling2 (None, 7, 11, 128)        0
_________________________________________________________________
batch_normalization_20 (Batc (None, 7, 11, 128)        512
_________________________________________________________________
conv2d_21 (Conv2D)           (None, 6, 10, 256)        131328
_________________________________________________________________
batch_normalization_21 (Batc (None, 6, 10, 256)        1024
_________________________________________________________________
conv2d_22 (Conv2D)           (None, 5, 9, 256)         262400
_________________________________________________________________
batch_normalization_22 (Batc (None, 5, 9, 256)         1024
_________________________________________________________________
conv2d_23 (Conv2D)           (None, 4, 8, 256)         262400
_________________________________________________________________
max_pooling2d_7 (MaxPooling2 (None, 2, 4, 256)         0
_________________________________________________________________
batch_normalization_23 (Batc (None, 2, 4, 256)         1024
_________________________________________________________________
flatten_1 (Flatten)          (None, 2048)              0
_________________________________________________________________
dense_7 (Dense)              (None, 64)                131136
_________________________________________________________________
dense_8 (Dense)              (None, 32)                2080
_________________________________________________________________
dense_9 (Dense)              (None, 32)                1056
_________________________________________________________________
dense_10 (Dense)             (None, 16)                528
_________________________________________________________________
dense_11 (Dense)             (None, 16)                272
_________________________________________________________________
dense_12 (Dense)             (None, 10)                170
=================================================================
Total params: 1,010,922
Trainable params: 1,008,042
Non-trainable params: 2,880
_________________________________________________________________
```

In [9]:

```
opt = keras.optimizers.Adam(learning_rate=0.01, decay=1e-6)


model.compile(optimizer= 'adam',
              loss='mean_squared_error',
              metrics=['mean_absolute_error'])

model.fit(T_x, #X_training,
                    Labels, #thickness_training,
                    epochs=20,
                    batch_size=512,
                    verbose=1,
                    validation_split=0.2
                   )

score, acc = model.evaluate(T_x, Labels)

print('Test score:', score)
print('Test accuracy:', acc)
```

```
Epoch 1/20
1/1 [==============================] - 4s 4s/step - loss: 0.0875 - mean_absolute_error: 0.2383 - val_loss: 0.0734
- val_mean_absolute_error: 0.2053
Epoch 2/20
1/1 [==============================] - 0s 110ms/step - loss: 0.0713 - mean_absolute_error: 0.2135 - val_loss: 0.0
723 - val_mean_absolute_error: 0.2037
Epoch 3/20
1/1 [==============================] - 0s 103ms/step - loss: 0.0594 - mean_absolute_error: 0.1886 - val_loss: 0.0
725 - val_mean_absolute_error: 0.2052
Epoch 4/20
1/1 [==============================] - 0s 103ms/step - loss: 0.0519 - mean_absolute_error: 0.1750 - val_loss: 0.0
693 - val_mean_absolute_error: 0.2025
Epoch 5/20
1/1 [==============================] - 0s 103ms/step - loss: 0.0438 - mean_absolute_error: 0.1575 - val_loss: 0.0
688 - val_mean_absolute_error: 0.2049
Epoch 6/20
1/1 [==============================] - 0s 102ms/step - loss: 0.0385 - mean_absolute_error: 0.1465 - val_loss: 0.0
702 - val_mean_absolute_error: 0.2077
Epoch 7/20
1/1 [==============================] - 0s 102ms/step - loss: 0.0330 - mean_absolute_error: 0.1330 - val_loss: 0.0
```

```
723 - val_mean_absolute_error: 0.2147
Epoch 8/20
1/1 [==============================] - 0s 102ms/step - loss: 0.0287 - mean_absolute_error: 0.1223 - val_loss: 0.0
742 - val_mean_absolute_error: 0.2192
Epoch 9/20
1/1 [==============================] - 0s 103ms/step - loss: 0.0257 - mean_absolute_error: 0.1145 - val_loss: 0.0
766 - val_mean_absolute_error: 0.2237
Epoch 10/20
1/1 [==============================] - 0s 103ms/step - loss: 0.0231 - mean_absolute_error: 0.1062 - val_loss: 0.0
798 - val_mean_absolute_error: 0.2279
Epoch 11/20
1/1 [==============================] - 0s 102ms/step - loss: 0.0210 - mean_absolute_error: 0.0987 - val_loss: 0.0
819 - val_mean_absolute_error: 0.2307
Epoch 12/20
1/1 [==============================] - 0s 102ms/step - loss: 0.0196 - mean_absolute_error: 0.0948 - val_loss: 0.0
824 - val_mean_absolute_error: 0.2317
Epoch 13/20
1/1 [==============================] - 0s 103ms/step - loss: 0.0179 - mean_absolute_error: 0.0890 - val_loss: 0.0
824 - val_mean_absolute_error: 0.2326
Epoch 14/20
1/1 [==============================] - 0s 102ms/step - loss: 0.0158 - mean_absolute_error: 0.0831 - val_loss: 0.0
825 - val_mean_absolute_error: 0.2334
Epoch 15/20
1/1 [==============================] - 0s 109ms/step - loss: 0.0139 - mean_absolute_error: 0.0770 - val_loss: 0.0
827 - val_mean_absolute_error: 0.2341
Epoch 16/20
1/1 [==============================] - 0s 102ms/step - loss: 0.0124 - mean_absolute_error: 0.0730 - val_loss: 0.0
833 - val_mean_absolute_error: 0.2349
Epoch 17/20
1/1 [==============================] - 0s 103ms/step - loss: 0.0112 - mean_absolute_error: 0.0685 - val_loss: 0.0
835 - val_mean_absolute_error: 0.2352
Epoch 18/20
1/1 [==============================] - 0s 103ms/step - loss: 0.0102 - mean_absolute_error: 0.0623 - val_loss: 0.0
833 - val_mean_absolute_error: 0.2350
Epoch 19/20
1/1 [==============================] - 0s 103ms/step - loss: 0.0095 - mean_absolute_error: 0.0587 - val_loss: 0.0
833 - val_mean_absolute_error: 0.2348
Epoch 20/20
1/1 [==============================] - 0s 103ms/step - loss: 0.0087 - mean_absolute_error: 0.0547 - val_loss: 0.0
832 - val_mean_absolute_error: 0.2344
2/2 [==============================] - 0s 191ms/step - loss: 0.0885 - mean_absolute_error: 0.2449
Test score: 0.08847814798355103
Test accuracy: 0.24487636983394623
```

In [10]:

```python
opt = keras.optimizers.Adam(learning_rate=0.01, decay=1e-6)
#opt = keras.optimizers.SGD

model.compile(optimizer= 'adagrad',
              loss='mean_squared_error',
              metrics=['mean_absolute_error'])

model.fit(T_x, #X_training,
        Labels, #thickness_training,
        epochs=20,
        batch_size=512,
        verbose=1,
        validation_split=0.2
        #validation_data=(X_val, thickness_val),
        )

score, acc = model.evaluate(T_x, Labels)

print('Test score:', score)
print('Test accuracy:', acc)
```

```
Epoch 1/20
1/1 [==============================] - 2s 2s/step - loss: 0.0078 - mean_absolute_error: 0.0512 - val_loss: 0.0832
- val_mean_absolute_error: 0.2342
Epoch 2/20
1/1 [==============================] - 0s 104ms/step - loss: 0.0078 - mean_absolute_error: 0.0511 - val_loss: 0.0
832 - val_mean_absolute_error: 0.2342
Epoch 3/20
1/1 [==============================] - 0s 102ms/step - loss: 0.0078 - mean_absolute_error: 0.0511 - val_loss: 0.0
832 - val_mean_absolute_error: 0.2341
Epoch 4/20
1/1 [==============================] - 0s 102ms/step - loss: 0.0078 - mean_absolute_error: 0.0511 - val_loss: 0.0
830 - val_mean_absolute_error: 0.2341
Epoch 5/20
1/1 [==============================] - 0s 103ms/step - loss: 0.0078 - mean_absolute_error: 0.0511 - val_loss: 0.0
828 - val_mean_absolute_error: 0.2341
Epoch 6/20
1/1 [==============================] - 0s 102ms/step - loss: 0.0078 - mean_absolute_error: 0.0511 - val_loss: 0.0
```

```
824 - val_mean_absolute_error: 0.2340
Epoch 7/20
1/1 [==============================] - 0s 101ms/step - loss: 0.0078 - mean_absolute_error: 0.0511 - val_loss: 0.0
820 - val_mean_absolute_error: 0.2335
Epoch 8/20
1/1 [==============================] - 0s 101ms/step - loss: 0.0078 - mean_absolute_error: 0.0511 - val_loss: 0.0
816 - val_mean_absolute_error: 0.2330
Epoch 9/20
1/1 [==============================] - 0s 101ms/step - loss: 0.0078 - mean_absolute_error: 0.0511 - val_loss: 0.0
812 - val_mean_absolute_error: 0.2324
Epoch 10/20
1/1 [==============================] - 0s 100ms/step - loss: 0.0077 - mean_absolute_error: 0.0511 - val_loss: 0.0
810 - val_mean_absolute_error: 0.2320
Epoch 11/20
1/1 [==============================] - 0s 100ms/step - loss: 0.0077 - mean_absolute_error: 0.0510 - val_loss: 0.0
807 - val_mean_absolute_error: 0.2315
Epoch 12/20
1/1 [==============================] - 0s 100ms/step - loss: 0.0077 - mean_absolute_error: 0.0510 - val_loss: 0.0
804 - val_mean_absolute_error: 0.2308
Epoch 13/20
1/1 [==============================] - 0s 100ms/step - loss: 0.0077 - mean_absolute_error: 0.0510 - val_loss: 0.0
800 - val_mean_absolute_error: 0.2298
Epoch 14/20
1/1 [==============================] - 0s 100ms/step - loss: 0.0077 - mean_absolute_error: 0.0510 - val_loss: 0.0
797 - val_mean_absolute_error: 0.2286
Epoch 15/20
1/1 [==============================] - 0s 100ms/step - loss: 0.0077 - mean_absolute_error: 0.0510 - val_loss: 0.0
799 - val_mean_absolute_error: 0.2271
Epoch 16/20
1/1 [==============================] - 0s 101ms/step - loss: 0.0077 - mean_absolute_error: 0.0510 - val_loss: 0.0
805 - val_mean_absolute_error: 0.2265
Epoch 17/20
1/1 [==============================] - 0s 100ms/step - loss: 0.0077 - mean_absolute_error: 0.0510 - val_loss: 0.0
814 - val_mean_absolute_error: 0.2261
Epoch 18/20
1/1 [==============================] - 0s 100ms/step - loss: 0.0077 - mean_absolute_error: 0.0510 - val_loss: 0.0
823 - val_mean_absolute_error: 0.2258
Epoch 19/20
1/1 [==============================] - 0s 101ms/step - loss: 0.0077 - mean_absolute_error: 0.0509 - val_loss: 0.0
829 - val_mean_absolute_error: 0.2260
Epoch 20/20
1/1 [==============================] - 0s 101ms/step - loss: 0.0077 - mean_absolute_error: 0.0509 - val_loss: 0.0
833 - val_mean_absolute_error: 0.2264
2/2 [==============================] - 0s 13ms/step - loss: 0.0955 - mean_absolute_error: 0.2467
Test score: 0.09546801447868347
Test accuracy: 0.24669699370861053
```

In [11]:

```
#model.save('convolutionalmodels/20220303_Noise_adagrad10')
```

Loading [MathJax]/jax/output/CommonHTML/fonts/TeX/fontdata.js

<!DOCTYPE html>

Training of the CNN on the synthetic data

Here we load the data and corresponding labels. We normalize the data and reshape them for the CNN training. Later we create the CNN architecture and train the CNN with the synthetic data. At the end of the notebook, we plot parity plots for all the parameters.

To make the notebook simple, we are using only a small amount of data (50 R(q,t)) to train the CNN. WE load data from a "Training data" folder.

In [1]:
```
import os

os.environ['TF_CPP_MIN_LOG_LEVEL'] = '1'

os.environ["CUDA_VISIBLE_DEVICES"]="1"

import tensorflow as tf
import keras
%matplotlib inline
```

In [2]:
```
#!pip install sklearn
#import sklearn
#from sklearn import preprocessing
```

In [3]:
```
import matplotlib.pylab as plt
import numpy as np
import pickle

from matplotlib import image
from matplotlib import pyplot
from matplotlib.pyplot import figure
```

In [4]:
```
T_data0 = np.load('Training data/training_data.npy', allow_pickle=True)


print(T_data0.shape)


T_data = T_data0
q_values = np.linspace(0.01, 0.14, 109)
#cycle 100s
print(T_data.shape)
print(q_values.shape)
```

```
(109, 4000)
(109, 4000)
(109,)
```

In [5]:
```
##data normalization
Training_data = (np.log(T_data)+20)/(20)
print((Training_data[:,5]))
plt.plot(Training_data[:,0:80])
plt.show()
T = np.transpose(Training_data)
print(T.shape)

T_x = np.reshape(T, [50, 80, 109, 1])

print(T.shape)
print(T_x.shape)

plt.imshow(T_x[42,:,:,:])
plt.show()
```

```
[1.         1.         1.         1.         1.         1.
 1.         1.         1.         1.         1.         1.
 1.         1.         1.         1.         1.         1.
 1.         0.94516205 0.9219699  0.90438654 0.88969116 0.87684117
 0.86530353 0.85476173 0.84500937 0.83590288 0.82733751 0.81923399
 0.81153063 0.80417822 0.79713676 0.79037323 0.78385993 0.77757341
 0.77149355 0.76560297 0.75988651 0.75433086 0.74892426 0.74365624
 0.73851745 0.73349951 0.72859483 0.72379657 0.71909847 0.71449486
 0.70998053 0.70555071 0.701201   0.69692734 0.692726   0.6885935
 0.68452662 0.68052235 0.6765779  0.67269067 0.66885822 0.66507827
```

```
0.66134868 0.65766744 0.65403266 0.65044258 0.64689553 0.64338993
0.6399243  0.63649724 0.63310743 0.62975361 0.62643461 0.62314931
0.61989664 0.61667562 0.61348528 0.61032472 0.6071931  0.60408961
0.60101348 0.59796397 0.59494041 0.59194214 0.58896853 0.586019
0.58309299 0.58018998 0.57730945 0.57445094 0.571614   0.5687982
0.56600314 0.56322843 0.56047372 0.55773867 0.55502294 0.55232625
0.54964831 0.54698884 0.54434758 0.54172431 0.53911879 0.53653082
0.53396019 0.53140672 0.52887023 0.52635056 0.52384755 0.52136106
0.51889096]
```

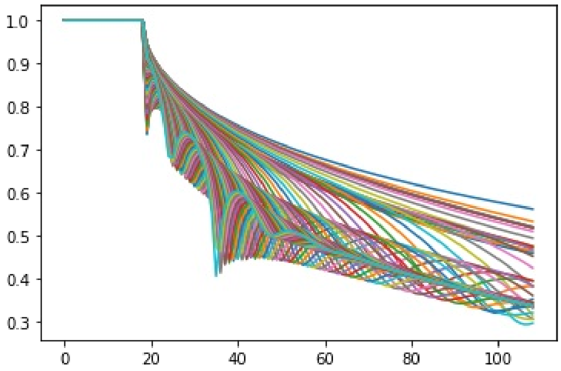


```
(4000, 109)
(4000, 109)
(50, 80, 109, 1)
```

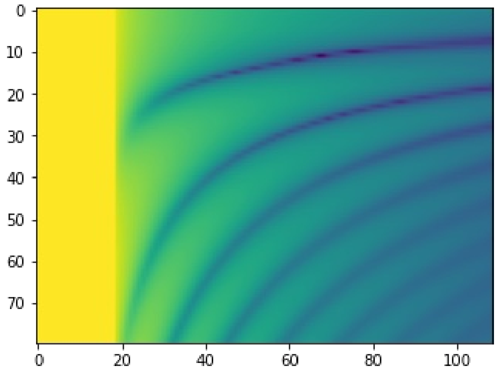

In [6]:

```
Label0 = np.load('Training data/label_Theta.npy', allow_pickle=True)

print(Label0.shape)

Label = Label0
Labels = Label[:,:]
print(Labels.shape)

#Labels normalization
Training_y = np.zeros([10,0])
print(Labels[9,:])
#G1
Labels[:,0] = Labels[:,0]-0.1
print("G1:",np.min(Labels[:,0]),np.max(Labels[:,0]))

#G2
Labels[:,1] = Labels[:,1] - 0.2
print("G2:",min(Labels[:,1]),max(Labels[:,1]))

#G3
Labels[:,2] = Labels[:,2] - 0.2
print("G3:",min(Labels[:,2]),max(Labels[:,2]))

#G4
Labels[:,3] = Labels[:,3]*0.55
print("G4:",min(Labels[:,3]),max(Labels[:,3]))

#a
Labels[:,4] = Labels[:,4]*1.5
print("a:",min(Labels[:,4]),max(Labels[:,4]))

#b
Labels[:,5] = (Labels[:,5]+0.5)/1.3
print("b(exp decay):",min(Labels[:,5]),max(Labels[:,5]))

#c
Labels[:,6] = (Labels[:,6])*2
```

```
print("c:",min(Labels[:,6]),max(Labels[:,6]))

#d
Labels[:,7] = (Labels[:,7]+7.1)/7.5
print("d:",min(Labels[:,7]),max(Labels[:,7]))

#g
Labels[:,8] = Labels[:,8]*9
print("g:",min(Labels[:,8]),max(Labels[:,8]))

#sld
Labels[:,9] = Labels[:,9]/2e15
print("SLD:",min(Labels[:,9]),max(Labels[:,9]))

Labels_a = tf.reshape(Labels, [50,10])

print(Labels_a[9,:])

print("min_max of decay is:",min(Labels[:,2]),max(Labels[:,2]))
```

```
(50, 10)
(50, 10)
[ 9.15360795e-01  8.39129188e-01  8.15546955e-01  1.44073271e+00
  4.25670624e-01  5.68451358e-01  1.93828512e-01 -2.37865293e+00
  1.78094852e-02  8.89207762e+14]
G1: 0.42357182316869624 0.9960411751931698
G2: 0.31264580674623327 0.8533223084253214
G3: 0.5096286388614333 0.9976748566639384
G4: 0.496099077124988 0.9320850041371723
a: 0.32701074143295916 0.8594457283024506
b(exp decay): 0.4315089848754137 0.9044266967062095
c: 0.24484823409016288 0.7879069598344122
d: 0.038542643202681505 0.9221917086338601
g: 0.04661166900112835 0.924217775161194
SLD: 0.43284518659872806 0.9197085415661308
tf.Tensor(
[0.8153608  0.63912919 0.61554696 0.79240299 0.63850594 0.82188566
 0.38765702 0.62951294 0.16028537 0.44460388], shape=(10,), dtype=float64)
min_max of decay is: 0.5096286388614333 0.9976748566639384
```

In [7]:

```
plt.imshow(T_x[10,:,:,0])
plt.ylabel('q_value')
plt.xlabel('curve number')
plt.show()
plt.plot(T_x[30,79,:,0])
plt.show()
```

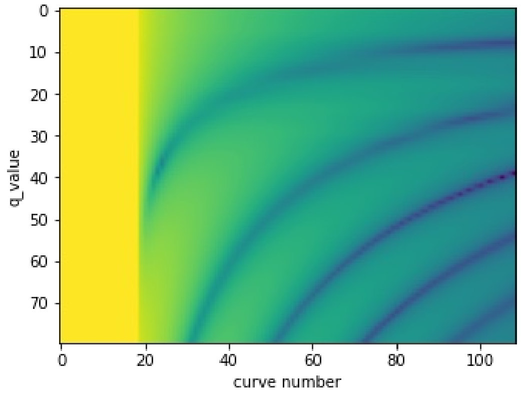

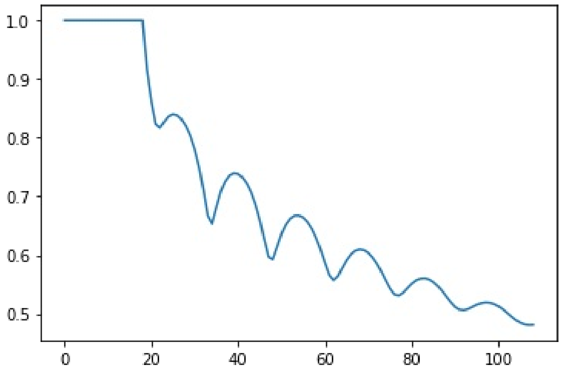

In [8]:

```
import tensorflow as tf
```

```
import tensorflow as tf
from tensorflow import keras
from tensorflow.keras.models import Sequential
from keras.models import load_model
from keras.layers import Dense, Dropout, Activation, Dropout, Flatten, BatchNormalization
from keras import metrics
from keras.callbacks import ModelCheckpoint, Callback, ReduceLROnPlateau, CSVLogger
from keras import optimizers
from keras.layers.convolutional import Convolution2D, MaxPooling2D, ZeroPadding2D, Conv2D, AveragePooling2D
from keras.preprocessing.image import ImageDataGenerator
from tensorflow.keras.optimizers import Adam
from tensorflow.keras.callbacks import History
```

In [9]:

```
def y_absolute_error(ind):
    def abs_err(y_true, y_pred):
        absolute_error = keras.backend.mean(abs(y_true[ind] - y_pred[ind]), axis=0)
        return absolute_error

    return abs_err
```

In [10]:

```
model = Sequential()

model.add(tf.keras.layers.Conv2D(32, (2, 2),activation="relu", input_shape = [80,109,1]))
model.add(tf.keras.layers.BatchNormalization())
model.add(tf.keras.layers.Conv2D(32, (2, 2), activation="relu"))
model.add(tf.keras.layers.MaxPooling2D((2, 2)))
model.add(tf.keras.layers.BatchNormalization())

model.add(tf.keras.layers.Conv2D(64, (2, 2), activation="relu"))
model.add(tf.keras.layers.BatchNormalization())
model.add(tf.keras.layers.Conv2D(64, (2, 2), activation="relu"))
model.add(tf.keras.layers.BatchNormalization())
model.add(tf.keras.layers.Conv2D(64, (2, 2), activation="relu"))
model.add(tf.keras.layers.MaxPooling2D((2, 2)))
model.add(tf.keras.layers.BatchNormalization())

model.add(tf.keras.layers.Conv2D(128, (2, 2), activation="relu"))
model.add(tf.keras.layers.BatchNormalization())
model.add(tf.keras.layers.Conv2D(128, (2, 2), activation="relu"))
model.add(tf.keras.layers.BatchNormalization())
model.add(tf.keras.layers.Conv2D(128, (2, 2), activation="relu"))
model.add(tf.keras.layers.MaxPooling2D((2, 2)))
model.add(tf.keras.layers.BatchNormalization())

model.add(tf.keras.layers.Conv2D(256, (2, 2), activation="relu"))
model.add(tf.keras.layers.BatchNormalization())
model.add(tf.keras.layers.Conv2D(256, (2, 2), activation="relu"))
model.add(tf.keras.layers.MaxPooling2D((2, 2)))

model.add(tf.keras.layers.Flatten())
model.add(tf.keras.layers.Dense(64, activation="relu"))
model.add(tf.keras.layers.Dense(32, activation="relu"))
model.add(tf.keras.layers.Dense(32, activation="relu"))
model.add(tf.keras.layers.Dense(16, activation="relu"))
model.add(tf.keras.layers.Dense(16, activation="relu"))
model.add(tf.keras.layers.Dense(10, activation="relu"))

model.summary()
```

```
Model: "sequential"
_________________________________________________________________
Layer (type)                 Output Shape              Param #
=================================================================
conv2d (Conv2D)              (None, 79, 108, 32)       160
_________________________________________________________________
batch_normalization (BatchNo (None, 79, 108, 32)       128
_________________________________________________________________
conv2d_1 (Conv2D)            (None, 78, 107, 32)       4128
_________________________________________________________________
max_pooling2d (MaxPooling2D) (None, 39, 53, 32)        0
_________________________________________________________________
batch_normalization_1 (Batch (None, 39, 53, 32)        128
_________________________________________________________________
conv2d_2 (Conv2D)            (None, 38, 52, 64)        8256
_________________________________________________________________
batch_normalization_2 (Batch (None, 38, 52, 64)        256
_________________________________________________________________
conv2d_3 (Conv2D)            (None, 37, 51, 64)        16448
_________________________________________________________________
batch_normalization_3 (Batch (None, 37, 51, 64)        256
_________________________________________________________________
conv2d_4 (Conv2D)            (None, 36, 50, 64)        16448
_________________________________________________________________
max_pooling2d_1 (MaxPooling2 (None, 18, 25, 64)        0
_________________________________________________________________
```

```
batch_normalization_4 (Batch (None, 18, 25, 64)        256
_________________________________________________________________
conv2d_5 (Conv2D)            (None, 17, 24, 128)       32896
_________________________________________________________________
batch_normalization_5 (Batch (None, 17, 24, 128)       512
_________________________________________________________________
conv2d_6 (Conv2D)            (None, 16, 23, 128)       65664
_________________________________________________________________
batch_normalization_6 (Batch (None, 16, 23, 128)       512
_________________________________________________________________
conv2d_7 (Conv2D)            (None, 15, 22, 128)       65664
_________________________________________________________________
max_pooling2d_2 (MaxPooling2 (None, 7, 11, 128)        0
_________________________________________________________________
batch_normalization_7 (Batch (None, 7, 11, 128)        512
_________________________________________________________________
conv2d_8 (Conv2D)            (None, 6, 10, 256)        131328
_________________________________________________________________
batch_normalization_8 (Batch (None, 6, 10, 256)        1024
_________________________________________________________________
conv2d_9 (Conv2D)            (None, 5, 9, 256)         262400
_________________________________________________________________
max_pooling2d_3 (MaxPooling2 (None, 2, 4, 256)         0
_________________________________________________________________
flatten (Flatten)            (None, 2048)              0
_________________________________________________________________
dense (Dense)                (None, 64)                131136
_________________________________________________________________
dense_1 (Dense)              (None, 32)                2080
_________________________________________________________________
dense_2 (Dense)              (None, 32)                1056
_________________________________________________________________
dense_3 (Dense)              (None, 16)                528
_________________________________________________________________
dense_4 (Dense)              (None, 16)                272
_________________________________________________________________
dense_5 (Dense)              (None, 10)                170
=================================================================
Total params: 742,218
Trainable params: 740,426
Non-trainable params: 1,792
_________________________________________________________________
```

In [11]:

```
model.compile(optimizer= 'Adam',
              loss='mean_squared_error',
              metrics=['mean_absolute_error'])


history = model.fit(T_x, #X_training,
                    Labels_a, #thickness_training,
                    epochs=1000,
                    batch_size=512,
                    verbose=1,
                    validation_split=0.05,

                   )

score, acc = model.evaluate(T_x, Labels_a)

print('Test score:', score)
print('Test accuracy:', acc)
```

```
Epoch 1/1000
1/1 [==============================] - 4s 4s/step - loss: 0.3821 - mean_absolute_error: 0.5533 - val_loss: 0.4322
- val_mean_absolute_error: 0.6279
Epoch 2/1000
1/1 [==============================] - 0s 98ms/step - loss: 0.2906 - mean_absolute_error: 0.4789 - val_loss: 0.43
03 - val_mean_absolute_error: 0.6264
Epoch 3/1000
1/1 [==============================] - 0s 94ms/step - loss: 0.1644 - mean_absolute_error: 0.3373 - val_loss: 0.42
79 - val_mean_absolute_error: 0.6245
Epoch 4/1000
1/1 [==============================] - 0s 95ms/step - loss: 0.1660 - mean_absolute_error: 0.3228 - val_loss: 0.42
68 - val_mean_absolute_error: 0.6237
Epoch 5/1000
1/1 [==============================] - 0s 96ms/step - loss: 0.1419 - mean_absolute_error: 0.2955 - val_loss: 0.42
53 - val_mean_absolute_error: 0.6224
Epoch 6/1000
1/1 [==============================] - 0s 98ms/step - loss: 0.1447 - mean_absolute_error: 0.3114 - val_loss: 0.42
35 - val_mean_absolute_error: 0.6210
Epoch 7/1000
```

```
1/1 [==============================] - 0s 96ms/step - loss: 0.1341 - mean_absolute_error: 0.2830 - val_loss: 0.42
15 - val_mean_absolute_error: 0.6194
Epoch 8/1000
1/1 [==============================] - 0s 99ms/step - loss: 0.1295 - mean_absolute_error: 0.2719 - val_loss: 0.41
91 - val_mean_absolute_error: 0.6175
Epoch 9/1000
1/1 [==============================] - 0s 96ms/step - loss: 0.1235 - mean_absolute_error: 0.2646 - val_loss: 0.41
64 - val_mean_absolute_error: 0.6154
Epoch 10/1000
1/1 [==============================] - 0s 95ms/step - loss: 0.1248 - mean_absolute_error: 0.2716 - val_loss: 0.41
38 - val_mean_absolute_error: 0.6132
Epoch 11/1000
1/1 [==============================] - 0s 94ms/step - loss: 0.1197 - mean_absolute_error: 0.2599 - val_loss: 0.41
07 - val_mean_absolute_error: 0.6106
Epoch 12/1000
1/1 [==============================] - 0s 98ms/step - loss: 0.1164 - mean_absolute_error: 0.2551 - val_loss: 0.40
76 - val_mean_absolute_error: 0.6080
Epoch 13/1000
1/1 [==============================] - 0s 98ms/step - loss: 0.1137 - mean_absolute_error: 0.2521 - val_loss: 0.40
43 - val_mean_absolute_error: 0.6052
Epoch 14/1000
1/1 [==============================] - 0s 96ms/step - loss: 0.1112 - mean_absolute_error: 0.2486 - val_loss: 0.40
08 - val_mean_absolute_error: 0.6023
Epoch 15/1000
1/1 [==============================] - 0s 93ms/step - loss: 0.1093 - mean_absolute_error: 0.2458 - val_loss: 0.39
74 - val_mean_absolute_error: 0.5994
Epoch 16/1000
1/1 [==============================] - 0s 96ms/step - loss: 0.1068 - mean_absolute_error: 0.2421 - val_loss: 0.39
40 - val_mean_absolute_error: 0.5966
Epoch 17/1000
1/1 [==============================] - 0s 99ms/step - loss: 0.1046 - mean_absolute_error: 0.2401 - val_loss: 0.39
09 - val_mean_absolute_error: 0.5939
Epoch 18/1000
1/1 [==============================] - 0s 99ms/step - loss: 0.1026 - mean_absolute_error: 0.2366 - val_loss: 0.38
79 - val_mean_absolute_error: 0.5913
Epoch 19/1000
1/1 [==============================] - 0s 99ms/step - loss: 0.1007 - mean_absolute_error: 0.2335 - val_loss: 0.38
48 - val_mean_absolute_error: 0.5887
Epoch 20/1000
1/1 [==============================] - 0s 97ms/step - loss: 0.0982 - mean_absolute_error: 0.2312 - val_loss: 0.38
18 - val_mean_absolute_error: 0.5862
Epoch 21/1000
1/1 [==============================] - 0s 97ms/step - loss: 0.0954 - mean_absolute_error: 0.2307 - val_loss: 0.37
91 - val_mean_absolute_error: 0.5838
Epoch 22/1000
1/1 [==============================] - 0s 100ms/step - loss: 0.0925 - mean_absolute_error: 0.2283 - val_loss: 0.3
760 - val_mean_absolute_error: 0.5812
Epoch 23/1000
1/1 [==============================] - 0s 96ms/step - loss: 0.0888 - mean_absolute_error: 0.2253 - val_loss: 0.37
26 - val_mean_absolute_error: 0.5784
Epoch 24/1000
1/1 [==============================] - 0s 94ms/step - loss: 0.0852 - mean_absolute_error: 0.2217 - val_loss: 0.36
94 - val_mean_absolute_error: 0.5758
Epoch 25/1000
1/1 [==============================] - 0s 95ms/step - loss: 0.0819 - mean_absolute_error: 0.2178 - val_loss: 0.36
66 - val_mean_absolute_error: 0.5735
Epoch 26/1000
1/1 [==============================] - 0s 94ms/step - loss: 0.0789 - mean_absolute_error: 0.2136 - val_loss: 0.36
39 - val_mean_absolute_error: 0.5714
Epoch 27/1000
1/1 [==============================] - 0s 95ms/step - loss: 0.0757 - mean_absolute_error: 0.2096 - val_loss: 0.36
16 - val_mean_absolute_error: 0.5695
Epoch 28/1000
1/1 [==============================] - 0s 95ms/step - loss: 0.0730 - mean_absolute_error: 0.2053 - val_loss: 0.35
91 - val_mean_absolute_error: 0.5674
Epoch 29/1000
1/1 [==============================] - 0s 95ms/step - loss: 0.0711 - mean_absolute_error: 0.2034 - val_loss: 0.35
63 - val_mean_absolute_error: 0.5650
Epoch 30/1000
1/1 [==============================] - 0s 94ms/step - loss: 0.0693 - mean_absolute_error: 0.2013 - val_loss: 0.35
32 - val_mean_absolute_error: 0.5625
Epoch 31/1000
1/1 [==============================] - 0s 94ms/step - loss: 0.0676 - mean_absolute_error: 0.1991 - val_loss: 0.35
05 - val_mean_absolute_error: 0.5602
Epoch 32/1000
1/1 [==============================] - 0s 93ms/step - loss: 0.0662 - mean_absolute_error: 0.1961 - val_loss: 0.34
83 - val_mean_absolute_error: 0.5582
Epoch 33/1000
1/1 [==============================] - 0s 93ms/step - loss: 0.0650 - mean_absolute_error: 0.1937 - val_loss: 0.34
63 - val_mean_absolute_error: 0.5564
Epoch 34/1000
1/1 [==============================] - 0s 93ms/step - loss: 0.0639 - mean_absolute_error: 0.1914 - val_loss: 0.34
42 - val_mean_absolute_error: 0.5544
```

```
Epoch 35/1000
1/1 [==============================] - 0s 95ms/step - loss: 0.0628 - mean_absolute_error: 0.1891 - val_loss: 0.34
23 - val_mean_absolute_error: 0.5525
Epoch 36/1000
1/1 [==============================] - 0s 92ms/step - loss: 0.0616 - mean_absolute_error: 0.1872 - val_loss: 0.34
03 - val_mean_absolute_error: 0.5507
Epoch 37/1000
1/1 [==============================] - 0s 93ms/step - loss: 0.0606 - mean_absolute_error: 0.1858 - val_loss: 0.33
87 - val_mean_absolute_error: 0.5492
Epoch 38/1000
1/1 [==============================] - 0s 93ms/step - loss: 0.0595 - mean_absolute_error: 0.1833 - val_loss: 0.33
72 - val_mean_absolute_error: 0.5475
Epoch 39/1000
1/1 [==============================] - 0s 103ms/step - loss: 0.0589 - mean_absolute_error: 0.1812 - val_loss: 0.3
343 - val_mean_absolute_error: 0.5450
Epoch 40/1000
1/1 [==============================] - 0s 94ms/step - loss: 0.0579 - mean_absolute_error: 0.1797 - val_loss: 0.33
16 - val_mean_absolute_error: 0.5424
Epoch 41/1000
1/1 [==============================] - 0s 93ms/step - loss: 0.0570 - mean_absolute_error: 0.1778 - val_loss: 0.32
87 - val_mean_absolute_error: 0.5397
Epoch 42/1000
1/1 [==============================] - 0s 91ms/step - loss: 0.0561 - mean_absolute_error: 0.1752 - val_loss: 0.32
55 - val_mean_absolute_error: 0.5367
Epoch 43/1000
1/1 [==============================] - 0s 91ms/step - loss: 0.0555 - mean_absolute_error: 0.1726 - val_loss: 0.32
28 - val_mean_absolute_error: 0.5340
Epoch 44/1000
1/1 [==============================] - 0s 92ms/step - loss: 0.0550 - mean_absolute_error: 0.1704 - val_loss: 0.32
01 - val_mean_absolute_error: 0.5314
Epoch 45/1000
1/1 [==============================] - 0s 91ms/step - loss: 0.0545 - mean_absolute_error: 0.1687 - val_loss: 0.31
75 - val_mean_absolute_error: 0.5290
Epoch 46/1000
1/1 [==============================] - 0s 91ms/step - loss: 0.0541 - mean_absolute_error: 0.1675 - val_loss: 0.31
58 - val_mean_absolute_error: 0.5273
Epoch 47/1000
1/1 [==============================] - 0s 91ms/step - loss: 0.0538 - mean_absolute_error: 0.1666 - val_loss: 0.31
34 - val_mean_absolute_error: 0.5250
Epoch 48/1000
1/1 [==============================] - 0s 91ms/step - loss: 0.0535 - mean_absolute_error: 0.1657 - val_loss: 0.31
14 - val_mean_absolute_error: 0.5229
Epoch 49/1000
1/1 [==============================] - 0s 93ms/step - loss: 0.0533 - mean_absolute_error: 0.1654 - val_loss: 0.30
93 - val_mean_absolute_error: 0.5208
Epoch 50/1000
1/1 [==============================] - 0s 94ms/step - loss: 0.0531 - mean_absolute_error: 0.1650 - val_loss: 0.30
71 - val_mean_absolute_error: 0.5187
Epoch 51/1000
1/1 [==============================] - 0s 91ms/step - loss: 0.0529 - mean_absolute_error: 0.1646 - val_loss: 0.30
58 - val_mean_absolute_error: 0.5170
Epoch 52/1000
1/1 [==============================] - 0s 92ms/step - loss: 0.0528 - mean_absolute_error: 0.1643 - val_loss: 0.30
29 - val_mean_absolute_error: 0.5144
Epoch 53/1000
1/1 [==============================] - 0s 92ms/step - loss: 0.0525 - mean_absolute_error: 0.1641 - val_loss: 0.30
06 - val_mean_absolute_error: 0.5121
Epoch 54/1000
1/1 [==============================] - 0s 92ms/step - loss: 0.0523 - mean_absolute_error: 0.1637 - val_loss: 0.29
90 - val_mean_absolute_error: 0.5103
Epoch 55/1000
1/1 [==============================] - 0s 92ms/step - loss: 0.0521 - mean_absolute_error: 0.1626 - val_loss: 0.29
67 - val_mean_absolute_error: 0.5082
Epoch 56/1000
1/1 [==============================] - 0s 92ms/step - loss: 0.0519 - mean_absolute_error: 0.1619 - val_loss: 0.29
46 - val_mean_absolute_error: 0.5062
Epoch 57/1000
1/1 [==============================] - 0s 92ms/step - loss: 0.0517 - mean_absolute_error: 0.1618 - val_loss: 0.29
25 - val_mean_absolute_error: 0.5042
Epoch 58/1000
1/1 [==============================] - 0s 92ms/step - loss: 0.0515 - mean_absolute_error: 0.1617 - val_loss: 0.29
13 - val_mean_absolute_error: 0.5028
Epoch 59/1000
1/1 [==============================] - 0s 92ms/step - loss: 0.0512 - mean_absolute_error: 0.1605 - val_loss: 0.28
96 - val_mean_absolute_error: 0.5011
Epoch 60/1000
1/1 [==============================] - 0s 92ms/step - loss: 0.0511 - mean_absolute_error: 0.1599 - val_loss: 0.28
79 - val_mean_absolute_error: 0.4995
Epoch 61/1000
1/1 [==============================] - 0s 96ms/step - loss: 0.0508 - mean_absolute_error: 0.1594 - val_loss: 0.28
64 - val_mean_absolute_error: 0.4981
Epoch 62/1000
1/1 [==============================] - 0s 96ms/step - loss: 0.0506 - mean_absolute_error: 0.1589 - val_loss: 0.28
```

```
56 - val_mean_absolute_error: 0.4971
Epoch 63/1000
1/1 [==============================] - 0s 91ms/step - loss: 0.0505 - mean_absolute_error: 0.1583 - val_loss: 0.28
33 - val_mean_absolute_error: 0.4951
Epoch 64/1000
1/1 [==============================] - 0s 91ms/step - loss: 0.0503 - mean_absolute_error: 0.1581 - val_loss: 0.28
26 - val_mean_absolute_error: 0.4941
Epoch 65/1000
1/1 [==============================] - 0s 91ms/step - loss: 0.0504 - mean_absolute_error: 0.1582 - val_loss: 0.27
96 - val_mean_absolute_error: 0.4917
Epoch 66/1000
1/1 [==============================] - 0s 91ms/step - loss: 0.0503 - mean_absolute_error: 0.1583 - val_loss: 0.27
97 - val_mean_absolute_error: 0.4912
Epoch 67/1000
1/1 [==============================] - 0s 92ms/step - loss: 0.0501 - mean_absolute_error: 0.1575 - val_loss: 0.27
71 - val_mean_absolute_error: 0.4891
Epoch 68/1000
1/1 [==============================] - 0s 92ms/step - loss: 0.0497 - mean_absolute_error: 0.1565 - val_loss: 0.27
53 - val_mean_absolute_error: 0.4874
Epoch 69/1000
1/1 [==============================] - 0s 94ms/step - loss: 0.0496 - mean_absolute_error: 0.1562 - val_loss: 0.27
51 - val_mean_absolute_error: 0.4867
Epoch 70/1000
1/1 [==============================] - 0s 91ms/step - loss: 0.0497 - mean_absolute_error: 0.1561 - val_loss: 0.27
18 - val_mean_absolute_error: 0.4841
Epoch 71/1000
1/1 [==============================] - 0s 91ms/step - loss: 0.0497 - mean_absolute_error: 0.1562 - val_loss: 0.27
18 - val_mean_absolute_error: 0.4835
Epoch 72/1000
1/1 [==============================] - 0s 91ms/step - loss: 0.0494 - mean_absolute_error: 0.1554 - val_loss: 0.26
99 - val_mean_absolute_error: 0.4818
Epoch 73/1000
1/1 [==============================] - 0s 96ms/step - loss: 0.0490 - mean_absolute_error: 0.1547 - val_loss: 0.26
79 - val_mean_absolute_error: 0.4800
Epoch 74/1000
1/1 [==============================] - 0s 94ms/step - loss: 0.0491 - mean_absolute_error: 0.1549 - val_loss: 0.26
77 - val_mean_absolute_error: 0.4792
Epoch 75/1000
1/1 [==============================] - 0s 91ms/step - loss: 0.0493 - mean_absolute_error: 0.1551 - val_loss: 0.26
40 - val_mean_absolute_error: 0.4762
Epoch 76/1000
1/1 [==============================] - 0s 92ms/step - loss: 0.0491 - mean_absolute_error: 0.1548 - val_loss: 0.26
38 - val_mean_absolute_error: 0.4755
Epoch 77/1000
1/1 [==============================] - 0s 91ms/step - loss: 0.0487 - mean_absolute_error: 0.1533 - val_loss: 0.26
35 - val_mean_absolute_error: 0.4749
Epoch 78/1000
1/1 [==============================] - 0s 91ms/step - loss: 0.0487 - mean_absolute_error: 0.1536 - val_loss: 0.26
15 - val_mean_absolute_error: 0.4732
Epoch 79/1000
1/1 [==============================] - 0s 91ms/step - loss: 0.0487 - mean_absolute_error: 0.1545 - val_loss: 0.26
08 - val_mean_absolute_error: 0.4721
Epoch 80/1000
1/1 [==============================] - 0s 91ms/step - loss: 0.0486 - mean_absolute_error: 0.1528 - val_loss: 0.25
85 - val_mean_absolute_error: 0.4700
Epoch 81/1000
1/1 [==============================] - 0s 91ms/step - loss: 0.0483 - mean_absolute_error: 0.1523 - val_loss: 0.25
69 - val_mean_absolute_error: 0.4683
Epoch 82/1000
1/1 [==============================] - 0s 91ms/step - loss: 0.0484 - mean_absolute_error: 0.1523 - val_loss: 0.25
67 - val_mean_absolute_error: 0.4675
Epoch 83/1000
1/1 [==============================] - 0s 91ms/step - loss: 0.0484 - mean_absolute_error: 0.1525 - val_loss: 0.25
36 - val_mean_absolute_error: 0.4650
Epoch 84/1000
1/1 [==============================] - 0s 91ms/step - loss: 0.0482 - mean_absolute_error: 0.1524 - val_loss: 0.25
32 - val_mean_absolute_error: 0.4645
Epoch 85/1000
1/1 [==============================] - 0s 91ms/step - loss: 0.0481 - mean_absolute_error: 0.1521 - val_loss: 0.25
37 - val_mean_absolute_error: 0.4646
Epoch 86/1000
1/1 [==============================] - 0s 92ms/step - loss: 0.0480 - mean_absolute_error: 0.1517 - val_loss: 0.25
23 - val_mean_absolute_error: 0.4631
Epoch 87/1000
1/1 [==============================] - 0s 91ms/step - loss: 0.0479 - mean_absolute_error: 0.1510 - val_loss: 0.25
07 - val_mean_absolute_error: 0.4616
Epoch 88/1000
1/1 [==============================] - 0s 91ms/step - loss: 0.0478 - mean_absolute_error: 0.1502 - val_loss: 0.24
87 - val_mean_absolute_error: 0.4595
Epoch 89/1000
1/1 [==============================] - 0s 92ms/step - loss: 0.0477 - mean_absolute_error: 0.1499 - val_loss: 0.24
73 - val_mean_absolute_error: 0.4581
Epoch 90/1000
```

```
1/1 [==============================] - 0s 95ms/step - loss: 0.0476 - mean_absolute_error: 0.1499 - val_loss: 0.24
70 - val_mean_absolute_error: 0.4577
Epoch 91/1000
1/1 [==============================] - 0s 94ms/step - loss: 0.0476 - mean_absolute_error: 0.1499 - val_loss: 0.24
48 - val_mean_absolute_error: 0.4555
Epoch 92/1000
1/1 [==============================] - 0s 93ms/step - loss: 0.0474 - mean_absolute_error: 0.1493 - val_loss: 0.24
38 - val_mean_absolute_error: 0.4545
Epoch 93/1000
1/1 [==============================] - 0s 94ms/step - loss: 0.0474 - mean_absolute_error: 0.1491 - val_loss: 0.24
38 - val_mean_absolute_error: 0.4544
Epoch 94/1000
1/1 [==============================] - 0s 94ms/step - loss: 0.0473 - mean_absolute_error: 0.1489 - val_loss: 0.24
26 - val_mean_absolute_error: 0.4531
Epoch 95/1000
1/1 [==============================] - 0s 95ms/step - loss: 0.0472 - mean_absolute_error: 0.1487 - val_loss: 0.24
08 - val_mean_absolute_error: 0.4512
Epoch 96/1000
1/1 [==============================] - 0s 94ms/step - loss: 0.0471 - mean_absolute_error: 0.1486 - val_loss: 0.23
87 - val_mean_absolute_error: 0.4491
Epoch 97/1000
1/1 [==============================] - 0s 99ms/step - loss: 0.0470 - mean_absolute_error: 0.1484 - val_loss: 0.23
79 - val_mean_absolute_error: 0.4482
Epoch 98/1000
1/1 [==============================] - 0s 98ms/step - loss: 0.0470 - mean_absolute_error: 0.1482 - val_loss: 0.23
81 - val_mean_absolute_error: 0.4483
Epoch 99/1000
1/1 [==============================] - 0s 96ms/step - loss: 0.0469 - mean_absolute_error: 0.1479 - val_loss: 0.23
68 - val_mean_absolute_error: 0.4470
Epoch 100/1000
1/1 [==============================] - 0s 96ms/step - loss: 0.0468 - mean_absolute_error: 0.1476 - val_loss: 0.23
56 - val_mean_absolute_error: 0.4458
Epoch 101/1000
1/1 [==============================] - 0s 96ms/step - loss: 0.0468 - mean_absolute_error: 0.1472 - val_loss: 0.23
36 - val_mean_absolute_error: 0.4436
Epoch 102/1000
1/1 [==============================] - 0s 97ms/step - loss: 0.0467 - mean_absolute_error: 0.1477 - val_loss: 0.23
31 - val_mean_absolute_error: 0.4430
Epoch 103/1000
1/1 [==============================] - 0s 97ms/step - loss: 0.0466 - mean_absolute_error: 0.1469 - val_loss: 0.23
15 - val_mean_absolute_error: 0.4413
Epoch 104/1000
1/1 [==============================] - 0s 98ms/step - loss: 0.0466 - mean_absolute_error: 0.1473 - val_loss: 0.23
04 - val_mean_absolute_error: 0.4401
Epoch 105/1000
1/1 [==============================] - 0s 95ms/step - loss: 0.0465 - mean_absolute_error: 0.1469 - val_loss: 0.22
98 - val_mean_absolute_error: 0.4394
Epoch 106/1000
1/1 [==============================] - 0s 93ms/step - loss: 0.0464 - mean_absolute_error: 0.1468 - val_loss: 0.22
86 - val_mean_absolute_error: 0.4382
Epoch 107/1000
1/1 [==============================] - 0s 93ms/step - loss: 0.0464 - mean_absolute_error: 0.1470 - val_loss: 0.22
87 - val_mean_absolute_error: 0.4382
Epoch 108/1000
1/1 [==============================] - 0s 92ms/step - loss: 0.0464 - mean_absolute_error: 0.1463 - val_loss: 0.22
47 - val_mean_absolute_error: 0.4339
Epoch 109/1000
1/1 [==============================] - 0s 92ms/step - loss: 0.0468 - mean_absolute_error: 0.1481 - val_loss: 0.22
62 - val_mean_absolute_error: 0.4356
Epoch 110/1000
1/1 [==============================] - 0s 92ms/step - loss: 0.0468 - mean_absolute_error: 0.1473 - val_loss: 0.22
49 - val_mean_absolute_error: 0.4341
Epoch 111/1000
1/1 [==============================] - 0s 93ms/step - loss: 0.0464 - mean_absolute_error: 0.1469 - val_loss: 0.22
40 - val_mean_absolute_error: 0.4330
Epoch 112/1000
1/1 [==============================] - 0s 95ms/step - loss: 0.0461 - mean_absolute_error: 0.1461 - val_loss: 0.22
27 - val_mean_absolute_error: 0.4316
Epoch 113/1000
1/1 [==============================] - 0s 99ms/step - loss: 0.0462 - mean_absolute_error: 0.1458 - val_loss: 0.22
03 - val_mean_absolute_error: 0.4290
Epoch 114/1000
1/1 [==============================] - 0s 96ms/step - loss: 0.0463 - mean_absolute_error: 0.1463 - val_loss: 0.22
14 - val_mean_absolute_error: 0.4302
Epoch 115/1000
1/1 [==============================] - 0s 92ms/step - loss: 0.0462 - mean_absolute_error: 0.1456 - val_loss: 0.21
95 - val_mean_absolute_error: 0.4281
Epoch 116/1000
1/1 [==============================] - 0s 92ms/step - loss: 0.0460 - mean_absolute_error: 0.1459 - val_loss: 0.21
86 - val_mean_absolute_error: 0.4271
Epoch 117/1000
1/1 [==============================] - 0s 93ms/step - loss: 0.0459 - mean_absolute_error: 0.1452 - val_loss: 0.21
81 - val_mean_absolute_error: 0.4265
```

```
Epoch 118/1000
1/1 [==============================] - 0s 92ms/step - loss: 0.0459 - mean_absolute_error: 0.1443 - val_loss: 0.21
66 - val_mean_absolute_error: 0.4248
Epoch 119/1000
1/1 [==============================] - 0s 92ms/step - loss: 0.0459 - mean_absolute_error: 0.1447 - val_loss: 0.21
84 - val_mean_absolute_error: 0.4266
Epoch 120/1000
1/1 [==============================] - 0s 103ms/step - loss: 0.0458 - mean_absolute_error: 0.1443 - val_loss: 0.2
167 - val_mean_absolute_error: 0.4247
Epoch 121/1000
1/1 [==============================] - 0s 93ms/step - loss: 0.0456 - mean_absolute_error: 0.1445 - val_loss: 0.21
57 - val_mean_absolute_error: 0.4236
Epoch 122/1000
1/1 [==============================] - 0s 92ms/step - loss: 0.0455 - mean_absolute_error: 0.1439 - val_loss: 0.21
58 - val_mean_absolute_error: 0.4236
Epoch 123/1000
1/1 [==============================] - 0s 98ms/step - loss: 0.0456 - mean_absolute_error: 0.1437 - val_loss: 0.21
45 - val_mean_absolute_error: 0.4223
Epoch 124/1000
1/1 [==============================] - 0s 96ms/step - loss: 0.0455 - mean_absolute_error: 0.1437 - val_loss: 0.21
49 - val_mean_absolute_error: 0.4227
Epoch 125/1000
1/1 [==============================] - 0s 98ms/step - loss: 0.0454 - mean_absolute_error: 0.1430 - val_loss: 0.21
34 - val_mean_absolute_error: 0.4209
Epoch 126/1000
1/1 [==============================] - 0s 94ms/step - loss: 0.0453 - mean_absolute_error: 0.1433 - val_loss: 0.21
29 - val_mean_absolute_error: 0.4204
Epoch 127/1000
1/1 [==============================] - 0s 92ms/step - loss: 0.0452 - mean_absolute_error: 0.1428 - val_loss: 0.21
32 - val_mean_absolute_error: 0.4206
Epoch 128/1000
1/1 [==============================] - 0s 95ms/step - loss: 0.0451 - mean_absolute_error: 0.1423 - val_loss: 0.21
22 - val_mean_absolute_error: 0.4195
Epoch 129/1000
1/1 [==============================] - 0s 94ms/step - loss: 0.0451 - mean_absolute_error: 0.1423 - val_loss: 0.21
02 - val_mean_absolute_error: 0.4172
Epoch 130/1000
1/1 [==============================] - 0s 94ms/step - loss: 0.0450 - mean_absolute_error: 0.1418 - val_loss: 0.20
95 - val_mean_absolute_error: 0.4164
Epoch 131/1000
1/1 [==============================] - 0s 95ms/step - loss: 0.0450 - mean_absolute_error: 0.1419 - val_loss: 0.20
92 - val_mean_absolute_error: 0.4160
Epoch 132/1000
1/1 [==============================] - 0s 96ms/step - loss: 0.0449 - mean_absolute_error: 0.1422 - val_loss: 0.20
91 - val_mean_absolute_error: 0.4159
Epoch 133/1000
1/1 [==============================] - 0s 93ms/step - loss: 0.0451 - mean_absolute_error: 0.1416 - val_loss: 0.20
61 - val_mean_absolute_error: 0.4125
Epoch 134/1000
1/1 [==============================] - 0s 93ms/step - loss: 0.0455 - mean_absolute_error: 0.1436 - val_loss: 0.20
71 - val_mean_absolute_error: 0.4135
Epoch 135/1000
1/1 [==============================] - 0s 94ms/step - loss: 0.0462 - mean_absolute_error: 0.1453 - val_loss: 0.20
52 - val_mean_absolute_error: 0.4114
Epoch 136/1000
1/1 [==============================] - 0s 93ms/step - loss: 0.0454 - mean_absolute_error: 0.1439 - val_loss: 0.20
67 - val_mean_absolute_error: 0.4131
Epoch 137/1000
1/1 [==============================] - 0s 93ms/step - loss: 0.0448 - mean_absolute_error: 0.1414 - val_loss: 0.20
61 - val_mean_absolute_error: 0.4124
Epoch 138/1000
1/1 [==============================] - 0s 93ms/step - loss: 0.0451 - mean_absolute_error: 0.1418 - val_loss: 0.20
30 - val_mean_absolute_error: 0.4088
Epoch 139/1000
1/1 [==============================] - 0s 93ms/step - loss: 0.0452 - mean_absolute_error: 0.1420 - val_loss: 0.20
45 - val_mean_absolute_error: 0.4105
Epoch 140/1000
1/1 [==============================] - 0s 92ms/step - loss: 0.0449 - mean_absolute_error: 0.1415 - val_loss: 0.20
42 - val_mean_absolute_error: 0.4102
Epoch 141/1000
1/1 [==============================] - 0s 93ms/step - loss: 0.0449 - mean_absolute_error: 0.1418 - val_loss: 0.20
29 - val_mean_absolute_error: 0.4085
Epoch 142/1000
1/1 [==============================] - 0s 91ms/step - loss: 0.0450 - mean_absolute_error: 0.1427 - val_loss: 0.20
14 - val_mean_absolute_error: 0.4067
Epoch 143/1000
1/1 [==============================] - 0s 93ms/step - loss: 0.0447 - mean_absolute_error: 0.1407 - val_loss: 0.19
97 - val_mean_absolute_error: 0.4047
Epoch 144/1000
1/1 [==============================] - 0s 92ms/step - loss: 0.0445 - mean_absolute_error: 0.1400 - val_loss: 0.20
04 - val_mean_absolute_error: 0.4056
Epoch 145/1000
1/1 [==============================] - 0s 91ms/step - loss: 0.0446 - mean_absolute_error: 0.1405 - val_loss: 0.20
```

```
00 - val_mean_absolute_error: 0.4050
Epoch 146/1000
1/1 [==============================] - 0s 91ms/step - loss: 0.0447 - mean_absolute_error: 0.1407 - val_loss: 0.19
99 - val_mean_absolute_error: 0.4049
Epoch 147/1000
1/1 [==============================] - 0s 92ms/step - loss: 0.0444 - mean_absolute_error: 0.1401 - val_loss: 0.19
88 - val_mean_absolute_error: 0.4036
Epoch 148/1000
1/1 [==============================] - 0s 92ms/step - loss: 0.0442 - mean_absolute_error: 0.1395 - val_loss: 0.19
79 - val_mean_absolute_error: 0.4025
Epoch 149/1000
1/1 [==============================] - 0s 91ms/step - loss: 0.0442 - mean_absolute_error: 0.1392 - val_loss: 0.19
85 - val_mean_absolute_error: 0.4032
Epoch 150/1000
1/1 [==============================] - 0s 92ms/step - loss: 0.0442 - mean_absolute_error: 0.1391 - val_loss: 0.19
76 - val_mean_absolute_error: 0.4022
Epoch 151/1000
1/1 [==============================] - 0s 97ms/step - loss: 0.0441 - mean_absolute_error: 0.1390 - val_loss: 0.19
78 - val_mean_absolute_error: 0.4025
Epoch 152/1000
1/1 [==============================] - 0s 92ms/step - loss: 0.0440 - mean_absolute_error: 0.1388 - val_loss: 0.19
75 - val_mean_absolute_error: 0.4021
Epoch 153/1000
1/1 [==============================] - 0s 97ms/step - loss: 0.0439 - mean_absolute_error: 0.1380 - val_loss: 0.19
54 - val_mean_absolute_error: 0.3994
Epoch 154/1000
1/1 [==============================] - 0s 95ms/step - loss: 0.0439 - mean_absolute_error: 0.1378 - val_loss: 0.19
42 - val_mean_absolute_error: 0.3979
Epoch 155/1000
1/1 [==============================] - 0s 93ms/step - loss: 0.0438 - mean_absolute_error: 0.1375 - val_loss: 0.19
31 - val_mean_absolute_error: 0.3966
Epoch 156/1000
1/1 [==============================] - 0s 93ms/step - loss: 0.0437 - mean_absolute_error: 0.1373 - val_loss: 0.19
34 - val_mean_absolute_error: 0.3970
Epoch 157/1000
1/1 [==============================] - 0s 94ms/step - loss: 0.0436 - mean_absolute_error: 0.1369 - val_loss: 0.19
34 - val_mean_absolute_error: 0.3969
Epoch 158/1000
1/1 [==============================] - 0s 93ms/step - loss: 0.0436 - mean_absolute_error: 0.1365 - val_loss: 0.19
22 - val_mean_absolute_error: 0.3955
Epoch 159/1000
1/1 [==============================] - 0s 93ms/step - loss: 0.0435 - mean_absolute_error: 0.1362 - val_loss: 0.19
22 - val_mean_absolute_error: 0.3955
Epoch 160/1000
1/1 [==============================] - 0s 96ms/step - loss: 0.0435 - mean_absolute_error: 0.1361 - val_loss: 0.19
17 - val_mean_absolute_error: 0.3949
Epoch 161/1000
1/1 [==============================] - 0s 92ms/step - loss: 0.0434 - mean_absolute_error: 0.1361 - val_loss: 0.19
12 - val_mean_absolute_error: 0.3943
Epoch 162/1000
1/1 [==============================] - 0s 93ms/step - loss: 0.0433 - mean_absolute_error: 0.1358 - val_loss: 0.19
02 - val_mean_absolute_error: 0.3931
Epoch 163/1000
1/1 [==============================] - 0s 93ms/step - loss: 0.0433 - mean_absolute_error: 0.1356 - val_loss: 0.18
91 - val_mean_absolute_error: 0.3916
Epoch 164/1000
1/1 [==============================] - 0s 94ms/step - loss: 0.0433 - mean_absolute_error: 0.1356 - val_loss: 0.18
94 - val_mean_absolute_error: 0.3921
Epoch 165/1000
1/1 [==============================] - 0s 92ms/step - loss: 0.0432 - mean_absolute_error: 0.1358 - val_loss: 0.18
81 - val_mean_absolute_error: 0.3904
Epoch 166/1000
1/1 [==============================] - 0s 100ms/step - loss: 0.0432 - mean_absolute_error: 0.1354 - val_loss: 0.1
881 - val_mean_absolute_error: 0.3903
Epoch 167/1000
1/1 [==============================] - 0s 92ms/step - loss: 0.0433 - mean_absolute_error: 0.1356 - val_loss: 0.18
78 - val_mean_absolute_error: 0.3901
Epoch 168/1000
1/1 [==============================] - 0s 92ms/step - loss: 0.0436 - mean_absolute_error: 0.1367 - val_loss: 0.18
76 - val_mean_absolute_error: 0.3898
Epoch 169/1000
1/1 [==============================] - 0s 97ms/step - loss: 0.0433 - mean_absolute_error: 0.1359 - val_loss: 0.18
66 - val_mean_absolute_error: 0.3884
Epoch 170/1000
1/1 [==============================] - 0s 93ms/step - loss: 0.0430 - mean_absolute_error: 0.1347 - val_loss: 0.18
60 - val_mean_absolute_error: 0.3878
Epoch 171/1000
1/1 [==============================] - 0s 92ms/step - loss: 0.0432 - mean_absolute_error: 0.1353 - val_loss: 0.18
43 - val_mean_absolute_error: 0.3854
Epoch 172/1000
1/1 [==============================] - 0s 92ms/step - loss: 0.0432 - mean_absolute_error: 0.1352 - val_loss: 0.18
41 - val_mean_absolute_error: 0.3853
Epoch 173/1000
```

```
1/1 [==============================] - 0s 98ms/step - loss: 0.0428 - mean_absolute_error: 0.1343 - val_loss: 0.18
47 - val_mean_absolute_error: 0.3860
Epoch 174/1000
1/1 [==============================] - 0s 93ms/step - loss: 0.0431 - mean_absolute_error: 0.1356 - val_loss: 0.18
23 - val_mean_absolute_error: 0.3827
Epoch 175/1000
1/1 [==============================] - 0s 94ms/step - loss: 0.0431 - mean_absolute_error: 0.1356 - val_loss: 0.18
30 - val_mean_absolute_error: 0.3840
Epoch 176/1000
1/1 [==============================] - 0s 93ms/step - loss: 0.0429 - mean_absolute_error: 0.1351 - val_loss: 0.18
20 - val_mean_absolute_error: 0.3826
Epoch 177/1000
1/1 [==============================] - 0s 96ms/step - loss: 0.0429 - mean_absolute_error: 0.1349 - val_loss: 0.18
11 - val_mean_absolute_error: 0.3812
Epoch 178/1000
1/1 [==============================] - 0s 94ms/step - loss: 0.0427 - mean_absolute_error: 0.1337 - val_loss: 0.18
12 - val_mean_absolute_error: 0.3813
Epoch 179/1000
1/1 [==============================] - 0s 92ms/step - loss: 0.0427 - mean_absolute_error: 0.1333 - val_loss: 0.18
03 - val_mean_absolute_error: 0.3802
Epoch 180/1000
1/1 [==============================] - 0s 94ms/step - loss: 0.0429 - mean_absolute_error: 0.1342 - val_loss: 0.18
09 - val_mean_absolute_error: 0.3812
Epoch 181/1000
1/1 [==============================] - 0s 92ms/step - loss: 0.0429 - mean_absolute_error: 0.1348 - val_loss: 0.17
93 - val_mean_absolute_error: 0.3786
Epoch 182/1000
1/1 [==============================] - 0s 92ms/step - loss: 0.0428 - mean_absolute_error: 0.1348 - val_loss: 0.18
05 - val_mean_absolute_error: 0.3805
Epoch 183/1000
1/1 [==============================] - 0s 94ms/step - loss: 0.0426 - mean_absolute_error: 0.1336 - val_loss: 0.17
89 - val_mean_absolute_error: 0.3785
Epoch 184/1000
1/1 [==============================] - 0s 94ms/step - loss: 0.0423 - mean_absolute_error: 0.1328 - val_loss: 0.17
81 - val_mean_absolute_error: 0.3773
Epoch 185/1000
1/1 [==============================] - 0s 92ms/step - loss: 0.0423 - mean_absolute_error: 0.1325 - val_loss: 0.17
79 - val_mean_absolute_error: 0.3771
Epoch 186/1000
1/1 [==============================] - 0s 92ms/step - loss: 0.0422 - mean_absolute_error: 0.1317 - val_loss: 0.17
62 - val_mean_absolute_error: 0.3747
Epoch 187/1000
1/1 [==============================] - 0s 91ms/step - loss: 0.0422 - mean_absolute_error: 0.1317 - val_loss: 0.17
64 - val_mean_absolute_error: 0.3752
Epoch 188/1000
1/1 [==============================] - 0s 91ms/step - loss: 0.0421 - mean_absolute_error: 0.1315 - val_loss: 0.17
66 - val_mean_absolute_error: 0.3754
Epoch 189/1000
1/1 [==============================] - 0s 91ms/step - loss: 0.0420 - mean_absolute_error: 0.1311 - val_loss: 0.17
65 - val_mean_absolute_error: 0.3752
Epoch 190/1000
1/1 [==============================] - 0s 94ms/step - loss: 0.0419 - mean_absolute_error: 0.1306 - val_loss: 0.17
63 - val_mean_absolute_error: 0.3749
Epoch 191/1000
1/1 [==============================] - 0s 93ms/step - loss: 0.0419 - mean_absolute_error: 0.1305 - val_loss: 0.17
51 - val_mean_absolute_error: 0.3732
Epoch 192/1000
1/1 [==============================] - 0s 92ms/step - loss: 0.0420 - mean_absolute_error: 0.1308 - val_loss: 0.17
53 - val_mean_absolute_error: 0.3736
Epoch 193/1000
1/1 [==============================] - 0s 92ms/step - loss: 0.0419 - mean_absolute_error: 0.1307 - val_loss: 0.17
34 - val_mean_absolute_error: 0.3706
Epoch 194/1000
1/1 [==============================] - 0s 92ms/step - loss: 0.0420 - mean_absolute_error: 0.1312 - val_loss: 0.17
49 - val_mean_absolute_error: 0.3732
Epoch 195/1000
1/1 [==============================] - 0s 92ms/step - loss: 0.0423 - mean_absolute_error: 0.1321 - val_loss: 0.17
16 - val_mean_absolute_error: 0.3677
Epoch 196/1000
1/1 [==============================] - 0s 92ms/step - loss: 0.0427 - mean_absolute_error: 0.1346 - val_loss: 0.17
26 - val_mean_absolute_error: 0.3695
Epoch 197/1000
1/1 [==============================] - 0s 91ms/step - loss: 0.0418 - mean_absolute_error: 0.1301 - val_loss: 0.17
23 - val_mean_absolute_error: 0.3694
Epoch 198/1000
1/1 [==============================] - 0s 92ms/step - loss: 0.0426 - mean_absolute_error: 0.1337 - val_loss: 0.16
77 - val_mean_absolute_error: 0.3622
Epoch 199/1000
1/1 [==============================] - 0s 92ms/step - loss: 0.0425 - mean_absolute_error: 0.1333 - val_loss: 0.16
83 - val_mean_absolute_error: 0.3637
Epoch 200/1000
1/1 [==============================] - 0s 92ms/step - loss: 0.0426 - mean_absolute_error: 0.1333 - val_loss: 0.16
76 - val_mean_absolute_error: 0.3625
```

```
Epoch 201/1000
1/1 [==============================] - 0s 92ms/step - loss: 0.0427 - mean_absolute_error: 0.1334 - val_loss: 0.16
61 - val_mean_absolute_error: 0.3605
Epoch 202/1000
1/1 [==============================] - 0s 92ms/step - loss: 0.0422 - mean_absolute_error: 0.1317 - val_loss: 0.16
49 - val_mean_absolute_error: 0.3579
Epoch 203/1000
1/1 [==============================] - 0s 92ms/step - loss: 0.0426 - mean_absolute_error: 0.1333 - val_loss: 0.16
64 - val_mean_absolute_error: 0.3614
Epoch 204/1000
1/1 [==============================] - 0s 91ms/step - loss: 0.0425 - mean_absolute_error: 0.1324 - val_loss: 0.16
26 - val_mean_absolute_error: 0.3555
Epoch 205/1000
1/1 [==============================] - 0s 94ms/step - loss: 0.0417 - mean_absolute_error: 0.1292 - val_loss: 0.16
03 - val_mean_absolute_error: 0.3519
Epoch 206/1000
1/1 [==============================] - 0s 94ms/step - loss: 0.0418 - mean_absolute_error: 0.1295 - val_loss: 0.16
20 - val_mean_absolute_error: 0.3549
Epoch 207/1000
1/1 [==============================] - 0s 93ms/step - loss: 0.0417 - mean_absolute_error: 0.1288 - val_loss: 0.16
28 - val_mean_absolute_error: 0.3563
Epoch 208/1000
1/1 [==============================] - 0s 93ms/step - loss: 0.0416 - mean_absolute_error: 0.1288 - val_loss: 0.16
31 - val_mean_absolute_error: 0.3569
Epoch 209/1000
1/1 [==============================] - 0s 94ms/step - loss: 0.0416 - mean_absolute_error: 0.1291 - val_loss: 0.16
22 - val_mean_absolute_error: 0.3554
Epoch 210/1000
1/1 [==============================] - 0s 93ms/step - loss: 0.0414 - mean_absolute_error: 0.1281 - val_loss: 0.16
17 - val_mean_absolute_error: 0.3548
Epoch 211/1000
1/1 [==============================] - 0s 94ms/step - loss: 0.0413 - mean_absolute_error: 0.1279 - val_loss: 0.16
18 - val_mean_absolute_error: 0.3551
Epoch 212/1000
1/1 [==============================] - 0s 92ms/step - loss: 0.0413 - mean_absolute_error: 0.1272 - val_loss: 0.16
12 - val_mean_absolute_error: 0.3537
Epoch 213/1000
1/1 [==============================] - 0s 92ms/step - loss: 0.0412 - mean_absolute_error: 0.1266 - val_loss: 0.16
08 - val_mean_absolute_error: 0.3532
Epoch 214/1000
1/1 [==============================] - 0s 93ms/step - loss: 0.0411 - mean_absolute_error: 0.1266 - val_loss: 0.16
01 - val_mean_absolute_error: 0.3524
Epoch 215/1000
1/1 [==============================] - 0s 91ms/step - loss: 0.0410 - mean_absolute_error: 0.1266 - val_loss: 0.15
87 - val_mean_absolute_error: 0.3502
Epoch 216/1000
1/1 [==============================] - 0s 92ms/step - loss: 0.0410 - mean_absolute_error: 0.1266 - val_loss: 0.15
79 - val_mean_absolute_error: 0.3492
Epoch 217/1000
1/1 [==============================] - 0s 94ms/step - loss: 0.0409 - mean_absolute_error: 0.1265 - val_loss: 0.15
91 - val_mean_absolute_error: 0.3509
Epoch 218/1000
1/1 [==============================] - 0s 92ms/step - loss: 0.0408 - mean_absolute_error: 0.1263 - val_loss: 0.16
04 - val_mean_absolute_error: 0.3529
Epoch 219/1000
1/1 [==============================] - 0s 93ms/step - loss: 0.0408 - mean_absolute_error: 0.1258 - val_loss: 0.15
90 - val_mean_absolute_error: 0.3508
Epoch 220/1000
1/1 [==============================] - 0s 92ms/step - loss: 0.0407 - mean_absolute_error: 0.1257 - val_loss: 0.15
78 - val_mean_absolute_error: 0.3491
Epoch 221/1000
1/1 [==============================] - 0s 92ms/step - loss: 0.0406 - mean_absolute_error: 0.1255 - val_loss: 0.15
65 - val_mean_absolute_error: 0.3467
Epoch 222/1000
1/1 [==============================] - 0s 91ms/step - loss: 0.0406 - mean_absolute_error: 0.1253 - val_loss: 0.15
68 - val_mean_absolute_error: 0.3471
Epoch 223/1000
1/1 [==============================] - 0s 92ms/step - loss: 0.0406 - mean_absolute_error: 0.1251 - val_loss: 0.15
80 - val_mean_absolute_error: 0.3493
Epoch 224/1000
1/1 [==============================] - 0s 101ms/step - loss: 0.0406 - mean_absolute_error: 0.1257 - val_loss: 0.1
565 - val_mean_absolute_error: 0.3461
Epoch 225/1000
1/1 [==============================] - 0s 92ms/step - loss: 0.0409 - mean_absolute_error: 0.1266 - val_loss: 0.15
75 - val_mean_absolute_error: 0.3490
Epoch 226/1000
1/1 [==============================] - 0s 92ms/step - loss: 0.0411 - mean_absolute_error: 0.1276 - val_loss: 0.15
55 - val_mean_absolute_error: 0.3448
Epoch 227/1000
1/1 [==============================] - 0s 92ms/step - loss: 0.0413 - mean_absolute_error: 0.1287 - val_loss: 0.15
78 - val_mean_absolute_error: 0.3491
Epoch 228/1000
1/1 [==============================] - 0s 91ms/step - loss: 0.0405 - mean_absolute_error: 0.1243 - val_loss: 0.15
```

```
77 - val_mean_absolute_error: 0.3491
Epoch 229/1000
1/1 [==============================] - 0s 91ms/step - loss: 0.0404 - mean_absolute_error: 0.1244 - val_loss: 0.15
64 - val_mean_absolute_error: 0.3463
Epoch 230/1000
1/1 [==============================] - 0s 99ms/step - loss: 0.0409 - mean_absolute_error: 0.1269 - val_loss: 0.15
75 - val_mean_absolute_error: 0.3489
Epoch 231/1000
1/1 [==============================] - 0s 92ms/step - loss: 0.0407 - mean_absolute_error: 0.1252 - val_loss: 0.15
51 - val_mean_absolute_error: 0.3446
Epoch 232/1000
1/1 [==============================] - 0s 91ms/step - loss: 0.0403 - mean_absolute_error: 0.1232 - val_loss: 0.15
41 - val_mean_absolute_error: 0.3431
Epoch 233/1000
1/1 [==============================] - 0s 91ms/step - loss: 0.0402 - mean_absolute_error: 0.1230 - val_loss: 0.15
36 - val_mean_absolute_error: 0.3430
Epoch 234/1000
1/1 [==============================] - 0s 92ms/step - loss: 0.0406 - mean_absolute_error: 0.1247 - val_loss: 0.15
09 - val_mean_absolute_error: 0.3383
Epoch 235/1000
1/1 [==============================] - 0s 92ms/step - loss: 0.0408 - mean_absolute_error: 0.1266 - val_loss: 0.15
32 - val_mean_absolute_error: 0.3420
Epoch 236/1000
1/1 [==============================] - 0s 92ms/step - loss: 0.0402 - mean_absolute_error: 0.1225 - val_loss: 0.15
28 - val_mean_absolute_error: 0.3417
Epoch 237/1000
1/1 [==============================] - 0s 92ms/step - loss: 0.0404 - mean_absolute_error: 0.1232 - val_loss: 0.15
01 - val_mean_absolute_error: 0.3373
Epoch 238/1000
1/1 [==============================] - 0s 92ms/step - loss: 0.0407 - mean_absolute_error: 0.1261 - val_loss: 0.15
01 - val_mean_absolute_error: 0.3379
Epoch 239/1000
1/1 [==============================] - 0s 93ms/step - loss: 0.0402 - mean_absolute_error: 0.1235 - val_loss: 0.14
90 - val_mean_absolute_error: 0.3359
Epoch 240/1000
1/1 [==============================] - 0s 93ms/step - loss: 0.0408 - mean_absolute_error: 0.1241 - val_loss: 0.14
73 - val_mean_absolute_error: 0.3336
Epoch 241/1000
1/1 [==============================] - 0s 93ms/step - loss: 0.0407 - mean_absolute_error: 0.1254 - val_loss: 0.14
79 - val_mean_absolute_error: 0.3349
Epoch 242/1000
1/1 [==============================] - 0s 92ms/step - loss: 0.0409 - mean_absolute_error: 0.1274 - val_loss: 0.14
78 - val_mean_absolute_error: 0.3337
Epoch 243/1000
1/1 [==============================] - 0s 92ms/step - loss: 0.0408 - mean_absolute_error: 0.1249 - val_loss: 0.14
81 - val_mean_absolute_error: 0.3351
Epoch 244/1000
1/1 [==============================] - 0s 92ms/step - loss: 0.0406 - mean_absolute_error: 0.1247 - val_loss: 0.14
46 - val_mean_absolute_error: 0.3292
Epoch 245/1000
1/1 [==============================] - 0s 92ms/step - loss: 0.0404 - mean_absolute_error: 0.1250 - val_loss: 0.14
38 - val_mean_absolute_error: 0.3284
Epoch 246/1000
1/1 [==============================] - 0s 93ms/step - loss: 0.0404 - mean_absolute_error: 0.1242 - val_loss: 0.14
76 - val_mean_absolute_error: 0.3343
Epoch 247/1000
1/1 [==============================] - 0s 95ms/step - loss: 0.0403 - mean_absolute_error: 0.1225 - val_loss: 0.14
67 - val_mean_absolute_error: 0.3330
Epoch 248/1000
1/1 [==============================] - 0s 94ms/step - loss: 0.0403 - mean_absolute_error: 0.1226 - val_loss: 0.14
37 - val_mean_absolute_error: 0.3285
Epoch 249/1000
1/1 [==============================] - 0s 92ms/step - loss: 0.0400 - mean_absolute_error: 0.1224 - val_loss: 0.14
37 - val_mean_absolute_error: 0.3285
Epoch 250/1000
1/1 [==============================] - 0s 92ms/step - loss: 0.0399 - mean_absolute_error: 0.1221 - val_loss: 0.14
41 - val_mean_absolute_error: 0.3291
Epoch 251/1000
1/1 [==============================] - 0s 91ms/step - loss: 0.0399 - mean_absolute_error: 0.1215 - val_loss: 0.14
51 - val_mean_absolute_error: 0.3306
Epoch 252/1000
1/1 [==============================] - 0s 92ms/step - loss: 0.0397 - mean_absolute_error: 0.1206 - val_loss: 0.14
46 - val_mean_absolute_error: 0.3298
Epoch 253/1000
1/1 [==============================] - 0s 91ms/step - loss: 0.0397 - mean_absolute_error: 0.1206 - val_loss: 0.14
22 - val_mean_absolute_error: 0.3262
Epoch 254/1000
1/1 [==============================] - 0s 92ms/step - loss: 0.0395 - mean_absolute_error: 0.1197 - val_loss: 0.14
09 - val_mean_absolute_error: 0.3244
Epoch 255/1000
1/1 [==============================] - 0s 92ms/step - loss: 0.0396 - mean_absolute_error: 0.1203 - val_loss: 0.14
00 - val_mean_absolute_error: 0.3229
Epoch 256/1000
```

```
1/1 [==============================] - 0s 92ms/step - loss: 0.0394 - mean_absolute_error: 0.1197 - val_loss: 0.14
05 - val_mean_absolute_error: 0.3235
Epoch 257/1000
1/1 [==============================] - 0s 92ms/step - loss: 0.0394 - mean_absolute_error: 0.1195 - val_loss: 0.14
16 - val_mean_absolute_error: 0.3251
Epoch 258/1000
1/1 [==============================] - 0s 99ms/step - loss: 0.0393 - mean_absolute_error: 0.1191 - val_loss: 0.14
06 - val_mean_absolute_error: 0.3234
Epoch 259/1000
1/1 [==============================] - 0s 93ms/step - loss: 0.0393 - mean_absolute_error: 0.1188 - val_loss: 0.13
81 - val_mean_absolute_error: 0.3194
Epoch 260/1000
1/1 [==============================] - 0s 94ms/step - loss: 0.0393 - mean_absolute_error: 0.1189 - val_loss: 0.13
76 - val_mean_absolute_error: 0.3187
Epoch 261/1000
1/1 [==============================] - 0s 92ms/step - loss: 0.0393 - mean_absolute_error: 0.1187 - val_loss: 0.13
72 - val_mean_absolute_error: 0.3176
Epoch 262/1000
1/1 [==============================] - 0s 92ms/step - loss: 0.0392 - mean_absolute_error: 0.1188 - val_loss: 0.13
86 - val_mean_absolute_error: 0.3198
Epoch 263/1000
1/1 [==============================] - 0s 93ms/step - loss: 0.0392 - mean_absolute_error: 0.1184 - val_loss: 0.13
86 - val_mean_absolute_error: 0.3202
Epoch 264/1000
1/1 [==============================] - 0s 92ms/step - loss: 0.0392 - mean_absolute_error: 0.1189 - val_loss: 0.13
66 - val_mean_absolute_error: 0.3167
Epoch 265/1000
1/1 [==============================] - 0s 92ms/step - loss: 0.0392 - mean_absolute_error: 0.1185 - val_loss: 0.13
71 - val_mean_absolute_error: 0.3177
Epoch 266/1000
1/1 [==============================] - 0s 93ms/step - loss: 0.0391 - mean_absolute_error: 0.1182 - val_loss: 0.13
62 - val_mean_absolute_error: 0.3163
Epoch 267/1000
1/1 [==============================] - 0s 94ms/step - loss: 0.0391 - mean_absolute_error: 0.1176 - val_loss: 0.13
48 - val_mean_absolute_error: 0.3140
Epoch 268/1000
1/1 [==============================] - 0s 93ms/step - loss: 0.0390 - mean_absolute_error: 0.1176 - val_loss: 0.13
38 - val_mean_absolute_error: 0.3128
Epoch 269/1000
1/1 [==============================] - 0s 93ms/step - loss: 0.0390 - mean_absolute_error: 0.1176 - val_loss: 0.13
41 - val_mean_absolute_error: 0.3131
Epoch 270/1000
1/1 [==============================] - 0s 93ms/step - loss: 0.0390 - mean_absolute_error: 0.1174 - val_loss: 0.13
45 - val_mean_absolute_error: 0.3140
Epoch 271/1000
1/1 [==============================] - 0s 92ms/step - loss: 0.0389 - mean_absolute_error: 0.1175 - val_loss: 0.13
46 - val_mean_absolute_error: 0.3138
Epoch 272/1000
1/1 [==============================] - 0s 102ms/step - loss: 0.0390 - mean_absolute_error: 0.1178 - val_loss: 0.1
332 - val_mean_absolute_error: 0.3120
Epoch 273/1000
1/1 [==============================] - 0s 92ms/step - loss: 0.0390 - mean_absolute_error: 0.1178 - val_loss: 0.13
31 - val_mean_absolute_error: 0.3114
Epoch 274/1000
1/1 [==============================] - 0s 92ms/step - loss: 0.0392 - mean_absolute_error: 0.1177 - val_loss: 0.13
31 - val_mean_absolute_error: 0.3123
Epoch 275/1000
1/1 [==============================] - 0s 93ms/step - loss: 0.0394 - mean_absolute_error: 0.1199 - val_loss: 0.13
46 - val_mean_absolute_error: 0.3140
Epoch 276/1000
1/1 [==============================] - 0s 93ms/step - loss: 0.0395 - mean_absolute_error: 0.1198 - val_loss: 0.13
33 - val_mean_absolute_error: 0.3125
Epoch 277/1000
1/1 [==============================] - 0s 94ms/step - loss: 0.0399 - mean_absolute_error: 0.1228 - val_loss: 0.13
29 - val_mean_absolute_error: 0.3109
Epoch 278/1000
1/1 [==============================] - 0s 93ms/step - loss: 0.0395 - mean_absolute_error: 0.1211 - val_loss: 0.13
16 - val_mean_absolute_error: 0.3093
Epoch 279/1000
1/1 [==============================] - 0s 93ms/step - loss: 0.0393 - mean_absolute_error: 0.1190 - val_loss: 0.13
15 - val_mean_absolute_error: 0.3095
Epoch 280/1000
1/1 [==============================] - 0s 93ms/step - loss: 0.0389 - mean_absolute_error: 0.1174 - val_loss: 0.13
12 - val_mean_absolute_error: 0.3085
Epoch 281/1000
1/1 [==============================] - 0s 93ms/step - loss: 0.0394 - mean_absolute_error: 0.1200 - val_loss: 0.13
01 - val_mean_absolute_error: 0.3074
Epoch 282/1000
1/1 [==============================] - 0s 92ms/step - loss: 0.0401 - mean_absolute_error: 0.1244 - val_loss: 0.13
04 - val_mean_absolute_error: 0.3069
Epoch 283/1000
1/1 [==============================] - 0s 93ms/step - loss: 0.0393 - mean_absolute_error: 0.1179 - val_loss: 0.13
04 - val_mean_absolute_error: 0.3073
```

```
Epoch 284/1000
1/1 [==============================] - 0s 92ms/step - loss: 0.0392 - mean_absolute_error: 0.1175 - val_loss: 0.12
85 - val_mean_absolute_error: 0.3046
Epoch 285/1000
1/1 [==============================] - 0s 92ms/step - loss: 0.0398 - mean_absolute_error: 0.1222 - val_loss: 0.12
63 - val_mean_absolute_error: 0.2991
Epoch 286/1000
1/1 [==============================] - 0s 92ms/step - loss: 0.0398 - mean_absolute_error: 0.1216 - val_loss: 0.12
75 - val_mean_absolute_error: 0.3028
Epoch 287/1000
1/1 [==============================] - 0s 92ms/step - loss: 0.0391 - mean_absolute_error: 0.1186 - val_loss: 0.12
62 - val_mean_absolute_error: 0.3006
Epoch 288/1000
1/1 [==============================] - 0s 93ms/step - loss: 0.0393 - mean_absolute_error: 0.1193 - val_loss: 0.12
49 - val_mean_absolute_error: 0.2968
Epoch 289/1000
1/1 [==============================] - 0s 92ms/step - loss: 0.0395 - mean_absolute_error: 0.1194 - val_loss: 0.12
62 - val_mean_absolute_error: 0.3006
Epoch 290/1000
1/1 [==============================] - 0s 92ms/step - loss: 0.0391 - mean_absolute_error: 0.1187 - val_loss: 0.12
57 - val_mean_absolute_error: 0.2995
Epoch 291/1000
1/1 [==============================] - 0s 92ms/step - loss: 0.0388 - mean_absolute_error: 0.1168 - val_loss: 0.12
31 - val_mean_absolute_error: 0.2940
Epoch 292/1000
1/1 [==============================] - 0s 93ms/step - loss: 0.0391 - mean_absolute_error: 0.1176 - val_loss: 0.12
32 - val_mean_absolute_error: 0.2953
Epoch 293/1000
1/1 [==============================] - 0s 96ms/step - loss: 0.0390 - mean_absolute_error: 0.1180 - val_loss: 0.12
40 - val_mean_absolute_error: 0.2963
Epoch 294/1000
1/1 [==============================] - 0s 94ms/step - loss: 0.0388 - mean_absolute_error: 0.1159 - val_loss: 0.12
19 - val_mean_absolute_error: 0.2915
Epoch 295/1000
1/1 [==============================] - 0s 94ms/step - loss: 0.0388 - mean_absolute_error: 0.1165 - val_loss: 0.12
18 - val_mean_absolute_error: 0.2922
Epoch 296/1000
1/1 [==============================] - 0s 92ms/step - loss: 0.0387 - mean_absolute_error: 0.1161 - val_loss: 0.12
11 - val_mean_absolute_error: 0.2907
Epoch 297/1000
1/1 [==============================] - 0s 97ms/step - loss: 0.0387 - mean_absolute_error: 0.1162 - val_loss: 0.11
86 - val_mean_absolute_error: 0.2855
Epoch 298/1000
1/1 [==============================] - 0s 92ms/step - loss: 0.0390 - mean_absolute_error: 0.1179 - val_loss: 0.12
09 - val_mean_absolute_error: 0.2901
Epoch 299/1000
1/1 [==============================] - 0s 91ms/step - loss: 0.0386 - mean_absolute_error: 0.1161 - val_loss: 0.12
10 - val_mean_absolute_error: 0.2900
Epoch 300/1000
1/1 [==============================] - 0s 91ms/step - loss: 0.0387 - mean_absolute_error: 0.1163 - val_loss: 0.11
85 - val_mean_absolute_error: 0.2850
Epoch 301/1000
1/1 [==============================] - 0s 91ms/step - loss: 0.0392 - mean_absolute_error: 0.1184 - val_loss: 0.11
93 - val_mean_absolute_error: 0.2868
Epoch 302/1000
1/1 [==============================] - 0s 91ms/step - loss: 0.0387 - mean_absolute_error: 0.1148 - val_loss: 0.11
89 - val_mean_absolute_error: 0.2862
Epoch 303/1000
1/1 [==============================] - 0s 92ms/step - loss: 0.0388 - mean_absolute_error: 0.1167 - val_loss: 0.11
65 - val_mean_absolute_error: 0.2810
Epoch 304/1000
1/1 [==============================] - 0s 92ms/step - loss: 0.0388 - mean_absolute_error: 0.1160 - val_loss: 0.11
75 - val_mean_absolute_error: 0.2830
Epoch 305/1000
1/1 [==============================] - 0s 92ms/step - loss: 0.0387 - mean_absolute_error: 0.1147 - val_loss: 0.11
82 - val_mean_absolute_error: 0.2850
Epoch 306/1000
1/1 [==============================] - 0s 93ms/step - loss: 0.0387 - mean_absolute_error: 0.1156 - val_loss: 0.11
57 - val_mean_absolute_error: 0.2789
Epoch 307/1000
1/1 [==============================] - 0s 92ms/step - loss: 0.0385 - mean_absolute_error: 0.1148 - val_loss: 0.11
44 - val_mean_absolute_error: 0.2770
Epoch 308/1000
1/1 [==============================] - 0s 92ms/step - loss: 0.0385 - mean_absolute_error: 0.1142 - val_loss: 0.11
49 - val_mean_absolute_error: 0.2786
Epoch 309/1000
1/1 [==============================] - 0s 94ms/step - loss: 0.0385 - mean_absolute_error: 0.1155 - val_loss: 0.11
40 - val_mean_absolute_error: 0.2762
Epoch 310/1000
1/1 [==============================] - 0s 94ms/step - loss: 0.0385 - mean_absolute_error: 0.1150 - val_loss: 0.11
33 - val_mean_absolute_error: 0.2752
Epoch 311/1000
1/1 [==============================] - 0s 92ms/step - loss: 0.0384 - mean_absolute_error: 0.1143 - val_loss: 0.11
```

```
41 - val_mean_absolute_error: 0.2771
Epoch 312/1000
1/1 [==============================] - 0s 92ms/step - loss: 0.0384 - mean_absolute_error: 0.1146 - val_loss: 0.11
32 - val_mean_absolute_error: 0.2751
Epoch 313/1000
1/1 [==============================] - 0s 92ms/step - loss: 0.0384 - mean_absolute_error: 0.1138 - val_loss: 0.11
19 - val_mean_absolute_error: 0.2727
Epoch 314/1000
1/1 [==============================] - 0s 93ms/step - loss: 0.0385 - mean_absolute_error: 0.1148 - val_loss: 0.11
23 - val_mean_absolute_error: 0.2733
Epoch 315/1000
1/1 [==============================] - 0s 95ms/step - loss: 0.0383 - mean_absolute_error: 0.1137 - val_loss: 0.11
13 - val_mean_absolute_error: 0.2715
Epoch 316/1000
1/1 [==============================] - 0s 93ms/step - loss: 0.0382 - mean_absolute_error: 0.1132 - val_loss: 0.11
03 - val_mean_absolute_error: 0.2700
Epoch 317/1000
1/1 [==============================] - 0s 92ms/step - loss: 0.0383 - mean_absolute_error: 0.1136 - val_loss: 0.11
12 - val_mean_absolute_error: 0.2716
Epoch 318/1000
1/1 [==============================] - 0s 92ms/step - loss: 0.0382 - mean_absolute_error: 0.1129 - val_loss: 0.11
11 - val_mean_absolute_error: 0.2716
Epoch 319/1000
1/1 [==============================] - 0s 92ms/step - loss: 0.0382 - mean_absolute_error: 0.1130 - val_loss: 0.10
96 - val_mean_absolute_error: 0.2686
Epoch 320/1000
1/1 [==============================] - 0s 92ms/step - loss: 0.0382 - mean_absolute_error: 0.1135 - val_loss: 0.10
97 - val_mean_absolute_error: 0.2689
Epoch 321/1000
1/1 [==============================] - 0s 92ms/step - loss: 0.0382 - mean_absolute_error: 0.1133 - val_loss: 0.10
99 - val_mean_absolute_error: 0.2696
Epoch 322/1000
1/1 [==============================] - 0s 93ms/step - loss: 0.0382 - mean_absolute_error: 0.1134 - val_loss: 0.10
93 - val_mean_absolute_error: 0.2682
Epoch 323/1000
1/1 [==============================] - 0s 93ms/step - loss: 0.0382 - mean_absolute_error: 0.1128 - val_loss: 0.10
95 - val_mean_absolute_error: 0.2689
Epoch 324/1000
1/1 [==============================] - 0s 94ms/step - loss: 0.0382 - mean_absolute_error: 0.1130 - val_loss: 0.10
96 - val_mean_absolute_error: 0.2689
Epoch 325/1000
1/1 [==============================] - 0s 93ms/step - loss: 0.0382 - mean_absolute_error: 0.1128 - val_loss: 0.10
91 - val_mean_absolute_error: 0.2678
Epoch 326/1000
1/1 [==============================] - 0s 95ms/step - loss: 0.0381 - mean_absolute_error: 0.1128 - val_loss: 0.10
92 - val_mean_absolute_error: 0.2678
Epoch 327/1000
1/1 [==============================] - 0s 95ms/step - loss: 0.0381 - mean_absolute_error: 0.1131 - val_loss: 0.10
97 - val_mean_absolute_error: 0.2692
Epoch 328/1000
1/1 [==============================] - 0s 95ms/step - loss: 0.0381 - mean_absolute_error: 0.1126 - val_loss: 0.10
99 - val_mean_absolute_error: 0.2695
Epoch 329/1000
1/1 [==============================] - 0s 93ms/step - loss: 0.0380 - mean_absolute_error: 0.1118 - val_loss: 0.10
97 - val_mean_absolute_error: 0.2688
Epoch 330/1000
1/1 [==============================] - 0s 94ms/step - loss: 0.0381 - mean_absolute_error: 0.1123 - val_loss: 0.10
85 - val_mean_absolute_error: 0.2668
Epoch 331/1000
1/1 [==============================] - 0s 93ms/step - loss: 0.0382 - mean_absolute_error: 0.1136 - val_loss: 0.10
78 - val_mean_absolute_error: 0.2653
Epoch 332/1000
1/1 [==============================] - 0s 92ms/step - loss: 0.0381 - mean_absolute_error: 0.1128 - val_loss: 0.10
71 - val_mean_absolute_error: 0.2643
Epoch 333/1000
1/1 [==============================] - 0s 92ms/step - loss: 0.0382 - mean_absolute_error: 0.1133 - val_loss: 0.10
68 - val_mean_absolute_error: 0.2627
Epoch 334/1000
1/1 [==============================] - 0s 93ms/step - loss: 0.0382 - mean_absolute_error: 0.1127 - val_loss: 0.10
74 - val_mean_absolute_error: 0.2645
Epoch 335/1000
1/1 [==============================] - 0s 92ms/step - loss: 0.0381 - mean_absolute_error: 0.1121 - val_loss: 0.10
66 - val_mean_absolute_error: 0.2631
Epoch 336/1000
1/1 [==============================] - 0s 92ms/step - loss: 0.0381 - mean_absolute_error: 0.1132 - val_loss: 0.10
46 - val_mean_absolute_error: 0.2582
Epoch 337/1000
1/1 [==============================] - 0s 92ms/step - loss: 0.0382 - mean_absolute_error: 0.1132 - val_loss: 0.10
57 - val_mean_absolute_error: 0.2616
Epoch 338/1000
1/1 [==============================] - 0s 91ms/step - loss: 0.0381 - mean_absolute_error: 0.1130 - val_loss: 0.10
63 - val_mean_absolute_error: 0.2627
Epoch 339/1000
```

```
1/1 [==============================] - 0s 91ms/step - loss: 0.0380 - mean_absolute_error: 0.1119 - val_loss: 0.10
58 - val_mean_absolute_error: 0.2600
Epoch 340/1000
1/1 [==============================] - 0s 91ms/step - loss: 0.0382 - mean_absolute_error: 0.1131 - val_loss: 0.10
59 - val_mean_absolute_error: 0.2620
Epoch 341/1000
1/1 [==============================] - 0s 91ms/step - loss: 0.0381 - mean_absolute_error: 0.1130 - val_loss: 0.10
44 - val_mean_absolute_error: 0.2584
Epoch 342/1000
1/1 [==============================] - 0s 91ms/step - loss: 0.0381 - mean_absolute_error: 0.1124 - val_loss: 0.10
36 - val_mean_absolute_error: 0.2565
Epoch 343/1000
1/1 [==============================] - 0s 91ms/step - loss: 0.0383 - mean_absolute_error: 0.1139 - val_loss: 0.10
52 - val_mean_absolute_error: 0.2604
Epoch 344/1000
1/1 [==============================] - 0s 91ms/step - loss: 0.0381 - mean_absolute_error: 0.1123 - val_loss: 0.10
60 - val_mean_absolute_error: 0.2621
Epoch 345/1000
1/1 [==============================] - 0s 91ms/step - loss: 0.0380 - mean_absolute_error: 0.1121 - val_loss: 0.10
41 - val_mean_absolute_error: 0.2571
Epoch 346/1000
1/1 [==============================] - 0s 92ms/step - loss: 0.0380 - mean_absolute_error: 0.1122 - val_loss: 0.10
29 - val_mean_absolute_error: 0.2555
Epoch 347/1000
1/1 [==============================] - 0s 91ms/step - loss: 0.0380 - mean_absolute_error: 0.1119 - val_loss: 0.10
34 - val_mean_absolute_error: 0.2565
Epoch 348/1000
1/1 [==============================] - 0s 93ms/step - loss: 0.0380 - mean_absolute_error: 0.1123 - val_loss: 0.10
33 - val_mean_absolute_error: 0.2553
Epoch 349/1000
1/1 [==============================] - 0s 96ms/step - loss: 0.0380 - mean_absolute_error: 0.1124 - val_loss: 0.10
37 - val_mean_absolute_error: 0.2571
Epoch 350/1000
1/1 [==============================] - 0s 92ms/step - loss: 0.0379 - mean_absolute_error: 0.1119 - val_loss: 0.10
27 - val_mean_absolute_error: 0.2550
Epoch 351/1000
1/1 [==============================] - 0s 92ms/step - loss: 0.0379 - mean_absolute_error: 0.1112 - val_loss: 0.10
20 - val_mean_absolute_error: 0.2535
Epoch 352/1000
1/1 [==============================] - 0s 92ms/step - loss: 0.0378 - mean_absolute_error: 0.1112 - val_loss: 0.10
22 - val_mean_absolute_error: 0.2540
Epoch 353/1000
1/1 [==============================] - 0s 97ms/step - loss: 0.0379 - mean_absolute_error: 0.1117 - val_loss: 0.10
23 - val_mean_absolute_error: 0.2539
Epoch 354/1000
1/1 [==============================] - 0s 92ms/step - loss: 0.0378 - mean_absolute_error: 0.1109 - val_loss: 0.10
25 - val_mean_absolute_error: 0.2548
Epoch 355/1000
1/1 [==============================] - 0s 93ms/step - loss: 0.0378 - mean_absolute_error: 0.1109 - val_loss: 0.10
23 - val_mean_absolute_error: 0.2543
Epoch 356/1000
1/1 [==============================] - 0s 93ms/step - loss: 0.0378 - mean_absolute_error: 0.1108 - val_loss: 0.10
19 - val_mean_absolute_error: 0.2532
Epoch 357/1000
1/1 [==============================] - 0s 92ms/step - loss: 0.0378 - mean_absolute_error: 0.1109 - val_loss: 0.10
20 - val_mean_absolute_error: 0.2539
Epoch 358/1000
1/1 [==============================] - 0s 93ms/step - loss: 0.0379 - mean_absolute_error: 0.1116 - val_loss: 0.10
15 - val_mean_absolute_error: 0.2525
Epoch 359/1000
1/1 [==============================] - 0s 93ms/step - loss: 0.0379 - mean_absolute_error: 0.1113 - val_loss: 0.10
23 - val_mean_absolute_error: 0.2547
Epoch 360/1000
1/1 [==============================] - 0s 93ms/step - loss: 0.0380 - mean_absolute_error: 0.1124 - val_loss: 0.10
20 - val_mean_absolute_error: 0.2534
Epoch 361/1000
1/1 [==============================] - 0s 94ms/step - loss: 0.0379 - mean_absolute_error: 0.1113 - val_loss: 0.10
20 - val_mean_absolute_error: 0.2536
Epoch 362/1000
1/1 [==============================] - 0s 93ms/step - loss: 0.0378 - mean_absolute_error: 0.1112 - val_loss: 0.10
09 - val_mean_absolute_error: 0.2514
Epoch 363/1000
1/1 [==============================] - 0s 94ms/step - loss: 0.0378 - mean_absolute_error: 0.1111 - val_loss: 0.10
04 - val_mean_absolute_error: 0.2507
Epoch 364/1000
1/1 [==============================] - 0s 94ms/step - loss: 0.0377 - mean_absolute_error: 0.1108 - val_loss: 0.10
10 - val_mean_absolute_error: 0.2525
Epoch 365/1000
1/1 [==============================] - 0s 93ms/step - loss: 0.0378 - mean_absolute_error: 0.1113 - val_loss: 0.10
03 - val_mean_absolute_error: 0.2499
Epoch 366/1000
1/1 [==============================] - 0s 92ms/step - loss: 0.0379 - mean_absolute_error: 0.1115 - val_loss: 0.10
12 - val_mean_absolute_error: 0.2527
```

```
Epoch 367/1000
1/1 [==============================] - 0s 92ms/step - loss: 0.0379 - mean_absolute_error: 0.1124 - val_loss: 0.09
97 - val_mean_absolute_error: 0.2489
Epoch 368/1000
1/1 [==============================] - 0s 92ms/step - loss: 0.0378 - mean_absolute_error: 0.1111 - val_loss: 0.09
89 - val_mean_absolute_error: 0.2484
Epoch 369/1000
1/1 [==============================] - 0s 92ms/step - loss: 0.0377 - mean_absolute_error: 0.1104 - val_loss: 0.09
82 - val_mean_absolute_error: 0.2467
Epoch 370/1000
1/1 [==============================] - 0s 92ms/step - loss: 0.0377 - mean_absolute_error: 0.1105 - val_loss: 0.09
81 - val_mean_absolute_error: 0.2459
Epoch 371/1000
1/1 [==============================] - 0s 93ms/step - loss: 0.0377 - mean_absolute_error: 0.1107 - val_loss: 0.09
94 - val_mean_absolute_error: 0.2490
Epoch 372/1000
1/1 [==============================] - 0s 102ms/step - loss: 0.0378 - mean_absolute_error: 0.1108 - val_loss: 0.0
983 - val_mean_absolute_error: 0.2465
Epoch 373/1000
1/1 [==============================] - 0s 93ms/step - loss: 0.0378 - mean_absolute_error: 0.1105 - val_loss: 0.09
72 - val_mean_absolute_error: 0.2450
Epoch 374/1000
1/1 [==============================] - 0s 91ms/step - loss: 0.0377 - mean_absolute_error: 0.1105 - val_loss: 0.09
62 - val_mean_absolute_error: 0.2426
Epoch 375/1000
1/1 [==============================] - 0s 91ms/step - loss: 0.0377 - mean_absolute_error: 0.1102 - val_loss: 0.09
64 - val_mean_absolute_error: 0.2432
Epoch 376/1000
1/1 [==============================] - 0s 92ms/step - loss: 0.0377 - mean_absolute_error: 0.1098 - val_loss: 0.09
66 - val_mean_absolute_error: 0.2435
Epoch 377/1000
1/1 [==============================] - 0s 92ms/step - loss: 0.0377 - mean_absolute_error: 0.1103 - val_loss: 0.09
57 - val_mean_absolute_error: 0.2418
Epoch 378/1000
1/1 [==============================] - 0s 91ms/step - loss: 0.0376 - mean_absolute_error: 0.1103 - val_loss: 0.09
55 - val_mean_absolute_error: 0.2414
Epoch 379/1000
1/1 [==============================] - 0s 91ms/step - loss: 0.0377 - mean_absolute_error: 0.1104 - val_loss: 0.09
43 - val_mean_absolute_error: 0.2390
Epoch 380/1000
1/1 [==============================] - 0s 92ms/step - loss: 0.0376 - mean_absolute_error: 0.1102 - val_loss: 0.09
47 - val_mean_absolute_error: 0.2398
Epoch 381/1000
1/1 [==============================] - 0s 98ms/step - loss: 0.0376 - mean_absolute_error: 0.1099 - val_loss: 0.09
46 - val_mean_absolute_error: 0.2399
Epoch 382/1000
1/1 [==============================] - 0s 92ms/step - loss: 0.0376 - mean_absolute_error: 0.1099 - val_loss: 0.09
40 - val_mean_absolute_error: 0.2386
Epoch 383/1000
1/1 [==============================] - 0s 92ms/step - loss: 0.0376 - mean_absolute_error: 0.1098 - val_loss: 0.09
32 - val_mean_absolute_error: 0.2370
Epoch 384/1000
1/1 [==============================] - 0s 92ms/step - loss: 0.0376 - mean_absolute_error: 0.1096 - val_loss: 0.09
33 - val_mean_absolute_error: 0.2374
Epoch 385/1000
1/1 [==============================] - 0s 92ms/step - loss: 0.0376 - mean_absolute_error: 0.1097 - val_loss: 0.09
30 - val_mean_absolute_error: 0.2368
Epoch 386/1000
1/1 [==============================] - 0s 92ms/step - loss: 0.0376 - mean_absolute_error: 0.1096 - val_loss: 0.09
30 - val_mean_absolute_error: 0.2369
Epoch 387/1000
1/1 [==============================] - 0s 92ms/step - loss: 0.0376 - mean_absolute_error: 0.1099 - val_loss: 0.09
25 - val_mean_absolute_error: 0.2360
Epoch 388/1000
1/1 [==============================] - 0s 92ms/step - loss: 0.0375 - mean_absolute_error: 0.1094 - val_loss: 0.09
23 - val_mean_absolute_error: 0.2355
Epoch 389/1000
1/1 [==============================] - 0s 93ms/step - loss: 0.0376 - mean_absolute_error: 0.1100 - val_loss: 0.09
13 - val_mean_absolute_error: 0.2340
Epoch 390/1000
1/1 [==============================] - 0s 92ms/step - loss: 0.0376 - mean_absolute_error: 0.1095 - val_loss: 0.09
20 - val_mean_absolute_error: 0.2350
Epoch 391/1000
1/1 [==============================] - 0s 92ms/step - loss: 0.0376 - mean_absolute_error: 0.1098 - val_loss: 0.09
13 - val_mean_absolute_error: 0.2340
Epoch 392/1000
1/1 [==============================] - 0s 91ms/step - loss: 0.0376 - mean_absolute_error: 0.1096 - val_loss: 0.09
18 - val_mean_absolute_error: 0.2347
Epoch 393/1000
1/1 [==============================] - 0s 91ms/step - loss: 0.0377 - mean_absolute_error: 0.1104 - val_loss: 0.09
05 - val_mean_absolute_error: 0.2325
Epoch 394/1000
1/1 [==============================] - 0s 91ms/step - loss: 0.0376 - mean_absolute_error: 0.1103 - val_loss: 0.09
```

```
08 - val_mean_absolute_error: 0.2333
Epoch 395/1000
1/1 [==============================] - 0s 91ms/step - loss: 0.0376 - mean_absolute_error: 0.1101 - val_loss: 0.08
98 - val_mean_absolute_error: 0.2311
Epoch 396/1000
1/1 [==============================] - 0s 92ms/step - loss: 0.0375 - mean_absolute_error: 0.1096 - val_loss: 0.08
94 - val_mean_absolute_error: 0.2306
Epoch 397/1000
1/1 [==============================] - 0s 91ms/step - loss: 0.0375 - mean_absolute_error: 0.1096 - val_loss: 0.09
00 - val_mean_absolute_error: 0.2319
Epoch 398/1000
1/1 [==============================] - 0s 92ms/step - loss: 0.0376 - mean_absolute_error: 0.1097 - val_loss: 0.08
88 - val_mean_absolute_error: 0.2292
Epoch 399/1000
1/1 [==============================] - 0s 91ms/step - loss: 0.0375 - mean_absolute_error: 0.1100 - val_loss: 0.08
90 - val_mean_absolute_error: 0.2294
Epoch 400/1000
1/1 [==============================] - 0s 91ms/step - loss: 0.0375 - mean_absolute_error: 0.1093 - val_loss: 0.08
84 - val_mean_absolute_error: 0.2284
Epoch 401/1000
1/1 [==============================] - 0s 91ms/step - loss: 0.0375 - mean_absolute_error: 0.1092 - val_loss: 0.08
79 - val_mean_absolute_error: 0.2275
Epoch 402/1000
1/1 [==============================] - 0s 91ms/step - loss: 0.0375 - mean_absolute_error: 0.1094 - val_loss: 0.08
78 - val_mean_absolute_error: 0.2272
Epoch 403/1000
1/1 [==============================] - 0s 91ms/step - loss: 0.0375 - mean_absolute_error: 0.1093 - val_loss: 0.08
80 - val_mean_absolute_error: 0.2278
Epoch 404/1000
1/1 [==============================] - 0s 91ms/step - loss: 0.0374 - mean_absolute_error: 0.1090 - val_loss: 0.08
75 - val_mean_absolute_error: 0.2272
Epoch 405/1000
1/1 [==============================] - 0s 91ms/step - loss: 0.0374 - mean_absolute_error: 0.1089 - val_loss: 0.08
73 - val_mean_absolute_error: 0.2268
Epoch 406/1000
1/1 [==============================] - 0s 91ms/step - loss: 0.0375 - mean_absolute_error: 0.1092 - val_loss: 0.08
71 - val_mean_absolute_error: 0.2263
Epoch 407/1000
1/1 [==============================] - 0s 91ms/step - loss: 0.0375 - mean_absolute_error: 0.1093 - val_loss: 0.08
63 - val_mean_absolute_error: 0.2252
Epoch 408/1000
1/1 [==============================] - 0s 91ms/step - loss: 0.0374 - mean_absolute_error: 0.1091 - val_loss: 0.08
65 - val_mean_absolute_error: 0.2257
Epoch 409/1000
1/1 [==============================] - 0s 91ms/step - loss: 0.0374 - mean_absolute_error: 0.1088 - val_loss: 0.08
62 - val_mean_absolute_error: 0.2248
Epoch 410/1000
1/1 [==============================] - 0s 92ms/step - loss: 0.0375 - mean_absolute_error: 0.1089 - val_loss: 0.08
60 - val_mean_absolute_error: 0.2246
Epoch 411/1000
1/1 [==============================] - 0s 96ms/step - loss: 0.0374 - mean_absolute_error: 0.1085 - val_loss: 0.08
58 - val_mean_absolute_error: 0.2246
Epoch 412/1000
1/1 [==============================] - 0s 93ms/step - loss: 0.0375 - mean_absolute_error: 0.1091 - val_loss: 0.08
51 - val_mean_absolute_error: 0.2233
Epoch 413/1000
1/1 [==============================] - 0s 91ms/step - loss: 0.0375 - mean_absolute_error: 0.1095 - val_loss: 0.08
62 - val_mean_absolute_error: 0.2258
Epoch 414/1000
1/1 [==============================] - 0s 92ms/step - loss: 0.0376 - mean_absolute_error: 0.1098 - val_loss: 0.08
40 - val_mean_absolute_error: 0.2193
Epoch 415/1000
1/1 [==============================] - 0s 92ms/step - loss: 0.0376 - mean_absolute_error: 0.1105 - val_loss: 0.08
52 - val_mean_absolute_error: 0.2236
Epoch 416/1000
1/1 [==============================] - 0s 91ms/step - loss: 0.0378 - mean_absolute_error: 0.1118 - val_loss: 0.08
33 - val_mean_absolute_error: 0.2185
Epoch 417/1000
1/1 [==============================] - 0s 91ms/step - loss: 0.0378 - mean_absolute_error: 0.1113 - val_loss: 0.08
47 - val_mean_absolute_error: 0.2231
Epoch 418/1000
1/1 [==============================] - 0s 92ms/step - loss: 0.0380 - mean_absolute_error: 0.1126 - val_loss: 0.08
42 - val_mean_absolute_error: 0.2214
Epoch 419/1000
1/1 [==============================] - 0s 93ms/step - loss: 0.0377 - mean_absolute_error: 0.1110 - val_loss: 0.08
50 - val_mean_absolute_error: 0.2243
Epoch 420/1000
1/1 [==============================] - 0s 93ms/step - loss: 0.0377 - mean_absolute_error: 0.1099 - val_loss: 0.08
47 - val_mean_absolute_error: 0.2229
Epoch 421/1000
1/1 [==============================] - 0s 92ms/step - loss: 0.0376 - mean_absolute_error: 0.1109 - val_loss: 0.08
35 - val_mean_absolute_error: 0.2192
Epoch 422/1000
```

```
1/1 [==============================] - 0s 92ms/step - loss: 0.0376 - mean_absolute_error: 0.1106 - val_loss: 0.08
46 - val_mean_absolute_error: 0.2239
Epoch 423/1000
1/1 [==============================] - 0s 92ms/step - loss: 0.0378 - mean_absolute_error: 0.1112 - val_loss: 0.08
34 - val_mean_absolute_error: 0.2198
Epoch 424/1000
1/1 [==============================] - 0s 92ms/step - loss: 0.0375 - mean_absolute_error: 0.1100 - val_loss: 0.08
36 - val_mean_absolute_error: 0.2206
Epoch 425/1000
1/1 [==============================] - 0s 92ms/step - loss: 0.0374 - mean_absolute_error: 0.1092 - val_loss: 0.08
31 - val_mean_absolute_error: 0.2197
Epoch 426/1000
1/1 [==============================] - 0s 92ms/step - loss: 0.0375 - mean_absolute_error: 0.1095 - val_loss: 0.08
26 - val_mean_absolute_error: 0.2173
Epoch 427/1000
1/1 [==============================] - 0s 92ms/step - loss: 0.0375 - mean_absolute_error: 0.1091 - val_loss: 0.08
41 - val_mean_absolute_error: 0.2220
Epoch 428/1000
1/1 [==============================] - 0s 92ms/step - loss: 0.0375 - mean_absolute_error: 0.1097 - val_loss: 0.08
23 - val_mean_absolute_error: 0.2169
Epoch 429/1000
1/1 [==============================] - 0s 92ms/step - loss: 0.0375 - mean_absolute_error: 0.1093 - val_loss: 0.08
26 - val_mean_absolute_error: 0.2195
Epoch 430/1000
1/1 [==============================] - 0s 92ms/step - loss: 0.0374 - mean_absolute_error: 0.1092 - val_loss: 0.08
17 - val_mean_absolute_error: 0.2177
Epoch 431/1000
1/1 [==============================] - 0s 91ms/step - loss: 0.0374 - mean_absolute_error: 0.1085 - val_loss: 0.08
15 - val_mean_absolute_error: 0.2166
Epoch 432/1000
1/1 [==============================] - 0s 91ms/step - loss: 0.0374 - mean_absolute_error: 0.1089 - val_loss: 0.08
25 - val_mean_absolute_error: 0.2189
Epoch 433/1000
1/1 [==============================] - 0s 91ms/step - loss: 0.0374 - mean_absolute_error: 0.1088 - val_loss: 0.08
14 - val_mean_absolute_error: 0.2161
Epoch 434/1000
1/1 [==============================] - 0s 92ms/step - loss: 0.0374 - mean_absolute_error: 0.1084 - val_loss: 0.08
19 - val_mean_absolute_error: 0.2191
Epoch 435/1000
1/1 [==============================] - 0s 93ms/step - loss: 0.0374 - mean_absolute_error: 0.1084 - val_loss: 0.08
13 - val_mean_absolute_error: 0.2175
Epoch 436/1000
1/1 [==============================] - 0s 94ms/step - loss: 0.0373 - mean_absolute_error: 0.1085 - val_loss: 0.08
05 - val_mean_absolute_error: 0.2152
Epoch 437/1000
1/1 [==============================] - 0s 94ms/step - loss: 0.0373 - mean_absolute_error: 0.1081 - val_loss: 0.08
03 - val_mean_absolute_error: 0.2151
Epoch 438/1000
1/1 [==============================] - 0s 92ms/step - loss: 0.0373 - mean_absolute_error: 0.1086 - val_loss: 0.07
98 - val_mean_absolute_error: 0.2124
Epoch 439/1000
1/1 [==============================] - 0s 93ms/step - loss: 0.0373 - mean_absolute_error: 0.1088 - val_loss: 0.08
17 - val_mean_absolute_error: 0.2187
Epoch 440/1000
1/1 [==============================] - 0s 93ms/step - loss: 0.0373 - mean_absolute_error: 0.1082 - val_loss: 0.08
04 - val_mean_absolute_error: 0.2138
Epoch 441/1000
1/1 [==============================] - 0s 96ms/step - loss: 0.0373 - mean_absolute_error: 0.1081 - val_loss: 0.08
01 - val_mean_absolute_error: 0.2141
Epoch 442/1000
1/1 [==============================] - 0s 92ms/step - loss: 0.0373 - mean_absolute_error: 0.1078 - val_loss: 0.08
09 - val_mean_absolute_error: 0.2167
Epoch 443/1000
1/1 [==============================] - 0s 94ms/step - loss: 0.0373 - mean_absolute_error: 0.1081 - val_loss: 0.07
96 - val_mean_absolute_error: 0.2109
Epoch 444/1000
1/1 [==============================] - 0s 93ms/step - loss: 0.0373 - mean_absolute_error: 0.1086 - val_loss: 0.08
05 - val_mean_absolute_error: 0.2149
Epoch 445/1000
1/1 [==============================] - 0s 95ms/step - loss: 0.0373 - mean_absolute_error: 0.1080 - val_loss: 0.07
88 - val_mean_absolute_error: 0.2098
Epoch 446/1000
1/1 [==============================] - 0s 92ms/step - loss: 0.0372 - mean_absolute_error: 0.1078 - val_loss: 0.07
88 - val_mean_absolute_error: 0.2097
Epoch 447/1000
1/1 [==============================] - 0s 92ms/step - loss: 0.0372 - mean_absolute_error: 0.1073 - val_loss: 0.07
93 - val_mean_absolute_error: 0.2115
Epoch 448/1000
1/1 [==============================] - 0s 92ms/step - loss: 0.0372 - mean_absolute_error: 0.1073 - val_loss: 0.07
87 - val_mean_absolute_error: 0.2093
Epoch 449/1000
1/1 [==============================] - 0s 92ms/step - loss: 0.0372 - mean_absolute_error: 0.1075 - val_loss: 0.07
83 - val_mean_absolute_error: 0.2086
```

```
Epoch 450/1000
1/1 [==============================] - 0s 92ms/step - loss: 0.0372 - mean_absolute_error: 0.1077 - val_loss: 0.07
78 - val_mean_absolute_error: 0.2074
Epoch 451/1000
1/1 [==============================] - 0s 92ms/step - loss: 0.0372 - mean_absolute_error: 0.1078 - val_loss: 0.07
89 - val_mean_absolute_error: 0.2117
Epoch 452/1000
1/1 [==============================] - 0s 92ms/step - loss: 0.0372 - mean_absolute_error: 0.1077 - val_loss: 0.07
83 - val_mean_absolute_error: 0.2071
Epoch 453/1000
1/1 [==============================] - 0s 91ms/step - loss: 0.0372 - mean_absolute_error: 0.1081 - val_loss: 0.07
85 - val_mean_absolute_error: 0.2093
Epoch 454/1000
1/1 [==============================] - 0s 91ms/step - loss: 0.0372 - mean_absolute_error: 0.1076 - val_loss: 0.07
77 - val_mean_absolute_error: 0.2065
Epoch 455/1000
1/1 [==============================] - 0s 91ms/step - loss: 0.0371 - mean_absolute_error: 0.1073 - val_loss: 0.07
74 - val_mean_absolute_error: 0.2052
Epoch 456/1000
1/1 [==============================] - 0s 91ms/step - loss: 0.0371 - mean_absolute_error: 0.1075 - val_loss: 0.07
79 - val_mean_absolute_error: 0.2076
Epoch 457/1000
1/1 [==============================] - 0s 91ms/step - loss: 0.0372 - mean_absolute_error: 0.1075 - val_loss: 0.07
72 - val_mean_absolute_error: 0.2042
Epoch 458/1000
1/1 [==============================] - 0s 92ms/step - loss: 0.0371 - mean_absolute_error: 0.1076 - val_loss: 0.07
76 - val_mean_absolute_error: 0.2065
Epoch 459/1000
1/1 [==============================] - 0s 91ms/step - loss: 0.0372 - mean_absolute_error: 0.1071 - val_loss: 0.07
71 - val_mean_absolute_error: 0.2042
Epoch 460/1000
1/1 [==============================] - 0s 96ms/step - loss: 0.0372 - mean_absolute_error: 0.1075 - val_loss: 0.07
71 - val_mean_absolute_error: 0.2051
Epoch 461/1000
1/1 [==============================] - 0s 95ms/step - loss: 0.0371 - mean_absolute_error: 0.1071 - val_loss: 0.07
69 - val_mean_absolute_error: 0.2035
Epoch 462/1000
1/1 [==============================] - 0s 95ms/step - loss: 0.0371 - mean_absolute_error: 0.1069 - val_loss: 0.07
71 - val_mean_absolute_error: 0.2051
Epoch 463/1000
1/1 [==============================] - 0s 94ms/step - loss: 0.0371 - mean_absolute_error: 0.1069 - val_loss: 0.07
69 - val_mean_absolute_error: 0.2045
Epoch 464/1000
1/1 [==============================] - 0s 101ms/step - loss: 0.0371 - mean_absolute_error: 0.1070 - val_loss: 0.0
761 - val_mean_absolute_error: 0.2025
Epoch 465/1000
1/1 [==============================] - 0s 95ms/step - loss: 0.0371 - mean_absolute_error: 0.1072 - val_loss: 0.07
61 - val_mean_absolute_error: 0.2030
Epoch 466/1000
1/1 [==============================] - 0s 94ms/step - loss: 0.0371 - mean_absolute_error: 0.1071 - val_loss: 0.07
56 - val_mean_absolute_error: 0.2013
Epoch 467/1000
1/1 [==============================] - 0s 92ms/step - loss: 0.0371 - mean_absolute_error: 0.1072 - val_loss: 0.07
59 - val_mean_absolute_error: 0.2030
Epoch 468/1000
1/1 [==============================] - 0s 93ms/step - loss: 0.0372 - mean_absolute_error: 0.1076 - val_loss: 0.07
51 - val_mean_absolute_error: 0.1998
Epoch 469/1000
1/1 [==============================] - 0s 93ms/step - loss: 0.0371 - mean_absolute_error: 0.1075 - val_loss: 0.07
54 - val_mean_absolute_error: 0.2021
Epoch 470/1000
1/1 [==============================] - 0s 93ms/step - loss: 0.0371 - mean_absolute_error: 0.1068 - val_loss: 0.07
54 - val_mean_absolute_error: 0.2024
Epoch 471/1000
1/1 [==============================] - 0s 94ms/step - loss: 0.0371 - mean_absolute_error: 0.1071 - val_loss: 0.07
56 - val_mean_absolute_error: 0.2029
Epoch 472/1000
1/1 [==============================] - 0s 93ms/step - loss: 0.0371 - mean_absolute_error: 0.1067 - val_loss: 0.07
51 - val_mean_absolute_error: 0.2005
Epoch 473/1000
1/1 [==============================] - 0s 93ms/step - loss: 0.0370 - mean_absolute_error: 0.1067 - val_loss: 0.07
50 - val_mean_absolute_error: 0.2013
Epoch 474/1000
1/1 [==============================] - 0s 93ms/step - loss: 0.0371 - mean_absolute_error: 0.1069 - val_loss: 0.07
49 - val_mean_absolute_error: 0.2012
Epoch 475/1000
1/1 [==============================] - 0s 93ms/step - loss: 0.0370 - mean_absolute_error: 0.1066 - val_loss: 0.07
47 - val_mean_absolute_error: 0.2000
Epoch 476/1000
1/1 [==============================] - 0s 94ms/step - loss: 0.0370 - mean_absolute_error: 0.1067 - val_loss: 0.07
47 - val_mean_absolute_error: 0.2005
Epoch 477/1000
1/1 [==============================] - 0s 109ms/step - loss: 0.0370 - mean_absolute_error: 0.1066 - val_loss: 0.0
```

```
741 - val_mean_absolute_error: 0.1986
Epoch 478/1000
1/1 [==============================] - 0s 92ms/step - loss: 0.0370 - mean_absolute_error: 0.1068 - val_loss: 0.07
40 - val_mean_absolute_error: 0.1996
Epoch 479/1000
1/1 [==============================] - 0s 92ms/step - loss: 0.0370 - mean_absolute_error: 0.1068 - val_loss: 0.07
41 - val_mean_absolute_error: 0.1991
Epoch 480/1000
1/1 [==============================] - 0s 93ms/step - loss: 0.0370 - mean_absolute_error: 0.1067 - val_loss: 0.07
39 - val_mean_absolute_error: 0.1988
Epoch 481/1000
1/1 [==============================] - 0s 95ms/step - loss: 0.0370 - mean_absolute_error: 0.1063 - val_loss: 0.07
35 - val_mean_absolute_error: 0.1986
Epoch 482/1000
1/1 [==============================] - 0s 92ms/step - loss: 0.0370 - mean_absolute_error: 0.1066 - val_loss: 0.07
37 - val_mean_absolute_error: 0.1994
Epoch 483/1000
1/1 [==============================] - 0s 94ms/step - loss: 0.0370 - mean_absolute_error: 0.1065 - val_loss: 0.07
36 - val_mean_absolute_error: 0.1981
Epoch 484/1000
1/1 [==============================] - 0s 92ms/step - loss: 0.0370 - mean_absolute_error: 0.1067 - val_loss: 0.07
41 - val_mean_absolute_error: 0.2007
Epoch 485/1000
1/1 [==============================] - 0s 92ms/step - loss: 0.0370 - mean_absolute_error: 0.1065 - val_loss: 0.07
33 - val_mean_absolute_error: 0.1984
Epoch 486/1000
1/1 [==============================] - 0s 91ms/step - loss: 0.0370 - mean_absolute_error: 0.1069 - val_loss: 0.07
35 - val_mean_absolute_error: 0.2005
Epoch 487/1000
1/1 [==============================] - 0s 92ms/step - loss: 0.0370 - mean_absolute_error: 0.1066 - val_loss: 0.07
26 - val_mean_absolute_error: 0.1967
Epoch 488/1000
1/1 [==============================] - 0s 92ms/step - loss: 0.0370 - mean_absolute_error: 0.1072 - val_loss: 0.07
32 - val_mean_absolute_error: 0.2000
Epoch 489/1000
1/1 [==============================] - 0s 92ms/step - loss: 0.0370 - mean_absolute_error: 0.1068 - val_loss: 0.07
21 - val_mean_absolute_error: 0.1962
Epoch 490/1000
1/1 [==============================] - 0s 92ms/step - loss: 0.0370 - mean_absolute_error: 0.1071 - val_loss: 0.07
30 - val_mean_absolute_error: 0.1999
Epoch 491/1000
1/1 [==============================] - 0s 92ms/step - loss: 0.0370 - mean_absolute_error: 0.1064 - val_loss: 0.07
21 - val_mean_absolute_error: 0.1963
Epoch 492/1000
1/1 [==============================] - 0s 91ms/step - loss: 0.0370 - mean_absolute_error: 0.1070 - val_loss: 0.07
28 - val_mean_absolute_error: 0.1999
Epoch 493/1000
1/1 [==============================] - 0s 91ms/step - loss: 0.0370 - mean_absolute_error: 0.1064 - val_loss: 0.07
19 - val_mean_absolute_error: 0.1961
Epoch 494/1000
1/1 [==============================] - 0s 92ms/step - loss: 0.0370 - mean_absolute_error: 0.1068 - val_loss: 0.07
26 - val_mean_absolute_error: 0.1991
Epoch 495/1000
1/1 [==============================] - 0s 92ms/step - loss: 0.0370 - mean_absolute_error: 0.1064 - val_loss: 0.07
18 - val_mean_absolute_error: 0.1967
Epoch 496/1000
1/1 [==============================] - 0s 92ms/step - loss: 0.0370 - mean_absolute_error: 0.1064 - val_loss: 0.07
21 - val_mean_absolute_error: 0.1976
Epoch 497/1000
1/1 [==============================] - 0s 91ms/step - loss: 0.0370 - mean_absolute_error: 0.1065 - val_loss: 0.07
20 - val_mean_absolute_error: 0.1979
Epoch 498/1000
1/1 [==============================] - 0s 91ms/step - loss: 0.0369 - mean_absolute_error: 0.1060 - val_loss: 0.07
18 - val_mean_absolute_error: 0.1959
Epoch 499/1000
1/1 [==============================] - 0s 92ms/step - loss: 0.0370 - mean_absolute_error: 0.1064 - val_loss: 0.07
23 - val_mean_absolute_error: 0.1978
Epoch 500/1000
1/1 [==============================] - 0s 92ms/step - loss: 0.0370 - mean_absolute_error: 0.1063 - val_loss: 0.07
12 - val_mean_absolute_error: 0.1953
Epoch 501/1000
1/1 [==============================] - 0s 92ms/step - loss: 0.0371 - mean_absolute_error: 0.1073 - val_loss: 0.07
19 - val_mean_absolute_error: 0.1986
Epoch 502/1000
1/1 [==============================] - 0s 92ms/step - loss: 0.0371 - mean_absolute_error: 0.1070 - val_loss: 0.07
10 - val_mean_absolute_error: 0.1967
Epoch 503/1000
1/1 [==============================] - 0s 92ms/step - loss: 0.0371 - mean_absolute_error: 0.1078 - val_loss: 0.07
18 - val_mean_absolute_error: 0.1977
Epoch 504/1000
1/1 [==============================] - 0s 97ms/step - loss: 0.0371 - mean_absolute_error: 0.1070 - val_loss: 0.07
11 - val_mean_absolute_error: 0.1960
Epoch 505/1000
```

```
1/1 [==============================] - 0s 93ms/step - loss: 0.0369 - mean_absolute_error: 0.1063 - val_loss: 0.07
10 - val_mean_absolute_error: 0.1962
Epoch 506/1000
1/1 [==============================] - 0s 92ms/step - loss: 0.0369 - mean_absolute_error: 0.1060 - val_loss: 0.07
10 - val_mean_absolute_error: 0.1954
Epoch 507/1000
1/1 [==============================] - 0s 92ms/step - loss: 0.0369 - mean_absolute_error: 0.1061 - val_loss: 0.07
11 - val_mean_absolute_error: 0.1967
Epoch 508/1000
1/1 [==============================] - 0s 92ms/step - loss: 0.0370 - mean_absolute_error: 0.1065 - val_loss: 0.07
18 - val_mean_absolute_error: 0.1978
Epoch 509/1000
1/1 [==============================] - 0s 92ms/step - loss: 0.0370 - mean_absolute_error: 0.1068 - val_loss: 0.07
08 - val_mean_absolute_error: 0.1960
Epoch 510/1000
1/1 [==============================] - 0s 92ms/step - loss: 0.0371 - mean_absolute_error: 0.1075 - val_loss: 0.07
17 - val_mean_absolute_error: 0.1977
Epoch 511/1000
1/1 [==============================] - 0s 93ms/step - loss: 0.0370 - mean_absolute_error: 0.1067 - val_loss: 0.07
07 - val_mean_absolute_error: 0.1970
Epoch 512/1000
1/1 [==============================] - 0s 93ms/step - loss: 0.0369 - mean_absolute_error: 0.1062 - val_loss: 0.07
09 - val_mean_absolute_error: 0.1953
Epoch 513/1000
1/1 [==============================] - 0s 93ms/step - loss: 0.0369 - mean_absolute_error: 0.1059 - val_loss: 0.07
14 - val_mean_absolute_error: 0.1974
Epoch 514/1000
1/1 [==============================] - 0s 92ms/step - loss: 0.0369 - mean_absolute_error: 0.1060 - val_loss: 0.07
08 - val_mean_absolute_error: 0.1974
Epoch 515/1000
1/1 [==============================] - 0s 92ms/step - loss: 0.0369 - mean_absolute_error: 0.1063 - val_loss: 0.07
13 - val_mean_absolute_error: 0.1963
Epoch 516/1000
1/1 [==============================] - 0s 92ms/step - loss: 0.0369 - mean_absolute_error: 0.1061 - val_loss: 0.07
12 - val_mean_absolute_error: 0.1981
Epoch 517/1000
1/1 [==============================] - 0s 92ms/step - loss: 0.0370 - mean_absolute_error: 0.1066 - val_loss: 0.07
07 - val_mean_absolute_error: 0.1953
Epoch 518/1000
1/1 [==============================] - 0s 92ms/step - loss: 0.0370 - mean_absolute_error: 0.1067 - val_loss: 0.07
09 - val_mean_absolute_error: 0.1969
Epoch 519/1000
1/1 [==============================] - 0s 91ms/step - loss: 0.0369 - mean_absolute_error: 0.1061 - val_loss: 0.07
12 - val_mean_absolute_error: 0.1972
Epoch 520/1000
1/1 [==============================] - 0s 91ms/step - loss: 0.0369 - mean_absolute_error: 0.1058 - val_loss: 0.07
05 - val_mean_absolute_error: 0.1953
Epoch 521/1000
1/1 [==============================] - 0s 92ms/step - loss: 0.0369 - mean_absolute_error: 0.1060 - val_loss: 0.07
12 - val_mean_absolute_error: 0.1971
Epoch 522/1000
1/1 [==============================] - 0s 93ms/step - loss: 0.0369 - mean_absolute_error: 0.1064 - val_loss: 0.07
15 - val_mean_absolute_error: 0.1966
Epoch 523/1000
1/1 [==============================] - 0s 91ms/step - loss: 0.0370 - mean_absolute_error: 0.1066 - val_loss: 0.07
06 - val_mean_absolute_error: 0.1961
Epoch 524/1000
1/1 [==============================] - 0s 93ms/step - loss: 0.0370 - mean_absolute_error: 0.1069 - val_loss: 0.07
19 - val_mean_absolute_error: 0.1976
Epoch 525/1000
1/1 [==============================] - 0s 92ms/step - loss: 0.0369 - mean_absolute_error: 0.1064 - val_loss: 0.07
07 - val_mean_absolute_error: 0.1963
Epoch 526/1000
1/1 [==============================] - 0s 92ms/step - loss: 0.0368 - mean_absolute_error: 0.1057 - val_loss: 0.07
06 - val_mean_absolute_error: 0.1953
Epoch 527/1000
1/1 [==============================] - 0s 95ms/step - loss: 0.0368 - mean_absolute_error: 0.1054 - val_loss: 0.07
13 - val_mean_absolute_error: 0.1964
Epoch 528/1000
1/1 [==============================] - 0s 92ms/step - loss: 0.0369 - mean_absolute_error: 0.1056 - val_loss: 0.07
07 - val_mean_absolute_error: 0.1957
Epoch 529/1000
1/1 [==============================] - 0s 91ms/step - loss: 0.0369 - mean_absolute_error: 0.1061 - val_loss: 0.07
13 - val_mean_absolute_error: 0.1960
Epoch 530/1000
1/1 [==============================] - 0s 92ms/step - loss: 0.0369 - mean_absolute_error: 0.1060 - val_loss: 0.07
11 - val_mean_absolute_error: 0.1967
Epoch 531/1000
1/1 [==============================] - 0s 91ms/step - loss: 0.0369 - mean_absolute_error: 0.1060 - val_loss: 0.07
02 - val_mean_absolute_error: 0.1945
Epoch 532/1000
1/1 [==============================] - 0s 91ms/step - loss: 0.0369 - mean_absolute_error: 0.1055 - val_loss: 0.07
12 - val_mean_absolute_error: 0.1968
```

```
Epoch 533/1000
1/1 [==============================] - 0s 90ms/step - loss: 0.0368 - mean_absolute_error: 0.1051 - val_loss: 0.07
08 - val_mean_absolute_error: 0.1959
Epoch 534/1000
1/1 [==============================] - 0s 91ms/step - loss: 0.0367 - mean_absolute_error: 0.1047 - val_loss: 0.07
04 - val_mean_absolute_error: 0.1949
Epoch 535/1000
1/1 [==============================] - 0s 91ms/step - loss: 0.0367 - mean_absolute_error: 0.1049 - val_loss: 0.07
14 - val_mean_absolute_error: 0.1979
Epoch 536/1000
1/1 [==============================] - 0s 92ms/step - loss: 0.0368 - mean_absolute_error: 0.1054 - val_loss: 0.07
09 - val_mean_absolute_error: 0.1963
Epoch 537/1000
1/1 [==============================] - 0s 93ms/step - loss: 0.0368 - mean_absolute_error: 0.1052 - val_loss: 0.07
14 - val_mean_absolute_error: 0.1982
Epoch 538/1000
1/1 [==============================] - 0s 93ms/step - loss: 0.0368 - mean_absolute_error: 0.1054 - val_loss: 0.07
09 - val_mean_absolute_error: 0.1971
Epoch 539/1000
1/1 [==============================] - 0s 93ms/step - loss: 0.0369 - mean_absolute_error: 0.1056 - val_loss: 0.07
13 - val_mean_absolute_error: 0.1986
Epoch 540/1000
1/1 [==============================] - 0s 92ms/step - loss: 0.0368 - mean_absolute_error: 0.1056 - val_loss: 0.07
16 - val_mean_absolute_error: 0.1983
Epoch 541/1000
1/1 [==============================] - 0s 91ms/step - loss: 0.0367 - mean_absolute_error: 0.1045 - val_loss: 0.07
14 - val_mean_absolute_error: 0.1990
Epoch 542/1000
1/1 [==============================] - 0s 94ms/step - loss: 0.0367 - mean_absolute_error: 0.1048 - val_loss: 0.07
13 - val_mean_absolute_error: 0.1975
Epoch 543/1000
1/1 [==============================] - 0s 93ms/step - loss: 0.0367 - mean_absolute_error: 0.1047 - val_loss: 0.07
12 - val_mean_absolute_error: 0.1978
Epoch 544/1000
1/1 [==============================] - 0s 93ms/step - loss: 0.0367 - mean_absolute_error: 0.1046 - val_loss: 0.07
26 - val_mean_absolute_error: 0.2021
Epoch 545/1000
1/1 [==============================] - 0s 91ms/step - loss: 0.0368 - mean_absolute_error: 0.1055 - val_loss: 0.07
14 - val_mean_absolute_error: 0.1979
Epoch 546/1000
1/1 [==============================] - 0s 92ms/step - loss: 0.0368 - mean_absolute_error: 0.1060 - val_loss: 0.07
17 - val_mean_absolute_error: 0.2005
Epoch 547/1000
1/1 [==============================] - 0s 92ms/step - loss: 0.0367 - mean_absolute_error: 0.1053 - val_loss: 0.07
17 - val_mean_absolute_error: 0.1988
Epoch 548/1000
1/1 [==============================] - 0s 93ms/step - loss: 0.0367 - mean_absolute_error: 0.1048 - val_loss: 0.07
14 - val_mean_absolute_error: 0.1978
Epoch 549/1000
1/1 [==============================] - 0s 98ms/step - loss: 0.0367 - mean_absolute_error: 0.1048 - val_loss: 0.07
23 - val_mean_absolute_error: 0.2010
Epoch 550/1000
1/1 [==============================] - 0s 92ms/step - loss: 0.0366 - mean_absolute_error: 0.1041 - val_loss: 0.07
23 - val_mean_absolute_error: 0.2006
Epoch 551/1000
1/1 [==============================] - 0s 92ms/step - loss: 0.0367 - mean_absolute_error: 0.1043 - val_loss: 0.07
16 - val_mean_absolute_error: 0.1991
Epoch 552/1000
1/1 [==============================] - 0s 92ms/step - loss: 0.0367 - mean_absolute_error: 0.1048 - val_loss: 0.07
18 - val_mean_absolute_error: 0.1996
Epoch 553/1000
1/1 [==============================] - 0s 92ms/step - loss: 0.0367 - mean_absolute_error: 0.1050 - val_loss: 0.07
21 - val_mean_absolute_error: 0.2012
Epoch 554/1000
1/1 [==============================] - 0s 93ms/step - loss: 0.0367 - mean_absolute_error: 0.1049 - val_loss: 0.07
13 - val_mean_absolute_error: 0.1980
Epoch 555/1000
1/1 [==============================] - 0s 92ms/step - loss: 0.0367 - mean_absolute_error: 0.1048 - val_loss: 0.07
25 - val_mean_absolute_error: 0.2017
Epoch 556/1000
1/1 [==============================] - 0s 91ms/step - loss: 0.0367 - mean_absolute_error: 0.1051 - val_loss: 0.07
10 - val_mean_absolute_error: 0.1974
Epoch 557/1000
1/1 [==============================] - 0s 92ms/step - loss: 0.0366 - mean_absolute_error: 0.1043 - val_loss: 0.07
15 - val_mean_absolute_error: 0.1988
Epoch 558/1000
1/1 [==============================] - 0s 91ms/step - loss: 0.0366 - mean_absolute_error: 0.1041 - val_loss: 0.07
23 - val_mean_absolute_error: 0.2008
Epoch 559/1000
1/1 [==============================] - 0s 91ms/step - loss: 0.0366 - mean_absolute_error: 0.1043 - val_loss: 0.07
11 - val_mean_absolute_error: 0.1972
Epoch 560/1000
1/1 [==============================] - 0s 93ms/step - loss: 0.0366 - mean_absolute_error: 0.1042 - val_loss: 0.07
```

```
18 - val_mean_absolute_error: 0.1997
Epoch 561/1000
1/1 [==============================] - 0s 91ms/step - loss: 0.0366 - mean_absolute_error: 0.1040 - val_loss: 0.07
23 - val_mean_absolute_error: 0.2007
Epoch 562/1000
1/1 [==============================] - 0s 92ms/step - loss: 0.0366 - mean_absolute_error: 0.1041 - val_loss: 0.07
11 - val_mean_absolute_error: 0.1978
Epoch 563/1000
1/1 [==============================] - 0s 91ms/step - loss: 0.0366 - mean_absolute_error: 0.1039 - val_loss: 0.07
22 - val_mean_absolute_error: 0.2000
Epoch 564/1000
1/1 [==============================] - 0s 91ms/step - loss: 0.0366 - mean_absolute_error: 0.1042 - val_loss: 0.07
16 - val_mean_absolute_error: 0.2001
Epoch 565/1000
1/1 [==============================] - 0s 91ms/step - loss: 0.0366 - mean_absolute_error: 0.1039 - val_loss: 0.07
16 - val_mean_absolute_error: 0.1988
Epoch 566/1000
1/1 [==============================] - 0s 90ms/step - loss: 0.0366 - mean_absolute_error: 0.1037 - val_loss: 0.07
26 - val_mean_absolute_error: 0.2015
Epoch 567/1000
1/1 [==============================] - 0s 91ms/step - loss: 0.0366 - mean_absolute_error: 0.1047 - val_loss: 0.07
11 - val_mean_absolute_error: 0.1973
Epoch 568/1000
1/1 [==============================] - 0s 91ms/step - loss: 0.0367 - mean_absolute_error: 0.1052 - val_loss: 0.07
34 - val_mean_absolute_error: 0.2038
Epoch 569/1000
1/1 [==============================] - 0s 91ms/step - loss: 0.0368 - mean_absolute_error: 0.1058 - val_loss: 0.07
21 - val_mean_absolute_error: 0.2000
Epoch 570/1000
1/1 [==============================] - 0s 92ms/step - loss: 0.0368 - mean_absolute_error: 0.1058 - val_loss: 0.07
35 - val_mean_absolute_error: 0.2051
Epoch 571/1000
1/1 [==============================] - 0s 91ms/step - loss: 0.0368 - mean_absolute_error: 0.1057 - val_loss: 0.07
35 - val_mean_absolute_error: 0.2022
Epoch 572/1000
1/1 [==============================] - 0s 91ms/step - loss: 0.0368 - mean_absolute_error: 0.1055 - val_loss: 0.07
33 - val_mean_absolute_error: 0.2053
Epoch 573/1000
1/1 [==============================] - 0s 92ms/step - loss: 0.0367 - mean_absolute_error: 0.1051 - val_loss: 0.07
36 - val_mean_absolute_error: 0.2028
Epoch 574/1000
1/1 [==============================] - 0s 91ms/step - loss: 0.0366 - mean_absolute_error: 0.1048 - val_loss: 0.07
38 - val_mean_absolute_error: 0.2041
Epoch 575/1000
1/1 [==============================] - 0s 92ms/step - loss: 0.0366 - mean_absolute_error: 0.1037 - val_loss: 0.07
39 - val_mean_absolute_error: 0.2049
Epoch 576/1000
1/1 [==============================] - 0s 93ms/step - loss: 0.0366 - mean_absolute_error: 0.1038 - val_loss: 0.07
42 - val_mean_absolute_error: 0.2039
Epoch 577/1000
1/1 [==============================] - 0s 95ms/step - loss: 0.0366 - mean_absolute_error: 0.1046 - val_loss: 0.07
43 - val_mean_absolute_error: 0.2071
Epoch 578/1000
1/1 [==============================] - 0s 93ms/step - loss: 0.0366 - mean_absolute_error: 0.1043 - val_loss: 0.07
39 - val_mean_absolute_error: 0.2038
Epoch 579/1000
1/1 [==============================] - 0s 94ms/step - loss: 0.0366 - mean_absolute_error: 0.1038 - val_loss: 0.07
55 - val_mean_absolute_error: 0.2084
Epoch 580/1000
1/1 [==============================] - 0s 92ms/step - loss: 0.0366 - mean_absolute_error: 0.1039 - val_loss: 0.07
45 - val_mean_absolute_error: 0.2068
Epoch 581/1000
1/1 [==============================] - 0s 93ms/step - loss: 0.0366 - mean_absolute_error: 0.1042 - val_loss: 0.07
59 - val_mean_absolute_error: 0.2101
Epoch 582/1000
1/1 [==============================] - 0s 94ms/step - loss: 0.0365 - mean_absolute_error: 0.1035 - val_loss: 0.07
60 - val_mean_absolute_error: 0.2101
Epoch 583/1000
1/1 [==============================] - 0s 92ms/step - loss: 0.0365 - mean_absolute_error: 0.1037 - val_loss: 0.07
57 - val_mean_absolute_error: 0.2106
Epoch 584/1000
1/1 [==============================] - 0s 93ms/step - loss: 0.0366 - mean_absolute_error: 0.1037 - val_loss: 0.07
68 - val_mean_absolute_error: 0.2123
Epoch 585/1000
1/1 [==============================] - 0s 92ms/step - loss: 0.0365 - mean_absolute_error: 0.1039 - val_loss: 0.07
61 - val_mean_absolute_error: 0.2096
Epoch 586/1000
1/1 [==============================] - 0s 92ms/step - loss: 0.0366 - mean_absolute_error: 0.1039 - val_loss: 0.07
68 - val_mean_absolute_error: 0.2136
Epoch 587/1000
1/1 [==============================] - 0s 93ms/step - loss: 0.0366 - mean_absolute_error: 0.1041 - val_loss: 0.07
69 - val_mean_absolute_error: 0.2105
Epoch 588/1000
```

```
1/1 [==============================] - 0s 92ms/step - loss: 0.0365 - mean_absolute_error: 0.1036 - val_loss: 0.07
61 - val_mean_absolute_error: 0.2106
Epoch 589/1000
1/1 [==============================] - 0s 92ms/step - loss: 0.0365 - mean_absolute_error: 0.1032 - val_loss: 0.07
67 - val_mean_absolute_error: 0.2121
Epoch 590/1000
1/1 [==============================] - 0s 92ms/step - loss: 0.0366 - mean_absolute_error: 0.1037 - val_loss: 0.07
66 - val_mean_absolute_error: 0.2112
Epoch 591/1000
1/1 [==============================] - 0s 92ms/step - loss: 0.0365 - mean_absolute_error: 0.1032 - val_loss: 0.07
68 - val_mean_absolute_error: 0.2117
Epoch 592/1000
1/1 [==============================] - 0s 93ms/step - loss: 0.0365 - mean_absolute_error: 0.1030 - val_loss: 0.07
71 - val_mean_absolute_error: 0.2131
Epoch 593/1000
1/1 [==============================] - 0s 93ms/step - loss: 0.0365 - mean_absolute_error: 0.1034 - val_loss: 0.07
69 - val_mean_absolute_error: 0.2133
Epoch 594/1000
1/1 [==============================] - 0s 92ms/step - loss: 0.0365 - mean_absolute_error: 0.1036 - val_loss: 0.07
73 - val_mean_absolute_error: 0.2127
Epoch 595/1000
1/1 [==============================] - 0s 92ms/step - loss: 0.0365 - mean_absolute_error: 0.1041 - val_loss: 0.07
72 - val_mean_absolute_error: 0.2146
Epoch 596/1000
1/1 [==============================] - 0s 92ms/step - loss: 0.0366 - mean_absolute_error: 0.1050 - val_loss: 0.07
63 - val_mean_absolute_error: 0.2089
Epoch 597/1000
1/1 [==============================] - 0s 92ms/step - loss: 0.0368 - mean_absolute_error: 0.1064 - val_loss: 0.07
80 - val_mean_absolute_error: 0.2175
Epoch 598/1000
1/1 [==============================] - 0s 92ms/step - loss: 0.0368 - mean_absolute_error: 0.1058 - val_loss: 0.07
67 - val_mean_absolute_error: 0.2125
Epoch 599/1000
1/1 [==============================] - 0s 93ms/step - loss: 0.0366 - mean_absolute_error: 0.1048 - val_loss: 0.07
75 - val_mean_absolute_error: 0.2137
Epoch 600/1000
1/1 [==============================] - 0s 92ms/step - loss: 0.0365 - mean_absolute_error: 0.1033 - val_loss: 0.07
82 - val_mean_absolute_error: 0.2160
Epoch 601/1000
1/1 [==============================] - 0s 92ms/step - loss: 0.0365 - mean_absolute_error: 0.1032 - val_loss: 0.07
78 - val_mean_absolute_error: 0.2159
Epoch 602/1000
1/1 [==============================] - 0s 91ms/step - loss: 0.0365 - mean_absolute_error: 0.1040 - val_loss: 0.07
91 - val_mean_absolute_error: 0.2181
Epoch 603/1000
1/1 [==============================] - 0s 92ms/step - loss: 0.0366 - mean_absolute_error: 0.1040 - val_loss: 0.07
78 - val_mean_absolute_error: 0.2149
Epoch 604/1000
1/1 [==============================] - 0s 91ms/step - loss: 0.0365 - mean_absolute_error: 0.1033 - val_loss: 0.07
78 - val_mean_absolute_error: 0.2153
Epoch 605/1000
1/1 [==============================] - 0s 93ms/step - loss: 0.0365 - mean_absolute_error: 0.1036 - val_loss: 0.07
93 - val_mean_absolute_error: 0.2172
Epoch 606/1000
1/1 [==============================] - 0s 92ms/step - loss: 0.0365 - mean_absolute_error: 0.1036 - val_loss: 0.07
74 - val_mean_absolute_error: 0.2142
Epoch 607/1000
1/1 [==============================] - 0s 92ms/step - loss: 0.0365 - mean_absolute_error: 0.1034 - val_loss: 0.07
76 - val_mean_absolute_error: 0.2141
Epoch 608/1000
1/1 [==============================] - 0s 91ms/step - loss: 0.0365 - mean_absolute_error: 0.1040 - val_loss: 0.07
72 - val_mean_absolute_error: 0.2128
Epoch 609/1000
1/1 [==============================] - 0s 92ms/step - loss: 0.0364 - mean_absolute_error: 0.1031 - val_loss: 0.07
66 - val_mean_absolute_error: 0.2115
Epoch 610/1000
1/1 [==============================] - 0s 92ms/step - loss: 0.0364 - mean_absolute_error: 0.1028 - val_loss: 0.07
76 - val_mean_absolute_error: 0.2120
Epoch 611/1000
1/1 [==============================] - 0s 91ms/step - loss: 0.0365 - mean_absolute_error: 0.1039 - val_loss: 0.07
59 - val_mean_absolute_error: 0.2101
Epoch 612/1000
1/1 [==============================] - 0s 92ms/step - loss: 0.0365 - mean_absolute_error: 0.1038 - val_loss: 0.07
67 - val_mean_absolute_error: 0.2114
Epoch 613/1000
1/1 [==============================] - 0s 93ms/step - loss: 0.0365 - mean_absolute_error: 0.1033 - val_loss: 0.07
65 - val_mean_absolute_error: 0.2110
Epoch 614/1000
1/1 [==============================] - 0s 92ms/step - loss: 0.0365 - mean_absolute_error: 0.1035 - val_loss: 0.07
58 - val_mean_absolute_error: 0.2109
Epoch 615/1000
1/1 [==============================] - 0s 92ms/step - loss: 0.0365 - mean_absolute_error: 0.1035 - val_loss: 0.07
70 - val_mean_absolute_error: 0.2108
```

```
Epoch 616/1000
1/1 [==============================] - 0s 92ms/step - loss: 0.0365 - mean_absolute_error: 0.1044 - val_loss: 0.07
49 - val_mean_absolute_error: 0.2068
Epoch 617/1000
1/1 [==============================] - 0s 94ms/step - loss: 0.0365 - mean_absolute_error: 0.1037 - val_loss: 0.07
69 - val_mean_absolute_error: 0.2110
Epoch 618/1000
1/1 [==============================] - 0s 92ms/step - loss: 0.0364 - mean_absolute_error: 0.1029 - val_loss: 0.07
71 - val_mean_absolute_error: 0.2122
Epoch 619/1000
1/1 [==============================] - 0s 93ms/step - loss: 0.0365 - mean_absolute_error: 0.1035 - val_loss: 0.07
57 - val_mean_absolute_error: 0.2097
Epoch 620/1000
1/1 [==============================] - 0s 94ms/step - loss: 0.0365 - mean_absolute_error: 0.1042 - val_loss: 0.07
65 - val_mean_absolute_error: 0.2110
Epoch 621/1000
1/1 [==============================] - 0s 93ms/step - loss: 0.0365 - mean_absolute_error: 0.1038 - val_loss: 0.07
65 - val_mean_absolute_error: 0.2107
Epoch 622/1000
1/1 [==============================] - 0s 92ms/step - loss: 0.0364 - mean_absolute_error: 0.1037 - val_loss: 0.07
59 - val_mean_absolute_error: 0.2092
Epoch 623/1000
1/1 [==============================] - 0s 92ms/step - loss: 0.0364 - mean_absolute_error: 0.1031 - val_loss: 0.07
62 - val_mean_absolute_error: 0.2086
Epoch 624/1000
1/1 [==============================] - 0s 91ms/step - loss: 0.0364 - mean_absolute_error: 0.1034 - val_loss: 0.07
51 - val_mean_absolute_error: 0.2064
Epoch 625/1000
1/1 [==============================] - 0s 92ms/step - loss: 0.0365 - mean_absolute_error: 0.1034 - val_loss: 0.07
64 - val_mean_absolute_error: 0.2084
Epoch 626/1000
1/1 [==============================] - 0s 92ms/step - loss: 0.0364 - mean_absolute_error: 0.1031 - val_loss: 0.07
50 - val_mean_absolute_error: 0.2055
Epoch 627/1000
1/1 [==============================] - 0s 92ms/step - loss: 0.0364 - mean_absolute_error: 0.1028 - val_loss: 0.07
54 - val_mean_absolute_error: 0.2065
Epoch 628/1000
1/1 [==============================] - 0s 92ms/step - loss: 0.0364 - mean_absolute_error: 0.1029 - val_loss: 0.07
68 - val_mean_absolute_error: 0.2087
Epoch 629/1000
1/1 [==============================] - 0s 93ms/step - loss: 0.0364 - mean_absolute_error: 0.1035 - val_loss: 0.07
56 - val_mean_absolute_error: 0.2055
Epoch 630/1000
1/1 [==============================] - 0s 92ms/step - loss: 0.0364 - mean_absolute_error: 0.1035 - val_loss: 0.07
59 - val_mean_absolute_error: 0.2076
Epoch 631/1000
1/1 [==============================] - 0s 91ms/step - loss: 0.0364 - mean_absolute_error: 0.1028 - val_loss: 0.07
55 - val_mean_absolute_error: 0.2060
Epoch 632/1000
1/1 [==============================] - 0s 92ms/step - loss: 0.0363 - mean_absolute_error: 0.1022 - val_loss: 0.07
49 - val_mean_absolute_error: 0.2053
Epoch 633/1000
1/1 [==============================] - 0s 91ms/step - loss: 0.0364 - mean_absolute_error: 0.1027 - val_loss: 0.07
67 - val_mean_absolute_error: 0.2076
Epoch 634/1000
1/1 [==============================] - 0s 91ms/step - loss: 0.0364 - mean_absolute_error: 0.1028 - val_loss: 0.07
46 - val_mean_absolute_error: 0.2040
Epoch 635/1000
1/1 [==============================] - 0s 91ms/step - loss: 0.0364 - mean_absolute_error: 0.1028 - val_loss: 0.07
56 - val_mean_absolute_error: 0.2057
Epoch 636/1000
1/1 [==============================] - 0s 91ms/step - loss: 0.0363 - mean_absolute_error: 0.1023 - val_loss: 0.07
59 - val_mean_absolute_error: 0.2068
Epoch 637/1000
1/1 [==============================] - 0s 91ms/step - loss: 0.0363 - mean_absolute_error: 0.1022 - val_loss: 0.07
57 - val_mean_absolute_error: 0.2059
Epoch 638/1000
1/1 [==============================] - 0s 91ms/step - loss: 0.0364 - mean_absolute_error: 0.1022 - val_loss: 0.07
55 - val_mean_absolute_error: 0.2065
Epoch 639/1000
1/1 [==============================] - 0s 91ms/step - loss: 0.0364 - mean_absolute_error: 0.1027 - val_loss: 0.07
59 - val_mean_absolute_error: 0.2061
Epoch 640/1000
1/1 [==============================] - 0s 94ms/step - loss: 0.0363 - mean_absolute_error: 0.1025 - val_loss: 0.07
59 - val_mean_absolute_error: 0.2066
Epoch 641/1000
1/1 [==============================] - 0s 93ms/step - loss: 0.0363 - mean_absolute_error: 0.1025 - val_loss: 0.07
60 - val_mean_absolute_error: 0.2069
Epoch 642/1000
1/1 [==============================] - 0s 91ms/step - loss: 0.0363 - mean_absolute_error: 0.1022 - val_loss: 0.07
55 - val_mean_absolute_error: 0.2054
Epoch 643/1000
1/1 [==============================] - 0s 92ms/step - loss: 0.0363 - mean_absolute_error: 0.1023 - val_loss: 0.07
```

```
65 - val_mean_absolute_error: 0.2081
Epoch 644/1000
1/1 [==============================] - 0s 97ms/step - loss: 0.0363 - mean_absolute_error: 0.1026 - val_loss: 0.07
61 - val_mean_absolute_error: 0.2069
Epoch 645/1000
1/1 [==============================] - 0s 92ms/step - loss: 0.0363 - mean_absolute_error: 0.1029 - val_loss: 0.07
55 - val_mean_absolute_error: 0.2063
Epoch 646/1000
1/1 [==============================] - 0s 92ms/step - loss: 0.0363 - mean_absolute_error: 0.1023 - val_loss: 0.07
56 - val_mean_absolute_error: 0.2063
Epoch 647/1000
1/1 [==============================] - 0s 92ms/step - loss: 0.0363 - mean_absolute_error: 0.1019 - val_loss: 0.07
54 - val_mean_absolute_error: 0.2061
Epoch 648/1000
1/1 [==============================] - 0s 92ms/step - loss: 0.0363 - mean_absolute_error: 0.1026 - val_loss: 0.07
67 - val_mean_absolute_error: 0.2069
Epoch 649/1000
1/1 [==============================] - 0s 102ms/step - loss: 0.0363 - mean_absolute_error: 0.1021 - val_loss: 0.0
755 - val_mean_absolute_error: 0.2062
Epoch 650/1000
1/1 [==============================] - 0s 92ms/step - loss: 0.0363 - mean_absolute_error: 0.1026 - val_loss: 0.07
62 - val_mean_absolute_error: 0.2069
Epoch 651/1000
1/1 [==============================] - 0s 92ms/step - loss: 0.0363 - mean_absolute_error: 0.1024 - val_loss: 0.07
55 - val_mean_absolute_error: 0.2056
Epoch 652/1000
1/1 [==============================] - 0s 92ms/step - loss: 0.0362 - mean_absolute_error: 0.1019 - val_loss: 0.07
49 - val_mean_absolute_error: 0.2042
Epoch 653/1000
1/1 [==============================] - 0s 92ms/step - loss: 0.0363 - mean_absolute_error: 0.1021 - val_loss: 0.07
55 - val_mean_absolute_error: 0.2052
Epoch 654/1000
1/1 [==============================] - 0s 92ms/step - loss: 0.0362 - mean_absolute_error: 0.1018 - val_loss: 0.07
58 - val_mean_absolute_error: 0.2059
Epoch 655/1000
1/1 [==============================] - 0s 92ms/step - loss: 0.0362 - mean_absolute_error: 0.1019 - val_loss: 0.07
55 - val_mean_absolute_error: 0.2060
Epoch 656/1000
1/1 [==============================] - 0s 91ms/step - loss: 0.0363 - mean_absolute_error: 0.1022 - val_loss: 0.07
64 - val_mean_absolute_error: 0.2063
Epoch 657/1000
1/1 [==============================] - 0s 91ms/step - loss: 0.0363 - mean_absolute_error: 0.1024 - val_loss: 0.07
52 - val_mean_absolute_error: 0.2049
Epoch 658/1000
1/1 [==============================] - 0s 91ms/step - loss: 0.0363 - mean_absolute_error: 0.1026 - val_loss: 0.07
65 - val_mean_absolute_error: 0.2068
Epoch 659/1000
1/1 [==============================] - 0s 91ms/step - loss: 0.0363 - mean_absolute_error: 0.1024 - val_loss: 0.07
56 - val_mean_absolute_error: 0.2065
Epoch 660/1000
1/1 [==============================] - 0s 98ms/step - loss: 0.0363 - mean_absolute_error: 0.1027 - val_loss: 0.07
63 - val_mean_absolute_error: 0.2057
Epoch 661/1000
1/1 [==============================] - 0s 94ms/step - loss: 0.0363 - mean_absolute_error: 0.1024 - val_loss: 0.07
63 - val_mean_absolute_error: 0.2077
Epoch 662/1000
1/1 [==============================] - 0s 92ms/step - loss: 0.0363 - mean_absolute_error: 0.1025 - val_loss: 0.07
64 - val_mean_absolute_error: 0.2062
Epoch 663/1000
1/1 [==============================] - 0s 93ms/step - loss: 0.0363 - mean_absolute_error: 0.1028 - val_loss: 0.07
67 - val_mean_absolute_error: 0.2080
Epoch 664/1000
1/1 [==============================] - 0s 91ms/step - loss: 0.0363 - mean_absolute_error: 0.1024 - val_loss: 0.07
70 - val_mean_absolute_error: 0.2079
Epoch 665/1000
1/1 [==============================] - 0s 91ms/step - loss: 0.0362 - mean_absolute_error: 0.1018 - val_loss: 0.07
64 - val_mean_absolute_error: 0.2062
Epoch 666/1000
1/1 [==============================] - 0s 91ms/step - loss: 0.0363 - mean_absolute_error: 0.1021 - val_loss: 0.07
66 - val_mean_absolute_error: 0.2077
Epoch 667/1000
1/1 [==============================] - 0s 91ms/step - loss: 0.0362 - mean_absolute_error: 0.1018 - val_loss: 0.07
65 - val_mean_absolute_error: 0.2066
Epoch 668/1000
1/1 [==============================] - 0s 91ms/step - loss: 0.0363 - mean_absolute_error: 0.1024 - val_loss: 0.07
65 - val_mean_absolute_error: 0.2069
Epoch 669/1000
1/1 [==============================] - 0s 91ms/step - loss: 0.0363 - mean_absolute_error: 0.1030 - val_loss: 0.07
63 - val_mean_absolute_error: 0.2061
Epoch 670/1000
1/1 [==============================] - 0s 93ms/step - loss: 0.0363 - mean_absolute_error: 0.1019 - val_loss: 0.07
62 - val_mean_absolute_error: 0.2079
Epoch 671/1000
```

```
1/1 [==============================] - 0s 92ms/step - loss: 0.0363 - mean_absolute_error: 0.1019 - val_loss: 0.07
74 - val_mean_absolute_error: 0.2082
Epoch 672/1000
1/1 [==============================] - 0s 92ms/step - loss: 0.0363 - mean_absolute_error: 0.1019 - val_loss: 0.07
59 - val_mean_absolute_error: 0.2059
Epoch 673/1000
1/1 [==============================] - 0s 92ms/step - loss: 0.0363 - mean_absolute_error: 0.1021 - val_loss: 0.07
79 - val_mean_absolute_error: 0.2090
Epoch 674/1000
1/1 [==============================] - 0s 91ms/step - loss: 0.0363 - mean_absolute_error: 0.1022 - val_loss: 0.07
66 - val_mean_absolute_error: 0.2067
Epoch 675/1000
1/1 [==============================] - 0s 92ms/step - loss: 0.0363 - mean_absolute_error: 0.1027 - val_loss: 0.07
70 - val_mean_absolute_error: 0.2080
Epoch 676/1000
1/1 [==============================] - 0s 92ms/step - loss: 0.0362 - mean_absolute_error: 0.1018 - val_loss: 0.07
73 - val_mean_absolute_error: 0.2092
Epoch 677/1000
1/1 [==============================] - 0s 92ms/step - loss: 0.0362 - mean_absolute_error: 0.1017 - val_loss: 0.07
62 - val_mean_absolute_error: 0.2057
Epoch 678/1000
1/1 [==============================] - 0s 92ms/step - loss: 0.0362 - mean_absolute_error: 0.1019 - val_loss: 0.07
79 - val_mean_absolute_error: 0.2100
Epoch 679/1000
1/1 [==============================] - 0s 92ms/step - loss: 0.0362 - mean_absolute_error: 0.1015 - val_loss: 0.07
77 - val_mean_absolute_error: 0.2095
Epoch 680/1000
1/1 [==============================] - 0s 92ms/step - loss: 0.0362 - mean_absolute_error: 0.1012 - val_loss: 0.07
75 - val_mean_absolute_error: 0.2085
Epoch 681/1000
1/1 [==============================] - 0s 91ms/step - loss: 0.0362 - mean_absolute_error: 0.1015 - val_loss: 0.07
86 - val_mean_absolute_error: 0.2115
Epoch 682/1000
1/1 [==============================] - 0s 91ms/step - loss: 0.0362 - mean_absolute_error: 0.1013 - val_loss: 0.07
83 - val_mean_absolute_error: 0.2102
Epoch 683/1000
1/1 [==============================] - 0s 91ms/step - loss: 0.0362 - mean_absolute_error: 0.1015 - val_loss: 0.07
84 - val_mean_absolute_error: 0.2112
Epoch 684/1000
1/1 [==============================] - 0s 91ms/step - loss: 0.0362 - mean_absolute_error: 0.1022 - val_loss: 0.07
83 - val_mean_absolute_error: 0.2098
Epoch 685/1000
1/1 [==============================] - 0s 92ms/step - loss: 0.0362 - mean_absolute_error: 0.1019 - val_loss: 0.07
75 - val_mean_absolute_error: 0.2096
Epoch 686/1000
1/1 [==============================] - 0s 93ms/step - loss: 0.0363 - mean_absolute_error: 0.1026 - val_loss: 0.07
95 - val_mean_absolute_error: 0.2107
Epoch 687/1000
1/1 [==============================] - 0s 92ms/step - loss: 0.0363 - mean_absolute_error: 0.1024 - val_loss: 0.07
61 - val_mean_absolute_error: 0.2053
Epoch 688/1000
1/1 [==============================] - 0s 92ms/step - loss: 0.0364 - mean_absolute_error: 0.1033 - val_loss: 0.07
87 - val_mean_absolute_error: 0.2100
Epoch 689/1000
1/1 [==============================] - 0s 92ms/step - loss: 0.0363 - mean_absolute_error: 0.1028 - val_loss: 0.07
71 - val_mean_absolute_error: 0.2075
Epoch 690/1000
1/1 [==============================] - 0s 93ms/step - loss: 0.0362 - mean_absolute_error: 0.1015 - val_loss: 0.07
66 - val_mean_absolute_error: 0.2055
Epoch 691/1000
1/1 [==============================] - 0s 93ms/step - loss: 0.0362 - mean_absolute_error: 0.1012 - val_loss: 0.07
77 - val_mean_absolute_error: 0.2079
Epoch 692/1000
1/1 [==============================] - 0s 92ms/step - loss: 0.0362 - mean_absolute_error: 0.1015 - val_loss: 0.07
67 - val_mean_absolute_error: 0.2057
Epoch 693/1000
1/1 [==============================] - 0s 93ms/step - loss: 0.0362 - mean_absolute_error: 0.1019 - val_loss: 0.07
85 - val_mean_absolute_error: 0.2091
Epoch 694/1000
1/1 [==============================] - 0s 95ms/step - loss: 0.0362 - mean_absolute_error: 0.1012 - val_loss: 0.07
78 - val_mean_absolute_error: 0.2082
Epoch 695/1000
1/1 [==============================] - 0s 95ms/step - loss: 0.0361 - mean_absolute_error: 0.1011 - val_loss: 0.07
74 - val_mean_absolute_error: 0.2070
Epoch 696/1000
1/1 [==============================] - 0s 93ms/step - loss: 0.0361 - mean_absolute_error: 0.1009 - val_loss: 0.07
88 - val_mean_absolute_error: 0.2093
Epoch 697/1000
1/1 [==============================] - 0s 92ms/step - loss: 0.0362 - mean_absolute_error: 0.1012 - val_loss: 0.07
68 - val_mean_absolute_error: 0.2057
Epoch 698/1000
1/1 [==============================] - 0s 92ms/step - loss: 0.0362 - mean_absolute_error: 0.1016 - val_loss: 0.07
85 - val_mean_absolute_error: 0.2089
```

```
Epoch 699/1000
1/1 [==============================] - 0s 92ms/step - loss: 0.0361 - mean_absolute_error: 0.1012 - val_loss: 0.07
73 - val_mean_absolute_error: 0.2076
Epoch 700/1000
1/1 [==============================] - 0s 91ms/step - loss: 0.0361 - mean_absolute_error: 0.1015 - val_loss: 0.07
81 - val_mean_absolute_error: 0.2081
Epoch 701/1000
1/1 [==============================] - 0s 92ms/step - loss: 0.0362 - mean_absolute_error: 0.1016 - val_loss: 0.07
87 - val_mean_absolute_error: 0.2104
Epoch 702/1000
1/1 [==============================] - 0s 92ms/step - loss: 0.0362 - mean_absolute_error: 0.1023 - val_loss: 0.07
76 - val_mean_absolute_error: 0.2066
Epoch 703/1000
1/1 [==============================] - 0s 95ms/step - loss: 0.0363 - mean_absolute_error: 0.1031 - val_loss: 0.07
92 - val_mean_absolute_error: 0.2113
Epoch 704/1000
1/1 [==============================] - 0s 93ms/step - loss: 0.0364 - mean_absolute_error: 0.1032 - val_loss: 0.07
93 - val_mean_absolute_error: 0.2102
Epoch 705/1000
1/1 [==============================] - 0s 92ms/step - loss: 0.0363 - mean_absolute_error: 0.1035 - val_loss: 0.07
80 - val_mean_absolute_error: 0.2091
Epoch 706/1000
1/1 [==============================] - 0s 92ms/step - loss: 0.0361 - mean_absolute_error: 0.1017 - val_loss: 0.07
97 - val_mean_absolute_error: 0.2114
Epoch 707/1000
1/1 [==============================] - 0s 92ms/step - loss: 0.0360 - mean_absolute_error: 0.1015 - val_loss: 0.07
89 - val_mean_absolute_error: 0.2122
Epoch 708/1000
1/1 [==============================] - 0s 92ms/step - loss: 0.0360 - mean_absolute_error: 0.1023 - val_loss: 0.08
29 - val_mean_absolute_error: 0.2165
Epoch 709/1000
1/1 [==============================] - 0s 92ms/step - loss: 0.0360 - mean_absolute_error: 0.1029 - val_loss: 0.07
97 - val_mean_absolute_error: 0.2142
Epoch 710/1000
1/1 [==============================] - 0s 96ms/step - loss: 0.0355 - mean_absolute_error: 0.1036 - val_loss: 0.08
22 - val_mean_absolute_error: 0.2163
Epoch 711/1000
1/1 [==============================] - 0s 91ms/step - loss: 0.0350 - mean_absolute_error: 0.1042 - val_loss: 0.07
71 - val_mean_absolute_error: 0.2109
Epoch 712/1000
1/1 [==============================] - 0s 92ms/step - loss: 0.0344 - mean_absolute_error: 0.1055 - val_loss: 0.08
35 - val_mean_absolute_error: 0.2171
Epoch 713/1000
1/1 [==============================] - 0s 93ms/step - loss: 0.0337 - mean_absolute_error: 0.1073 - val_loss: 0.07
68 - val_mean_absolute_error: 0.2192
Epoch 714/1000
1/1 [==============================] - 0s 92ms/step - loss: 0.0328 - mean_absolute_error: 0.1061 - val_loss: 0.07
55 - val_mean_absolute_error: 0.2173
Epoch 715/1000
1/1 [==============================] - 0s 93ms/step - loss: 0.0324 - mean_absolute_error: 0.1100 - val_loss: 0.07
01 - val_mean_absolute_error: 0.2092
Epoch 716/1000
1/1 [==============================] - 0s 93ms/step - loss: 0.0323 - mean_absolute_error: 0.1127 - val_loss: 0.06
14 - val_mean_absolute_error: 0.1997
Epoch 717/1000
1/1 [==============================] - 0s 93ms/step - loss: 0.0292 - mean_absolute_error: 0.1187 - val_loss: 0.05
96 - val_mean_absolute_error: 0.1981
Epoch 718/1000
1/1 [==============================] - 0s 95ms/step - loss: 0.0251 - mean_absolute_error: 0.1163 - val_loss: 0.05
06 - val_mean_absolute_error: 0.1841
Epoch 719/1000
1/1 [==============================] - 0s 92ms/step - loss: 0.0228 - mean_absolute_error: 0.1092 - val_loss: 0.04
68 - val_mean_absolute_error: 0.1789
Epoch 720/1000
1/1 [==============================] - 0s 92ms/step - loss: 0.0220 - mean_absolute_error: 0.1113 - val_loss: 0.04
82 - val_mean_absolute_error: 0.1734
Epoch 721/1000
1/1 [==============================] - 0s 93ms/step - loss: 0.0207 - mean_absolute_error: 0.1077 - val_loss: 0.04
22 - val_mean_absolute_error: 0.1687
Epoch 722/1000
1/1 [==============================] - 0s 92ms/step - loss: 0.0192 - mean_absolute_error: 0.1039 - val_loss: 0.04
32 - val_mean_absolute_error: 0.1651
Epoch 723/1000
1/1 [==============================] - 0s 92ms/step - loss: 0.0178 - mean_absolute_error: 0.0990 - val_loss: 0.03
49 - val_mean_absolute_error: 0.1441
Epoch 724/1000
1/1 [==============================] - 0s 92ms/step - loss: 0.0152 - mean_absolute_error: 0.0923 - val_loss: 0.03
54 - val_mean_absolute_error: 0.1475
Epoch 725/1000
1/1 [==============================] - 0s 92ms/step - loss: 0.0166 - mean_absolute_error: 0.1018 - val_loss: 0.03
68 - val_mean_absolute_error: 0.1568
Epoch 726/1000
1/1 [==============================] - 0s 91ms/step - loss: 0.0171 - mean_absolute_error: 0.1036 - val_loss: 0.03
```

```
13 - val_mean_absolute_error: 0.1415
Epoch 727/1000
1/1 [==============================] - 0s 92ms/step - loss: 0.0147 - mean_absolute_error: 0.0954 - val_loss: 0.02
91 - val_mean_absolute_error: 0.1371
Epoch 728/1000
1/1 [==============================] - 0s 92ms/step - loss: 0.0147 - mean_absolute_error: 0.0973 - val_loss: 0.02
99 - val_mean_absolute_error: 0.1398
Epoch 729/1000
1/1 [==============================] - 0s 92ms/step - loss: 0.0138 - mean_absolute_error: 0.0944 - val_loss: 0.03
16 - val_mean_absolute_error: 0.1454
Epoch 730/1000
1/1 [==============================] - 0s 92ms/step - loss: 0.0135 - mean_absolute_error: 0.0903 - val_loss: 0.03
13 - val_mean_absolute_error: 0.1437
Epoch 731/1000
1/1 [==============================] - 0s 92ms/step - loss: 0.0129 - mean_absolute_error: 0.0905 - val_loss: 0.03
34 - val_mean_absolute_error: 0.1423
Epoch 732/1000
1/1 [==============================] - 0s 92ms/step - loss: 0.0129 - mean_absolute_error: 0.0896 - val_loss: 0.03
08 - val_mean_absolute_error: 0.1349
Epoch 733/1000
1/1 [==============================] - 0s 92ms/step - loss: 0.0126 - mean_absolute_error: 0.0894 - val_loss: 0.03
51 - val_mean_absolute_error: 0.1429
Epoch 734/1000
1/1 [==============================] - 0s 92ms/step - loss: 0.0123 - mean_absolute_error: 0.0882 - val_loss: 0.03
15 - val_mean_absolute_error: 0.1377
Epoch 735/1000
1/1 [==============================] - 0s 91ms/step - loss: 0.0114 - mean_absolute_error: 0.0845 - val_loss: 0.03
21 - val_mean_absolute_error: 0.1382
Epoch 736/1000
1/1 [==============================] - 0s 92ms/step - loss: 0.0112 - mean_absolute_error: 0.0835 - val_loss: 0.03
55 - val_mean_absolute_error: 0.1430
Epoch 737/1000
1/1 [==============================] - 0s 92ms/step - loss: 0.0109 - mean_absolute_error: 0.0817 - val_loss: 0.03
47 - val_mean_absolute_error: 0.1393
Epoch 738/1000
1/1 [==============================] - 0s 92ms/step - loss: 0.0109 - mean_absolute_error: 0.0836 - val_loss: 0.03
44 - val_mean_absolute_error: 0.1420
Epoch 739/1000
1/1 [==============================] - 0s 91ms/step - loss: 0.0103 - mean_absolute_error: 0.0796 - val_loss: 0.03
57 - val_mean_absolute_error: 0.1423
Epoch 740/1000
1/1 [==============================] - 0s 92ms/step - loss: 0.0095 - mean_absolute_error: 0.0761 - val_loss: 0.03
45 - val_mean_absolute_error: 0.1395
Epoch 741/1000
1/1 [==============================] - 0s 92ms/step - loss: 0.0097 - mean_absolute_error: 0.0774 - val_loss: 0.03
53 - val_mean_absolute_error: 0.1418
Epoch 742/1000
1/1 [==============================] - 0s 91ms/step - loss: 0.0095 - mean_absolute_error: 0.0761 - val_loss: 0.03
79 - val_mean_absolute_error: 0.1405
Epoch 743/1000
1/1 [==============================] - 0s 91ms/step - loss: 0.0089 - mean_absolute_error: 0.0734 - val_loss: 0.03
63 - val_mean_absolute_error: 0.1410
Epoch 744/1000
1/1 [==============================] - 0s 91ms/step - loss: 0.0093 - mean_absolute_error: 0.0766 - val_loss: 0.03
99 - val_mean_absolute_error: 0.1503
Epoch 745/1000
1/1 [==============================] - 0s 91ms/step - loss: 0.0091 - mean_absolute_error: 0.0754 - val_loss: 0.03
76 - val_mean_absolute_error: 0.1398
Epoch 746/1000
1/1 [==============================] - 0s 91ms/step - loss: 0.0093 - mean_absolute_error: 0.0748 - val_loss: 0.03
62 - val_mean_absolute_error: 0.1450
Epoch 747/1000
1/1 [==============================] - 0s 93ms/step - loss: 0.0094 - mean_absolute_error: 0.0765 - val_loss: 0.04
40 - val_mean_absolute_error: 0.1529
Epoch 748/1000
1/1 [==============================] - 0s 92ms/step - loss: 0.0100 - mean_absolute_error: 0.0779 - val_loss: 0.03
86 - val_mean_absolute_error: 0.1445
Epoch 749/1000
1/1 [==============================] - 0s 92ms/step - loss: 0.0088 - mean_absolute_error: 0.0742 - val_loss: 0.03
75 - val_mean_absolute_error: 0.1412
Epoch 750/1000
1/1 [==============================] - 0s 93ms/step - loss: 0.0086 - mean_absolute_error: 0.0733 - val_loss: 0.03
99 - val_mean_absolute_error: 0.1505
Epoch 751/1000
1/1 [==============================] - 0s 93ms/step - loss: 0.0088 - mean_absolute_error: 0.0741 - val_loss: 0.03
73 - val_mean_absolute_error: 0.1422
Epoch 752/1000
1/1 [==============================] - 0s 92ms/step - loss: 0.0088 - mean_absolute_error: 0.0734 - val_loss: 0.04
01 - val_mean_absolute_error: 0.1468
Epoch 753/1000
1/1 [==============================] - 0s 92ms/step - loss: 0.0085 - mean_absolute_error: 0.0724 - val_loss: 0.04
17 - val_mean_absolute_error: 0.1509
Epoch 754/1000
```

```
1/1 [==============================] - 0s 92ms/step - loss: 0.0085 - mean_absolute_error: 0.0716 - val_loss: 0.03
93 - val_mean_absolute_error: 0.1448
Epoch 755/1000
1/1 [==============================] - 0s 92ms/step - loss: 0.0083 - mean_absolute_error: 0.0713 - val_loss: 0.04
53 - val_mean_absolute_error: 0.1569
Epoch 756/1000
1/1 [==============================] - 0s 92ms/step - loss: 0.0082 - mean_absolute_error: 0.0713 - val_loss: 0.04
36 - val_mean_absolute_error: 0.1545
Epoch 757/1000
1/1 [==============================] - 0s 92ms/step - loss: 0.0079 - mean_absolute_error: 0.0696 - val_loss: 0.04
13 - val_mean_absolute_error: 0.1482
Epoch 758/1000
1/1 [==============================] - 0s 92ms/step - loss: 0.0080 - mean_absolute_error: 0.0700 - val_loss: 0.04
17 - val_mean_absolute_error: 0.1500
Epoch 759/1000
1/1 [==============================] - 0s 91ms/step - loss: 0.0076 - mean_absolute_error: 0.0684 - val_loss: 0.04
02 - val_mean_absolute_error: 0.1464
Epoch 760/1000
1/1 [==============================] - 0s 92ms/step - loss: 0.0080 - mean_absolute_error: 0.0695 - val_loss: 0.04
14 - val_mean_absolute_error: 0.1517
Epoch 761/1000
1/1 [==============================] - 0s 91ms/step - loss: 0.0080 - mean_absolute_error: 0.0704 - val_loss: 0.03
96 - val_mean_absolute_error: 0.1424
Epoch 762/1000
1/1 [==============================] - 0s 93ms/step - loss: 0.0078 - mean_absolute_error: 0.0694 - val_loss: 0.04
21 - val_mean_absolute_error: 0.1531
Epoch 763/1000
1/1 [==============================] - 0s 92ms/step - loss: 0.0077 - mean_absolute_error: 0.0687 - val_loss: 0.04
36 - val_mean_absolute_error: 0.1532
Epoch 764/1000
1/1 [==============================] - 0s 93ms/step - loss: 0.0079 - mean_absolute_error: 0.0689 - val_loss: 0.04
04 - val_mean_absolute_error: 0.1479
Epoch 765/1000
1/1 [==============================] - 0s 92ms/step - loss: 0.0076 - mean_absolute_error: 0.0676 - val_loss: 0.04
32 - val_mean_absolute_error: 0.1555
Epoch 766/1000
1/1 [==============================] - 0s 93ms/step - loss: 0.0075 - mean_absolute_error: 0.0670 - val_loss: 0.04
45 - val_mean_absolute_error: 0.1560
Epoch 767/1000
1/1 [==============================] - 0s 92ms/step - loss: 0.0075 - mean_absolute_error: 0.0673 - val_loss: 0.04
04 - val_mean_absolute_error: 0.1466
Epoch 768/1000
1/1 [==============================] - 0s 93ms/step - loss: 0.0074 - mean_absolute_error: 0.0664 - val_loss: 0.04
37 - val_mean_absolute_error: 0.1519
Epoch 769/1000
1/1 [==============================] - 0s 93ms/step - loss: 0.0074 - mean_absolute_error: 0.0660 - val_loss: 0.04
37 - val_mean_absolute_error: 0.1527
Epoch 770/1000
1/1 [==============================] - 0s 95ms/step - loss: 0.0073 - mean_absolute_error: 0.0660 - val_loss: 0.04
01 - val_mean_absolute_error: 0.1473
Epoch 771/1000
1/1 [==============================] - 0s 92ms/step - loss: 0.0073 - mean_absolute_error: 0.0658 - val_loss: 0.04
43 - val_mean_absolute_error: 0.1557
Epoch 772/1000
1/1 [==============================] - 0s 91ms/step - loss: 0.0073 - mean_absolute_error: 0.0659 - val_loss: 0.04
47 - val_mean_absolute_error: 0.1585
Epoch 773/1000
1/1 [==============================] - 0s 92ms/step - loss: 0.0071 - mean_absolute_error: 0.0653 - val_loss: 0.04
49 - val_mean_absolute_error: 0.1587
Epoch 774/1000
1/1 [==============================] - 0s 91ms/step - loss: 0.0069 - mean_absolute_error: 0.0637 - val_loss: 0.04
52 - val_mean_absolute_error: 0.1581
Epoch 775/1000
1/1 [==============================] - 0s 92ms/step - loss: 0.0070 - mean_absolute_error: 0.0647 - val_loss: 0.04
37 - val_mean_absolute_error: 0.1554
Epoch 776/1000
1/1 [==============================] - 0s 92ms/step - loss: 0.0069 - mean_absolute_error: 0.0638 - val_loss: 0.04
61 - val_mean_absolute_error: 0.1612
Epoch 777/1000
1/1 [==============================] - 0s 92ms/step - loss: 0.0068 - mean_absolute_error: 0.0637 - val_loss: 0.04
37 - val_mean_absolute_error: 0.1541
Epoch 778/1000
1/1 [==============================] - 0s 91ms/step - loss: 0.0068 - mean_absolute_error: 0.0628 - val_loss: 0.04
53 - val_mean_absolute_error: 0.1613
Epoch 779/1000
1/1 [==============================] - 0s 91ms/step - loss: 0.0070 - mean_absolute_error: 0.0651 - val_loss: 0.04
20 - val_mean_absolute_error: 0.1480
Epoch 780/1000
1/1 [==============================] - 0s 91ms/step - loss: 0.0072 - mean_absolute_error: 0.0657 - val_loss: 0.04
38 - val_mean_absolute_error: 0.1599
Epoch 781/1000
1/1 [==============================] - 0s 91ms/step - loss: 0.0069 - mean_absolute_error: 0.0647 - val_loss: 0.04
22 - val_mean_absolute_error: 0.1533
```

```
Epoch 782/1000
1/1 [==============================] - 0s 91ms/step - loss: 0.0066 - mean_absolute_error: 0.0622 - val_loss: 0.04
16 - val_mean_absolute_error: 0.1519
Epoch 783/1000
1/1 [==============================] - 0s 91ms/step - loss: 0.0066 - mean_absolute_error: 0.0619 - val_loss: 0.04
38 - val_mean_absolute_error: 0.1597
Epoch 784/1000
1/1 [==============================] - 0s 91ms/step - loss: 0.0068 - mean_absolute_error: 0.0644 - val_loss: 0.04
10 - val_mean_absolute_error: 0.1485
Epoch 785/1000
1/1 [==============================] - 0s 91ms/step - loss: 0.0071 - mean_absolute_error: 0.0656 - val_loss: 0.04
38 - val_mean_absolute_error: 0.1605
Epoch 786/1000
1/1 [==============================] - 0s 92ms/step - loss: 0.0067 - mean_absolute_error: 0.0630 - val_loss: 0.04
50 - val_mean_absolute_error: 0.1619
Epoch 787/1000
1/1 [==============================] - 0s 91ms/step - loss: 0.0067 - mean_absolute_error: 0.0637 - val_loss: 0.04
20 - val_mean_absolute_error: 0.1524
Epoch 788/1000
1/1 [==============================] - 0s 92ms/step - loss: 0.0067 - mean_absolute_error: 0.0630 - val_loss: 0.04
32 - val_mean_absolute_error: 0.1586
Epoch 789/1000
1/1 [==============================] - 0s 92ms/step - loss: 0.0066 - mean_absolute_error: 0.0628 - val_loss: 0.04
62 - val_mean_absolute_error: 0.1646
Epoch 790/1000
1/1 [==============================] - 0s 93ms/step - loss: 0.0067 - mean_absolute_error: 0.0641 - val_loss: 0.04
20 - val_mean_absolute_error: 0.1544
Epoch 791/1000
1/1 [==============================] - 0s 92ms/step - loss: 0.0066 - mean_absolute_error: 0.0624 - val_loss: 0.04
35 - val_mean_absolute_error: 0.1588
Epoch 792/1000
1/1 [==============================] - 0s 94ms/step - loss: 0.0064 - mean_absolute_error: 0.0615 - val_loss: 0.04
57 - val_mean_absolute_error: 0.1640
Epoch 793/1000
1/1 [==============================] - 0s 93ms/step - loss: 0.0064 - mean_absolute_error: 0.0617 - val_loss: 0.04
38 - val_mean_absolute_error: 0.1585
Epoch 794/1000
1/1 [==============================] - 0s 92ms/step - loss: 0.0066 - mean_absolute_error: 0.0622 - val_loss: 0.04
52 - val_mean_absolute_error: 0.1631
Epoch 795/1000
1/1 [==============================] - 0s 91ms/step - loss: 0.0063 - mean_absolute_error: 0.0617 - val_loss: 0.04
36 - val_mean_absolute_error: 0.1585
Epoch 796/1000
1/1 [==============================] - 0s 91ms/step - loss: 0.0062 - mean_absolute_error: 0.0603 - val_loss: 0.04
26 - val_mean_absolute_error: 0.1562
Epoch 797/1000
1/1 [==============================] - 0s 91ms/step - loss: 0.0063 - mean_absolute_error: 0.0607 - val_loss: 0.04
43 - val_mean_absolute_error: 0.1619
Epoch 798/1000
1/1 [==============================] - 0s 91ms/step - loss: 0.0062 - mean_absolute_error: 0.0609 - val_loss: 0.04
34 - val_mean_absolute_error: 0.1594
Epoch 799/1000
1/1 [==============================] - 0s 91ms/step - loss: 0.0062 - mean_absolute_error: 0.0602 - val_loss: 0.04
42 - val_mean_absolute_error: 0.1612
Epoch 800/1000
1/1 [==============================] - 0s 92ms/step - loss: 0.0062 - mean_absolute_error: 0.0607 - val_loss: 0.04
53 - val_mean_absolute_error: 0.1629
Epoch 801/1000
1/1 [==============================] - 0s 91ms/step - loss: 0.0062 - mean_absolute_error: 0.0605 - val_loss: 0.04
46 - val_mean_absolute_error: 0.1629
Epoch 802/1000
1/1 [==============================] - 0s 91ms/step - loss: 0.0061 - mean_absolute_error: 0.0600 - val_loss: 0.04
46 - val_mean_absolute_error: 0.1620
Epoch 803/1000
1/1 [==============================] - 0s 92ms/step - loss: 0.0060 - mean_absolute_error: 0.0593 - val_loss: 0.04
56 - val_mean_absolute_error: 0.1630
Epoch 804/1000
1/1 [==============================] - 0s 90ms/step - loss: 0.0060 - mean_absolute_error: 0.0594 - val_loss: 0.04
49 - val_mean_absolute_error: 0.1626
Epoch 805/1000
1/1 [==============================] - 0s 91ms/step - loss: 0.0060 - mean_absolute_error: 0.0592 - val_loss: 0.04
48 - val_mean_absolute_error: 0.1617
Epoch 806/1000
1/1 [==============================] - 0s 92ms/step - loss: 0.0060 - mean_absolute_error: 0.0583 - val_loss: 0.04
51 - val_mean_absolute_error: 0.1627
Epoch 807/1000
1/1 [==============================] - 0s 91ms/step - loss: 0.0059 - mean_absolute_error: 0.0586 - val_loss: 0.04
52 - val_mean_absolute_error: 0.1634
Epoch 808/1000
1/1 [==============================] - 0s 91ms/step - loss: 0.0059 - mean_absolute_error: 0.0591 - val_loss: 0.04
53 - val_mean_absolute_error: 0.1631
Epoch 809/1000
1/1 [==============================] - 0s 92ms/step - loss: 0.0059 - mean_absolute_error: 0.0584 - val_loss: 0.04
```

```
58 - val_mean_absolute_error: 0.1644
Epoch 810/1000
1/1 [==============================] - 0s 92ms/step - loss: 0.0059 - mean_absolute_error: 0.0585 - val_loss: 0.04
52 - val_mean_absolute_error: 0.1609
Epoch 811/1000
1/1 [==============================] - 0s 91ms/step - loss: 0.0059 - mean_absolute_error: 0.0581 - val_loss: 0.04
70 - val_mean_absolute_error: 0.1669
Epoch 812/1000
1/1 [==============================] - 0s 92ms/step - loss: 0.0058 - mean_absolute_error: 0.0579 - val_loss: 0.04
68 - val_mean_absolute_error: 0.1670
Epoch 813/1000
1/1 [==============================] - 0s 93ms/step - loss: 0.0058 - mean_absolute_error: 0.0579 - val_loss: 0.04
52 - val_mean_absolute_error: 0.1626
Epoch 814/1000
1/1 [==============================] - 0s 92ms/step - loss: 0.0058 - mean_absolute_error: 0.0579 - val_loss: 0.04
65 - val_mean_absolute_error: 0.1672
Epoch 815/1000
1/1 [==============================] - 0s 92ms/step - loss: 0.0057 - mean_absolute_error: 0.0579 - val_loss: 0.04
71 - val_mean_absolute_error: 0.1677
Epoch 816/1000
1/1 [==============================] - 0s 92ms/step - loss: 0.0058 - mean_absolute_error: 0.0578 - val_loss: 0.04
67 - val_mean_absolute_error: 0.1669
Epoch 817/1000
1/1 [==============================] - 0s 91ms/step - loss: 0.0057 - mean_absolute_error: 0.0579 - val_loss: 0.04
70 - val_mean_absolute_error: 0.1664
Epoch 818/1000
1/1 [==============================] - 0s 92ms/step - loss: 0.0057 - mean_absolute_error: 0.0571 - val_loss: 0.04
82 - val_mean_absolute_error: 0.1702
Epoch 819/1000
1/1 [==============================] - 0s 92ms/step - loss: 0.0057 - mean_absolute_error: 0.0577 - val_loss: 0.04
59 - val_mean_absolute_error: 0.1643
Epoch 820/1000
1/1 [==============================] - 0s 93ms/step - loss: 0.0058 - mean_absolute_error: 0.0580 - val_loss: 0.04
75 - val_mean_absolute_error: 0.1685
Epoch 821/1000
1/1 [==============================] - 0s 92ms/step - loss: 0.0057 - mean_absolute_error: 0.0572 - val_loss: 0.04
78 - val_mean_absolute_error: 0.1686
Epoch 822/1000
1/1 [==============================] - 0s 92ms/step - loss: 0.0056 - mean_absolute_error: 0.0572 - val_loss: 0.04
70 - val_mean_absolute_error: 0.1656
Epoch 823/1000
1/1 [==============================] - 0s 92ms/step - loss: 0.0056 - mean_absolute_error: 0.0572 - val_loss: 0.04
81 - val_mean_absolute_error: 0.1688
Epoch 824/1000
1/1 [==============================] - 0s 92ms/step - loss: 0.0056 - mean_absolute_error: 0.0574 - val_loss: 0.04
72 - val_mean_absolute_error: 0.1675
Epoch 825/1000
1/1 [==============================] - 0s 92ms/step - loss: 0.0056 - mean_absolute_error: 0.0571 - val_loss: 0.04
72 - val_mean_absolute_error: 0.1683
Epoch 826/1000
1/1 [==============================] - 0s 92ms/step - loss: 0.0056 - mean_absolute_error: 0.0571 - val_loss: 0.04
75 - val_mean_absolute_error: 0.1676
Epoch 827/1000
1/1 [==============================] - 0s 92ms/step - loss: 0.0056 - mean_absolute_error: 0.0566 - val_loss: 0.04
73 - val_mean_absolute_error: 0.1670
Epoch 828/1000
1/1 [==============================] - 0s 91ms/step - loss: 0.0055 - mean_absolute_error: 0.0566 - val_loss: 0.04
66 - val_mean_absolute_error: 0.1658
Epoch 829/1000
1/1 [==============================] - 0s 91ms/step - loss: 0.0055 - mean_absolute_error: 0.0562 - val_loss: 0.04
75 - val_mean_absolute_error: 0.1692
Epoch 830/1000
1/1 [==============================] - 0s 92ms/step - loss: 0.0055 - mean_absolute_error: 0.0568 - val_loss: 0.04
75 - val_mean_absolute_error: 0.1681
Epoch 831/1000
1/1 [==============================] - 0s 91ms/step - loss: 0.0055 - mean_absolute_error: 0.0559 - val_loss: 0.04
75 - val_mean_absolute_error: 0.1675
Epoch 832/1000
1/1 [==============================] - 0s 91ms/step - loss: 0.0055 - mean_absolute_error: 0.0559 - val_loss: 0.04
81 - val_mean_absolute_error: 0.1693
Epoch 833/1000
1/1 [==============================] - 0s 91ms/step - loss: 0.0055 - mean_absolute_error: 0.0564 - val_loss: 0.04
62 - val_mean_absolute_error: 0.1646
Epoch 834/1000
1/1 [==============================] - 0s 91ms/step - loss: 0.0055 - mean_absolute_error: 0.0565 - val_loss: 0.04
76 - val_mean_absolute_error: 0.1694
Epoch 835/1000
1/1 [==============================] - 0s 90ms/step - loss: 0.0056 - mean_absolute_error: 0.0573 - val_loss: 0.04
83 - val_mean_absolute_error: 0.1688
Epoch 836/1000
1/1 [==============================] - 0s 101ms/step - loss: 0.0055 - mean_absolute_error: 0.0566 - val_loss: 0.0
486 - val_mean_absolute_error: 0.1702
Epoch 837/1000
```

```
1/1 [==============================] - 0s 90ms/step - loss: 0.0055 - mean_absolute_error: 0.0568 - val_loss: 0.04
82 - val_mean_absolute_error: 0.1698
Epoch 838/1000
1/1 [==============================] - 0s 91ms/step - loss: 0.0054 - mean_absolute_error: 0.0555 - val_loss: 0.04
77 - val_mean_absolute_error: 0.1698
Epoch 839/1000
1/1 [==============================] - 0s 90ms/step - loss: 0.0054 - mean_absolute_error: 0.0557 - val_loss: 0.04
75 - val_mean_absolute_error: 0.1690
Epoch 840/1000
1/1 [==============================] - 0s 91ms/step - loss: 0.0054 - mean_absolute_error: 0.0558 - val_loss: 0.04
80 - val_mean_absolute_error: 0.1687
Epoch 841/1000
1/1 [==============================] - 0s 91ms/step - loss: 0.0054 - mean_absolute_error: 0.0557 - val_loss: 0.04
86 - val_mean_absolute_error: 0.1705
Epoch 842/1000
1/1 [==============================] - 0s 91ms/step - loss: 0.0054 - mean_absolute_error: 0.0566 - val_loss: 0.04
71 - val_mean_absolute_error: 0.1663
Epoch 843/1000
1/1 [==============================] - 0s 91ms/step - loss: 0.0055 - mean_absolute_error: 0.0563 - val_loss: 0.04
84 - val_mean_absolute_error: 0.1714
Epoch 844/1000
1/1 [==============================] - 0s 91ms/step - loss: 0.0055 - mean_absolute_error: 0.0568 - val_loss: 0.04
79 - val_mean_absolute_error: 0.1685
Epoch 845/1000
1/1 [==============================] - 0s 92ms/step - loss: 0.0054 - mean_absolute_error: 0.0555 - val_loss: 0.04
83 - val_mean_absolute_error: 0.1691
Epoch 846/1000
1/1 [==============================] - 0s 93ms/step - loss: 0.0053 - mean_absolute_error: 0.0556 - val_loss: 0.04
91 - val_mean_absolute_error: 0.1715
Epoch 847/1000
1/1 [==============================] - 0s 93ms/step - loss: 0.0053 - mean_absolute_error: 0.0555 - val_loss: 0.04
83 - val_mean_absolute_error: 0.1694
Epoch 848/1000
1/1 [==============================] - 0s 94ms/step - loss: 0.0053 - mean_absolute_error: 0.0552 - val_loss: 0.04
77 - val_mean_absolute_error: 0.1691
Epoch 849/1000
1/1 [==============================] - 0s 96ms/step - loss: 0.0054 - mean_absolute_error: 0.0560 - val_loss: 0.04
84 - val_mean_absolute_error: 0.1699
Epoch 850/1000
1/1 [==============================] - 0s 97ms/step - loss: 0.0053 - mean_absolute_error: 0.0551 - val_loss: 0.04
91 - val_mean_absolute_error: 0.1717
Epoch 851/1000
1/1 [==============================] - 0s 95ms/step - loss: 0.0053 - mean_absolute_error: 0.0554 - val_loss: 0.04
75 - val_mean_absolute_error: 0.1670
Epoch 852/1000
1/1 [==============================] - 0s 95ms/step - loss: 0.0053 - mean_absolute_error: 0.0550 - val_loss: 0.04
87 - val_mean_absolute_error: 0.1712
Epoch 853/1000
1/1 [==============================] - 0s 94ms/step - loss: 0.0053 - mean_absolute_error: 0.0558 - val_loss: 0.04
81 - val_mean_absolute_error: 0.1692
Epoch 854/1000
1/1 [==============================] - 0s 93ms/step - loss: 0.0053 - mean_absolute_error: 0.0550 - val_loss: 0.04
78 - val_mean_absolute_error: 0.1674
Epoch 855/1000
1/1 [==============================] - 0s 93ms/step - loss: 0.0052 - mean_absolute_error: 0.0546 - val_loss: 0.04
86 - val_mean_absolute_error: 0.1698
Epoch 856/1000
1/1 [==============================] - 0s 93ms/step - loss: 0.0053 - mean_absolute_error: 0.0550 - val_loss: 0.04
76 - val_mean_absolute_error: 0.1670
Epoch 857/1000
1/1 [==============================] - 0s 93ms/step - loss: 0.0052 - mean_absolute_error: 0.0545 - val_loss: 0.04
75 - val_mean_absolute_error: 0.1681
Epoch 858/1000
1/1 [==============================] - 0s 93ms/step - loss: 0.0052 - mean_absolute_error: 0.0545 - val_loss: 0.04
81 - val_mean_absolute_error: 0.1697
Epoch 859/1000
1/1 [==============================] - 0s 92ms/step - loss: 0.0052 - mean_absolute_error: 0.0548 - val_loss: 0.04
76 - val_mean_absolute_error: 0.1667
Epoch 860/1000
1/1 [==============================] - 0s 92ms/step - loss: 0.0052 - mean_absolute_error: 0.0546 - val_loss: 0.04
83 - val_mean_absolute_error: 0.1690
Epoch 861/1000
1/1 [==============================] - 0s 91ms/step - loss: 0.0052 - mean_absolute_error: 0.0548 - val_loss: 0.04
76 - val_mean_absolute_error: 0.1671
Epoch 862/1000
1/1 [==============================] - 0s 93ms/step - loss: 0.0052 - mean_absolute_error: 0.0544 - val_loss: 0.04
70 - val_mean_absolute_error: 0.1658
Epoch 863/1000
1/1 [==============================] - 0s 92ms/step - loss: 0.0052 - mean_absolute_error: 0.0548 - val_loss: 0.04
79 - val_mean_absolute_error: 0.1684
Epoch 864/1000
1/1 [==============================] - 0s 92ms/step - loss: 0.0051 - mean_absolute_error: 0.0541 - val_loss: 0.04
86 - val_mean_absolute_error: 0.1698
```

```
Epoch 865/1000
1/1 [==============================] - 0s 92ms/step - loss: 0.0052 - mean_absolute_error: 0.0541 - val_loss: 0.04
77 - val_mean_absolute_error: 0.1673
Epoch 866/1000
1/1 [==============================] - 0s 92ms/step - loss: 0.0051 - mean_absolute_error: 0.0541 - val_loss: 0.04
76 - val_mean_absolute_error: 0.1671
Epoch 867/1000
1/1 [==============================] - 0s 91ms/step - loss: 0.0051 - mean_absolute_error: 0.0541 - val_loss: 0.04
78 - val_mean_absolute_error: 0.1685
Epoch 868/1000
1/1 [==============================] - 0s 92ms/step - loss: 0.0051 - mean_absolute_error: 0.0541 - val_loss: 0.04
76 - val_mean_absolute_error: 0.1674
Epoch 869/1000
1/1 [==============================] - 0s 92ms/step - loss: 0.0051 - mean_absolute_error: 0.0541 - val_loss: 0.04
80 - val_mean_absolute_error: 0.1680
Epoch 870/1000
1/1 [==============================] - 0s 102ms/step - loss: 0.0051 - mean_absolute_error: 0.0541 - val_loss: 0.0
477 - val_mean_absolute_error: 0.1674
Epoch 871/1000
1/1 [==============================] - 0s 93ms/step - loss: 0.0051 - mean_absolute_error: 0.0542 - val_loss: 0.04
74 - val_mean_absolute_error: 0.1661
Epoch 872/1000
1/1 [==============================] - 0s 93ms/step - loss: 0.0051 - mean_absolute_error: 0.0540 - val_loss: 0.04
78 - val_mean_absolute_error: 0.1674
Epoch 873/1000
1/1 [==============================] - 0s 92ms/step - loss: 0.0051 - mean_absolute_error: 0.0537 - val_loss: 0.04
74 - val_mean_absolute_error: 0.1662
Epoch 874/1000
1/1 [==============================] - 0s 92ms/step - loss: 0.0050 - mean_absolute_error: 0.0536 - val_loss: 0.04
74 - val_mean_absolute_error: 0.1660
Epoch 875/1000
1/1 [==============================] - 0s 92ms/step - loss: 0.0050 - mean_absolute_error: 0.0537 - val_loss: 0.04
83 - val_mean_absolute_error: 0.1686
Epoch 876/1000
1/1 [==============================] - 0s 92ms/step - loss: 0.0051 - mean_absolute_error: 0.0540 - val_loss: 0.04
66 - val_mean_absolute_error: 0.1633
Epoch 877/1000
1/1 [==============================] - 0s 92ms/step - loss: 0.0051 - mean_absolute_error: 0.0539 - val_loss: 0.04
80 - val_mean_absolute_error: 0.1683
Epoch 878/1000
1/1 [==============================] - 0s 92ms/step - loss: 0.0051 - mean_absolute_error: 0.0542 - val_loss: 0.04
82 - val_mean_absolute_error: 0.1681
Epoch 879/1000
1/1 [==============================] - 0s 92ms/step - loss: 0.0051 - mean_absolute_error: 0.0537 - val_loss: 0.04
78 - val_mean_absolute_error: 0.1670
Epoch 880/1000
1/1 [==============================] - 0s 93ms/step - loss: 0.0051 - mean_absolute_error: 0.0539 - val_loss: 0.04
73 - val_mean_absolute_error: 0.1666
Epoch 881/1000
1/1 [==============================] - 0s 93ms/step - loss: 0.0050 - mean_absolute_error: 0.0535 - val_loss: 0.04
73 - val_mean_absolute_error: 0.1670
Epoch 882/1000
1/1 [==============================] - 0s 91ms/step - loss: 0.0050 - mean_absolute_error: 0.0533 - val_loss: 0.04
74 - val_mean_absolute_error: 0.1673
Epoch 883/1000
1/1 [==============================] - 0s 92ms/step - loss: 0.0050 - mean_absolute_error: 0.0536 - val_loss: 0.04
82 - val_mean_absolute_error: 0.1678
Epoch 884/1000
1/1 [==============================] - 0s 92ms/step - loss: 0.0050 - mean_absolute_error: 0.0535 - val_loss: 0.04
68 - val_mean_absolute_error: 0.1649
Epoch 885/1000
1/1 [==============================] - 0s 91ms/step - loss: 0.0050 - mean_absolute_error: 0.0536 - val_loss: 0.04
75 - val_mean_absolute_error: 0.1680
Epoch 886/1000
1/1 [==============================] - 0s 94ms/step - loss: 0.0050 - mean_absolute_error: 0.0534 - val_loss: 0.04
66 - val_mean_absolute_error: 0.1647
Epoch 887/1000
1/1 [==============================] - 0s 97ms/step - loss: 0.0050 - mean_absolute_error: 0.0532 - val_loss: 0.04
78 - val_mean_absolute_error: 0.1683
Epoch 888/1000
1/1 [==============================] - 0s 93ms/step - loss: 0.0050 - mean_absolute_error: 0.0542 - val_loss: 0.04
76 - val_mean_absolute_error: 0.1665
Epoch 889/1000
1/1 [==============================] - 0s 91ms/step - loss: 0.0051 - mean_absolute_error: 0.0541 - val_loss: 0.04
85 - val_mean_absolute_error: 0.1692
Epoch 890/1000
1/1 [==============================] - 0s 92ms/step - loss: 0.0051 - mean_absolute_error: 0.0547 - val_loss: 0.04
57 - val_mean_absolute_error: 0.1607
Epoch 891/1000
1/1 [==============================] - 0s 91ms/step - loss: 0.0051 - mean_absolute_error: 0.0546 - val_loss: 0.04
85 - val_mean_absolute_error: 0.1695
Epoch 892/1000
1/1 [==============================] - 0s 92ms/step - loss: 0.0051 - mean_absolute_error: 0.0548 - val_loss: 0.04
```

```
72 - val_mean_absolute_error: 0.1654
Epoch 893/1000
1/1 [==============================] - 0s 91ms/step - loss: 0.0050 - mean_absolute_error: 0.0536 - val_loss: 0.04
78 - val_mean_absolute_error: 0.1655
Epoch 894/1000
1/1 [==============================] - 0s 91ms/step - loss: 0.0050 - mean_absolute_error: 0.0530 - val_loss: 0.04
85 - val_mean_absolute_error: 0.1693
Epoch 895/1000
1/1 [==============================] - 0s 92ms/step - loss: 0.0050 - mean_absolute_error: 0.0537 - val_loss: 0.04
42 - val_mean_absolute_error: 0.1590
Epoch 896/1000
1/1 [==============================] - 0s 91ms/step - loss: 0.0052 - mean_absolute_error: 0.0552 - val_loss: 0.04
83 - val_mean_absolute_error: 0.1695
Epoch 897/1000
1/1 [==============================] - 0s 92ms/step - loss: 0.0052 - mean_absolute_error: 0.0557 - val_loss: 0.04
80 - val_mean_absolute_error: 0.1667
Epoch 898/1000
1/1 [==============================] - 0s 91ms/step - loss: 0.0051 - mean_absolute_error: 0.0547 - val_loss: 0.04
72 - val_mean_absolute_error: 0.1628
Epoch 899/1000
1/1 [==============================] - 0s 91ms/step - loss: 0.0050 - mean_absolute_error: 0.0540 - val_loss: 0.04
84 - val_mean_absolute_error: 0.1685
Epoch 900/1000
1/1 [==============================] - 0s 92ms/step - loss: 0.0050 - mean_absolute_error: 0.0542 - val_loss: 0.04
48 - val_mean_absolute_error: 0.1598
Epoch 901/1000
1/1 [==============================] - 0s 91ms/step - loss: 0.0052 - mean_absolute_error: 0.0551 - val_loss: 0.04
57 - val_mean_absolute_error: 0.1633
Epoch 902/1000
1/1 [==============================] - 0s 91ms/step - loss: 0.0052 - mean_absolute_error: 0.0549 - val_loss: 0.04
74 - val_mean_absolute_error: 0.1653
Epoch 903/1000
1/1 [==============================] - 0s 91ms/step - loss: 0.0051 - mean_absolute_error: 0.0546 - val_loss: 0.04
70 - val_mean_absolute_error: 0.1638
Epoch 904/1000
1/1 [==============================] - 0s 92ms/step - loss: 0.0050 - mean_absolute_error: 0.0531 - val_loss: 0.04
60 - val_mean_absolute_error: 0.1626
Epoch 905/1000
1/1 [==============================] - 0s 91ms/step - loss: 0.0049 - mean_absolute_error: 0.0527 - val_loss: 0.04
52 - val_mean_absolute_error: 0.1616
Epoch 906/1000
1/1 [==============================] - 0s 92ms/step - loss: 0.0050 - mean_absolute_error: 0.0538 - val_loss: 0.04
59 - val_mean_absolute_error: 0.1641
Epoch 907/1000
1/1 [==============================] - 0s 91ms/step - loss: 0.0050 - mean_absolute_error: 0.0540 - val_loss: 0.04
70 - val_mean_absolute_error: 0.1641
Epoch 908/1000
1/1 [==============================] - 0s 96ms/step - loss: 0.0049 - mean_absolute_error: 0.0529 - val_loss: 0.04
77 - val_mean_absolute_error: 0.1658
Epoch 909/1000
1/1 [==============================] - 0s 92ms/step - loss: 0.0049 - mean_absolute_error: 0.0532 - val_loss: 0.04
68 - val_mean_absolute_error: 0.1641
Epoch 910/1000
1/1 [==============================] - 0s 92ms/step - loss: 0.0049 - mean_absolute_error: 0.0528 - val_loss: 0.04
66 - val_mean_absolute_error: 0.1652
Epoch 911/1000
1/1 [==============================] - 0s 92ms/step - loss: 0.0049 - mean_absolute_error: 0.0528 - val_loss: 0.04
72 - val_mean_absolute_error: 0.1670
Epoch 912/1000
1/1 [==============================] - 0s 92ms/step - loss: 0.0049 - mean_absolute_error: 0.0528 - val_loss: 0.04
69 - val_mean_absolute_error: 0.1647
Epoch 913/1000
1/1 [==============================] - 0s 91ms/step - loss: 0.0049 - mean_absolute_error: 0.0525 - val_loss: 0.04
72 - val_mean_absolute_error: 0.1655
Epoch 914/1000
1/1 [==============================] - 0s 92ms/step - loss: 0.0048 - mean_absolute_error: 0.0525 - val_loss: 0.04
78 - val_mean_absolute_error: 0.1667
Epoch 915/1000
1/1 [==============================] - 0s 92ms/step - loss: 0.0048 - mean_absolute_error: 0.0523 - val_loss: 0.04
61 - val_mean_absolute_error: 0.1637
Epoch 916/1000
1/1 [==============================] - 0s 92ms/step - loss: 0.0048 - mean_absolute_error: 0.0522 - val_loss: 0.04
73 - val_mean_absolute_error: 0.1666
Epoch 917/1000
1/1 [==============================] - 0s 92ms/step - loss: 0.0048 - mean_absolute_error: 0.0522 - val_loss: 0.04
68 - val_mean_absolute_error: 0.1649
Epoch 918/1000
1/1 [==============================] - 0s 92ms/step - loss: 0.0048 - mean_absolute_error: 0.0522 - val_loss: 0.04
79 - val_mean_absolute_error: 0.1675
Epoch 919/1000
1/1 [==============================] - 0s 92ms/step - loss: 0.0048 - mean_absolute_error: 0.0525 - val_loss: 0.04
73 - val_mean_absolute_error: 0.1659
Epoch 920/1000
```

```
1/1 [==============================] - 0s 93ms/step - loss: 0.0048 - mean_absolute_error: 0.0518 - val_loss: 0.04
74 - val_mean_absolute_error: 0.1671
Epoch 921/1000
1/1 [==============================] - 0s 92ms/step - loss: 0.0048 - mean_absolute_error: 0.0520 - val_loss: 0.04
70 - val_mean_absolute_error: 0.1661
Epoch 922/1000
1/1 [==============================] - 0s 92ms/step - loss: 0.0048 - mean_absolute_error: 0.0521 - val_loss: 0.04
79 - val_mean_absolute_error: 0.1678
Epoch 923/1000
1/1 [==============================] - 0s 92ms/step - loss: 0.0047 - mean_absolute_error: 0.0518 - val_loss: 0.04
73 - val_mean_absolute_error: 0.1663
Epoch 924/1000
1/1 [==============================] - 0s 92ms/step - loss: 0.0047 - mean_absolute_error: 0.0518 - val_loss: 0.04
68 - val_mean_absolute_error: 0.1642
Epoch 925/1000
1/1 [==============================] - 0s 92ms/step - loss: 0.0047 - mean_absolute_error: 0.0518 - val_loss: 0.04
79 - val_mean_absolute_error: 0.1671
Epoch 926/1000
1/1 [==============================] - 0s 92ms/step - loss: 0.0048 - mean_absolute_error: 0.0525 - val_loss: 0.04
61 - val_mean_absolute_error: 0.1637
Epoch 927/1000
1/1 [==============================] - 0s 92ms/step - loss: 0.0048 - mean_absolute_error: 0.0528 - val_loss: 0.04
84 - val_mean_absolute_error: 0.1699
Epoch 928/1000
1/1 [==============================] - 0s 96ms/step - loss: 0.0048 - mean_absolute_error: 0.0525 - val_loss: 0.04
74 - val_mean_absolute_error: 0.1654
Epoch 929/1000
1/1 [==============================] - 0s 92ms/step - loss: 0.0048 - mean_absolute_error: 0.0519 - val_loss: 0.04
73 - val_mean_absolute_error: 0.1649
Epoch 930/1000
1/1 [==============================] - 0s 92ms/step - loss: 0.0047 - mean_absolute_error: 0.0520 - val_loss: 0.04
70 - val_mean_absolute_error: 0.1653
Epoch 931/1000
1/1 [==============================] - 0s 91ms/step - loss: 0.0047 - mean_absolute_error: 0.0515 - val_loss: 0.04
69 - val_mean_absolute_error: 0.1662
Epoch 932/1000
1/1 [==============================] - 0s 91ms/step - loss: 0.0047 - mean_absolute_error: 0.0518 - val_loss: 0.04
75 - val_mean_absolute_error: 0.1670
Epoch 933/1000
1/1 [==============================] - 0s 91ms/step - loss: 0.0047 - mean_absolute_error: 0.0517 - val_loss: 0.04
74 - val_mean_absolute_error: 0.1648
Epoch 934/1000
1/1 [==============================] - 0s 91ms/step - loss: 0.0047 - mean_absolute_error: 0.0515 - val_loss: 0.04
80 - val_mean_absolute_error: 0.1667
Epoch 935/1000
1/1 [==============================] - 0s 91ms/step - loss: 0.0047 - mean_absolute_error: 0.0518 - val_loss: 0.04
68 - val_mean_absolute_error: 0.1648
Epoch 936/1000
1/1 [==============================] - 0s 91ms/step - loss: 0.0047 - mean_absolute_error: 0.0515 - val_loss: 0.04
71 - val_mean_absolute_error: 0.1658
Epoch 937/1000
1/1 [==============================] - 0s 90ms/step - loss: 0.0047 - mean_absolute_error: 0.0515 - val_loss: 0.04
72 - val_mean_absolute_error: 0.1654
Epoch 938/1000
1/1 [==============================] - 0s 91ms/step - loss: 0.0047 - mean_absolute_error: 0.0514 - val_loss: 0.04
68 - val_mean_absolute_error: 0.1640
Epoch 939/1000
1/1 [==============================] - 0s 97ms/step - loss: 0.0047 - mean_absolute_error: 0.0513 - val_loss: 0.04
74 - val_mean_absolute_error: 0.1652
Epoch 940/1000
1/1 [==============================] - 0s 93ms/step - loss: 0.0047 - mean_absolute_error: 0.0515 - val_loss: 0.04
61 - val_mean_absolute_error: 0.1627
Epoch 941/1000
1/1 [==============================] - 0s 91ms/step - loss: 0.0047 - mean_absolute_error: 0.0513 - val_loss: 0.04
61 - val_mean_absolute_error: 0.1629
Epoch 942/1000
1/1 [==============================] - 0s 92ms/step - loss: 0.0047 - mean_absolute_error: 0.0512 - val_loss: 0.04
59 - val_mean_absolute_error: 0.1624
Epoch 943/1000
1/1 [==============================] - 0s 91ms/step - loss: 0.0046 - mean_absolute_error: 0.0513 - val_loss: 0.04
52 - val_mean_absolute_error: 0.1612
Epoch 944/1000
1/1 [==============================] - 0s 92ms/step - loss: 0.0046 - mean_absolute_error: 0.0511 - val_loss: 0.04
58 - val_mean_absolute_error: 0.1627
Epoch 945/1000
1/1 [==============================] - 0s 91ms/step - loss: 0.0046 - mean_absolute_error: 0.0510 - val_loss: 0.04
55 - val_mean_absolute_error: 0.1617
Epoch 946/1000
1/1 [==============================] - 0s 92ms/step - loss: 0.0046 - mean_absolute_error: 0.0511 - val_loss: 0.04
56 - val_mean_absolute_error: 0.1614
Epoch 947/1000
1/1 [==============================] - 0s 92ms/step - loss: 0.0046 - mean_absolute_error: 0.0511 - val_loss: 0.04
61 - val_mean_absolute_error: 0.1625
```

```
Epoch 948/1000
1/1 [==============================] - 0s 91ms/step - loss: 0.0046 - mean_absolute_error: 0.0511 - val_loss: 0.04
55 - val_mean_absolute_error: 0.1611
Epoch 949/1000
1/1 [==============================] - 0s 91ms/step - loss: 0.0046 - mean_absolute_error: 0.0513 - val_loss: 0.04
61 - val_mean_absolute_error: 0.1630
Epoch 950/1000
1/1 [==============================] - 0s 92ms/step - loss: 0.0046 - mean_absolute_error: 0.0512 - val_loss: 0.04
62 - val_mean_absolute_error: 0.1629
Epoch 951/1000
1/1 [==============================] - 0s 91ms/step - loss: 0.0047 - mean_absolute_error: 0.0515 - val_loss: 0.04
64 - val_mean_absolute_error: 0.1633
Epoch 952/1000
1/1 [==============================] - 0s 93ms/step - loss: 0.0047 - mean_absolute_error: 0.0512 - val_loss: 0.04
64 - val_mean_absolute_error: 0.1632
Epoch 953/1000
1/1 [==============================] - 0s 93ms/step - loss: 0.0046 - mean_absolute_error: 0.0513 - val_loss: 0.04
52 - val_mean_absolute_error: 0.1614
Epoch 954/1000
1/1 [==============================] - 0s 92ms/step - loss: 0.0046 - mean_absolute_error: 0.0511 - val_loss: 0.04
65 - val_mean_absolute_error: 0.1639
Epoch 955/1000
1/1 [==============================] - 0s 94ms/step - loss: 0.0046 - mean_absolute_error: 0.0510 - val_loss: 0.04
64 - val_mean_absolute_error: 0.1628
Epoch 956/1000
1/1 [==============================] - 0s 93ms/step - loss: 0.0046 - mean_absolute_error: 0.0509 - val_loss: 0.04
60 - val_mean_absolute_error: 0.1617
Epoch 957/1000
1/1 [==============================] - 0s 93ms/step - loss: 0.0046 - mean_absolute_error: 0.0512 - val_loss: 0.04
69 - val_mean_absolute_error: 0.1639
Epoch 958/1000
1/1 [==============================] - 0s 92ms/step - loss: 0.0046 - mean_absolute_error: 0.0512 - val_loss: 0.04
62 - val_mean_absolute_error: 0.1626
Epoch 959/1000
1/1 [==============================] - 0s 92ms/step - loss: 0.0046 - mean_absolute_error: 0.0511 - val_loss: 0.04
64 - val_mean_absolute_error: 0.1626
Epoch 960/1000
1/1 [==============================] - 0s 92ms/step - loss: 0.0046 - mean_absolute_error: 0.0511 - val_loss: 0.04
65 - val_mean_absolute_error: 0.1631
Epoch 961/1000
1/1 [==============================] - 0s 92ms/step - loss: 0.0046 - mean_absolute_error: 0.0509 - val_loss: 0.04
65 - val_mean_absolute_error: 0.1630
Epoch 962/1000
1/1 [==============================] - 0s 93ms/step - loss: 0.0046 - mean_absolute_error: 0.0512 - val_loss: 0.04
64 - val_mean_absolute_error: 0.1626
Epoch 963/1000
1/1 [==============================] - 0s 92ms/step - loss: 0.0046 - mean_absolute_error: 0.0510 - val_loss: 0.04
56 - val_mean_absolute_error: 0.1608
Epoch 964/1000
1/1 [==============================] - 0s 91ms/step - loss: 0.0046 - mean_absolute_error: 0.0509 - val_loss: 0.04
56 - val_mean_absolute_error: 0.1610
Epoch 965/1000
1/1 [==============================] - 0s 91ms/step - loss: 0.0046 - mean_absolute_error: 0.0506 - val_loss: 0.04
58 - val_mean_absolute_error: 0.1616
Epoch 966/1000
1/1 [==============================] - 0s 91ms/step - loss: 0.0046 - mean_absolute_error: 0.0508 - val_loss: 0.04
56 - val_mean_absolute_error: 0.1610
Epoch 967/1000
1/1 [==============================] - 0s 91ms/step - loss: 0.0046 - mean_absolute_error: 0.0508 - val_loss: 0.04
50 - val_mean_absolute_error: 0.1600
Epoch 968/1000
1/1 [==============================] - 0s 91ms/step - loss: 0.0046 - mean_absolute_error: 0.0512 - val_loss: 0.04
62 - val_mean_absolute_error: 0.1630
Epoch 969/1000
1/1 [==============================] - 0s 91ms/step - loss: 0.0046 - mean_absolute_error: 0.0514 - val_loss: 0.04
50 - val_mean_absolute_error: 0.1598
Epoch 970/1000
1/1 [==============================] - 0s 91ms/step - loss: 0.0046 - mean_absolute_error: 0.0513 - val_loss: 0.04
64 - val_mean_absolute_error: 0.1621
Epoch 971/1000
1/1 [==============================] - 0s 91ms/step - loss: 0.0046 - mean_absolute_error: 0.0512 - val_loss: 0.04
63 - val_mean_absolute_error: 0.1623
Epoch 972/1000
1/1 [==============================] - 0s 91ms/step - loss: 0.0046 - mean_absolute_error: 0.0506 - val_loss: 0.04
56 - val_mean_absolute_error: 0.1603
Epoch 973/1000
1/1 [==============================] - 0s 91ms/step - loss: 0.0046 - mean_absolute_error: 0.0511 - val_loss: 0.04
67 - val_mean_absolute_error: 0.1632
Epoch 974/1000
1/1 [==============================] - 0s 91ms/step - loss: 0.0046 - mean_absolute_error: 0.0506 - val_loss: 0.04
61 - val_mean_absolute_error: 0.1620
Epoch 975/1000
1/1 [==============================] - 0s 92ms/step - loss: 0.0045 - mean_absolute_error: 0.0505 - val_loss: 0.04
```

```
59 - val_mean_absolute_error: 0.1612
Epoch 976/1000
1/1 [==============================] - 0s 91ms/step - loss: 0.0046 - mean_absolute_error: 0.0507 - val_loss: 0.04
66 - val_mean_absolute_error: 0.1632
Epoch 977/1000
1/1 [==============================] - 0s 91ms/step - loss: 0.0046 - mean_absolute_error: 0.0508 - val_loss: 0.04
58 - val_mean_absolute_error: 0.1604
Epoch 978/1000
1/1 [==============================] - 0s 91ms/step - loss: 0.0046 - mean_absolute_error: 0.0513 - val_loss: 0.04
61 - val_mean_absolute_error: 0.1613
Epoch 979/1000
1/1 [==============================] - 0s 93ms/step - loss: 0.0046 - mean_absolute_error: 0.0510 - val_loss: 0.04
66 - val_mean_absolute_error: 0.1626
Epoch 980/1000
1/1 [==============================] - 0s 92ms/step - loss: 0.0046 - mean_absolute_error: 0.0512 - val_loss: 0.04
60 - val_mean_absolute_error: 0.1612
Epoch 981/1000
1/1 [==============================] - 0s 91ms/step - loss: 0.0045 - mean_absolute_error: 0.0505 - val_loss: 0.04
64 - val_mean_absolute_error: 0.1618
Epoch 982/1000
1/1 [==============================] - 0s 91ms/step - loss: 0.0045 - mean_absolute_error: 0.0509 - val_loss: 0.04
70 - val_mean_absolute_error: 0.1629
Epoch 983/1000
1/1 [==============================] - 0s 91ms/step - loss: 0.0045 - mean_absolute_error: 0.0508 - val_loss: 0.04
69 - val_mean_absolute_error: 0.1627
Epoch 984/1000
1/1 [==============================] - 0s 91ms/step - loss: 0.0046 - mean_absolute_error: 0.0506 - val_loss: 0.04
68 - val_mean_absolute_error: 0.1620
Epoch 985/1000
1/1 [==============================] - 0s 91ms/step - loss: 0.0046 - mean_absolute_error: 0.0508 - val_loss: 0.04
70 - val_mean_absolute_error: 0.1628
Epoch 986/1000
1/1 [==============================] - 0s 92ms/step - loss: 0.0045 - mean_absolute_error: 0.0505 - val_loss: 0.04
68 - val_mean_absolute_error: 0.1623
Epoch 987/1000
1/1 [==============================] - 0s 91ms/step - loss: 0.0045 - mean_absolute_error: 0.0503 - val_loss: 0.04
72 - val_mean_absolute_error: 0.1637
Epoch 988/1000
1/1 [==============================] - 0s 91ms/step - loss: 0.0045 - mean_absolute_error: 0.0502 - val_loss: 0.04
67 - val_mean_absolute_error: 0.1628
Epoch 989/1000
1/1 [==============================] - 0s 91ms/step - loss: 0.0045 - mean_absolute_error: 0.0504 - val_loss: 0.04
67 - val_mean_absolute_error: 0.1626
Epoch 990/1000
1/1 [==============================] - 0s 91ms/step - loss: 0.0045 - mean_absolute_error: 0.0503 - val_loss: 0.04
73 - val_mean_absolute_error: 0.1641
Epoch 991/1000
1/1 [==============================] - 0s 90ms/step - loss: 0.0045 - mean_absolute_error: 0.0503 - val_loss: 0.04
68 - val_mean_absolute_error: 0.1628
Epoch 992/1000
1/1 [==============================] - 0s 91ms/step - loss: 0.0045 - mean_absolute_error: 0.0503 - val_loss: 0.04
78 - val_mean_absolute_error: 0.1651
Epoch 993/1000
1/1 [==============================] - 0s 91ms/step - loss: 0.0045 - mean_absolute_error: 0.0502 - val_loss: 0.04
70 - val_mean_absolute_error: 0.1636
Epoch 994/1000
1/1 [==============================] - 0s 92ms/step - loss: 0.0045 - mean_absolute_error: 0.0506 - val_loss: 0.04
67 - val_mean_absolute_error: 0.1630
Epoch 995/1000
1/1 [==============================] - 0s 91ms/step - loss: 0.0045 - mean_absolute_error: 0.0504 - val_loss: 0.04
64 - val_mean_absolute_error: 0.1619
Epoch 996/1000
1/1 [==============================] - 0s 92ms/step - loss: 0.0045 - mean_absolute_error: 0.0504 - val_loss: 0.04
63 - val_mean_absolute_error: 0.1616
Epoch 997/1000
1/1 [==============================] - 0s 92ms/step - loss: 0.0045 - mean_absolute_error: 0.0501 - val_loss: 0.04
68 - val_mean_absolute_error: 0.1626
Epoch 998/1000
1/1 [==============================] - 0s 91ms/step - loss: 0.0045 - mean_absolute_error: 0.0500 - val_loss: 0.04
61 - val_mean_absolute_error: 0.1611
Epoch 999/1000
1/1 [==============================] - 0s 91ms/step - loss: 0.0045 - mean_absolute_error: 0.0500 - val_loss: 0.04
64 - val_mean_absolute_error: 0.1623
Epoch 1000/1000
1/1 [==============================] - 0s 92ms/step - loss: 0.0045 - mean_absolute_error: 0.0500 - val_loss: 0.04
65 - val_mean_absolute_error: 0.1625
2/2 [==============================] - 0s 152ms/step - loss: 0.0071 - mean_absolute_error: 0.0580
Test score: 0.007086591795086861
Test accuracy: 0.05799603462219238
```

In [12]:

```
plt.plot(history.history['mean_absolute_error'])
```

```
plt.plot(history.history['val_mean_absolute_error'])
plt.title('y_absolute_error')
plt.ylabel('absolute_error')
plt.xlabel('epochs')
plt.legend(['train', 'val'], loc='upper left')
plt.show()
```

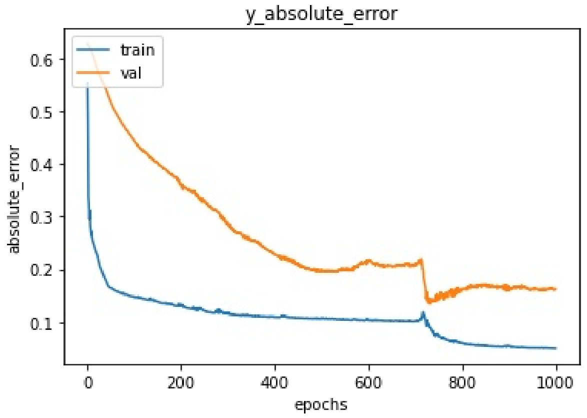

In [13]:

```
plt.plot(history.history['loss'])
plt.plot(history.history['val_loss'])
plt.title('model loss')
plt.ylabel('loss')
plt.xlabel('epochs')
plt.legend(['train', 'test'], loc='upper right')
plt.show()
```

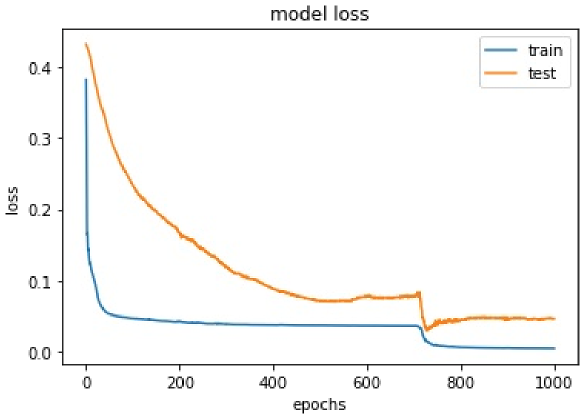

In [14]:

```
labels_predicted = model.predict(T_x)
curve_number = np.linspace(0, 80, 80)

label_pred = labels_predicted[8,:]
t_pred = label_pred[:]
print(t_pred)
print(Labels_a[8])

figure(figsize=(12,4), dpi=80)
plt.subplot(1,4,1)
plt.plot(labels_predicted[:,0], Labels_a[:,0],'.', alpha =0.3)
plt.plot(Labels_a[:,0],Labels_a[:,0])
plt.title('growth rate (G1)')
#plt.show()

plt.subplot(1,4,2)
plt.plot(labels_predicted[:,1], Labels_a[:,1],'.', alpha =0.3)
plt.plot(Labels_a[:,1],Labels_a[:,1])
plt.title('G2')
#plt.show()

plt.subplot(1,4,3)
plt.plot(labels_predicted[:,2], Labels_a[:,2],'.', alpha =0.3)
plt.plot(Labels_a[:,2],Labels_a[:,2])
plt.title('G3')
#plt.show()

plt.subplot(1,4,4)
plt.plot(labels_predicted[:,3], Labels_a[:,3],'.', alpha =0.3)
plt.plot(Labels_a[:,3],Labels_a[:,3])
plt.title('G4')
```

```
plt.show()

figure(figsize=(12,4), dpi=80)
plt.subplot(1,4,1)
plt.plot(labels_predicted[:,4], Labels_a[:,4],'.', alpha =0.3)
plt.plot(Labels_a[:,4],Labels_a[:,4])
plt.title('max starting value')
#plt.show()

plt.subplot(1,4,2)
plt.plot(labels_predicted[:,5], Labels_a[:,5],'.', alpha =0.3)
plt.plot(Labels_a[:,5],Labels_a[:,5])
plt.title('decay')
#plt.show()

plt.subplot(1,4,3)
plt.plot(labels_predicted[:,6], Labels_a[:,6],'.', alpha =0.3)
plt.plot(Labels_a[:,6],Labels_a[:,6])
plt.title('shift')
#plt.show()

plt.subplot(1,4,4)
plt.plot(labels_predicted[:,7], Labels_a[:,7],'.', alpha =0.3)
plt.plot(Labels_a[:,7],Labels_a[:,7])
plt.title('e')
plt.show()

plt.plot(labels_predicted[:,8], Labels_a[:,8],'.', alpha =0.3)
plt.plot(Labels_a[:,8],Labels_a[:,8])
plt.title('SLD')
plt.show()
```

```
[0.52193445 0.7702471  0.59170437 0.7413906  0.6476549  0.41771048
 0.44108227 0.73798597 0.33347997 0.58343375]
tf.Tensor(
[0.49896736 0.78243473 0.55462088 0.80039684 0.63180394 0.43928263
 0.37353709 0.70038843 0.36645639 0.52798539], shape=(10,), dtype=float64)
```

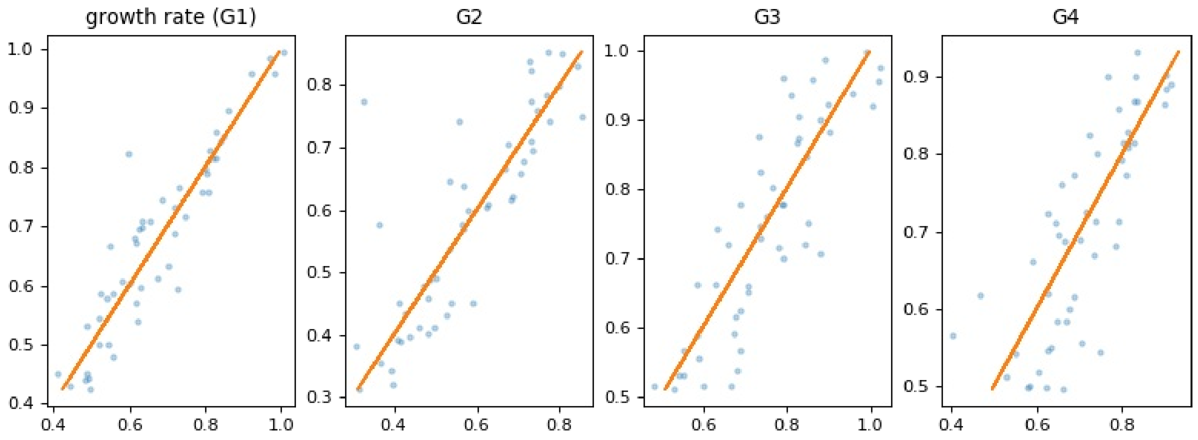

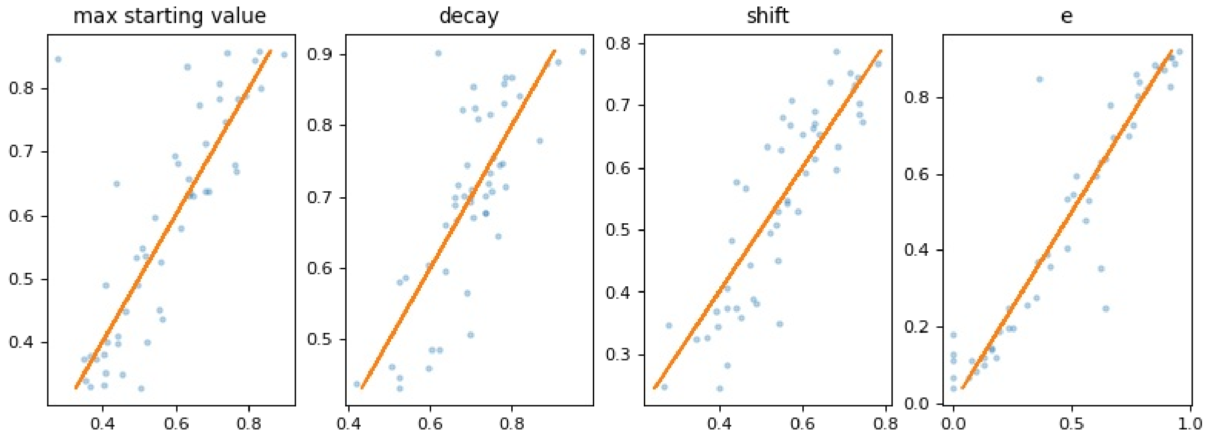

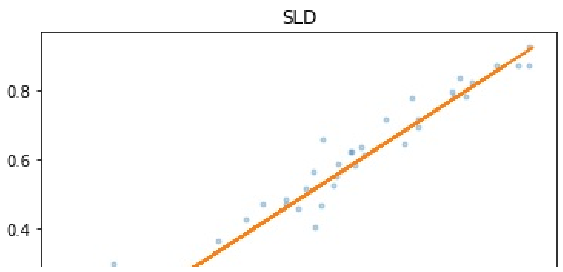

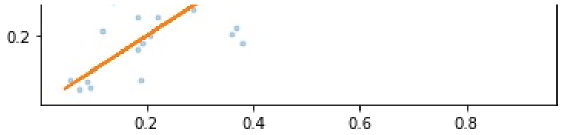

In [ ]:
