# Supplementary material for: Faster and lower-dose X-ray reflectivity measurements enabled by physics-informed modeling and artificial intelligence co-refinement
Source: J Appl Crystallogr. 2022 Oct 1;55(Pt 5):1305–13. doi: 10.1107/S2053273322008051 (PMC9533750; doi:10.1107/S2053273322008051)
Supplement: Supplementary file 2 [file j-55-01305-sup2.pdf]

JOURNAL OF
APPLIED
CRYSTALLOGRAPHY

Volume 55 (2022)

Supporting information for article:

Faster and lower-dose X-ray reflectivity measurements enabled by physics-informed modeling and artificial intelligence co-refinement

David Mareček, Julian Oberreiter, Andrew Nelson and Stefan Kowarik

In [1]:

```python
import re
import time
import os
import glob
from functools import partial, partialmethod


import math
from numpy import exp, array, insert
from math import log, sqrt, e

import numpy as np
from scipy import integrate
from scipy.optimize import differential_evolution

%matplotlib inline
import matplotlib.pyplot as plt
import matplotlib as mpl
from matplotlib.widgets import CheckButtons, Cursor

from refnx.analysis import Parameter, Parameters
from refnx.reflect import SLD, Slab, Structure, ReflectModel


# an alphanumeic sorter
def sorted_nicely(l):
    """Sort the given iterable in the way that humans expect."""
    convert = lambda text: int(text) if text.isdigit() else text
    alphanum_key = lambda key: [convert(c) for c in re.split("([0-9]+)", key)]
    return sorted(l, key=alphanum_key)
```

Simplified Trofimov-model solver

In [2]:

```python
################################################################################
###############      Trofimov-model for layer coverages         ###############
################################################################################
def Xi2(Theta, ThetaC):
    rval = np.zeros_like(Theta)

    cond0 = Theta > ThetaC
    cond1 = np.logical_and(0 < Theta, Theta < 1)
    cond2 = np.logical_and(0 < ThetaC, ThetaC < 1)
    cond3 = cond0 & cond1 & cond2
    cond4 = Theta >= 1
    cond5 = ThetaC >= 1

    rval[cond0 & cond4] = 1.0
    rval[cond0 & cond5] = 1.0

    if cond3.any():
        expo1 = np.sqrt(-np.log(1 - Theta[cond3]))
        expo2 = np.sqrt(-np.log(1 - ThetaC[cond3]))
        rval[cond3] = 1 - np.exp(-(expo1 - expo2) * (expo1 - expo2))

    return rval

def trofimov(X, t, *args):
    """
    X: list of differential equations to solve
    t: independent variable
    args: Xc, R123
        Xc: list of theta criticals
        R123 = R1,R2,R3,R: growth rates for different layers
    """
    Xc, R123 = args
    R1, R2, R3, R = R123
    N = len(Xc)
    rval = np.zeros_like(Xc)

    # Rate equations for different rates for the first four ML
    xit = Xi2(X, Xc)

    if X[0] < 0.99999999:
        rval[0] = R1 * (1 - X[0]) + R2 * (X[0] - xit[0])

    rval[1] = R2 * (xit[0] - X[1]) + R * (X[1] - xit[1])
    rval[2] = R3 * (xit[1] - X[2]) + R * (X[2] - xit[2])
    rval[3:] = R * (xit[2:-1] - xit[3:])
    return rval


################################################################################
###############     Functions for calculations      ###############
################################################################################
```

```python
def calc_all(N, t, R123, ThetaC):
    """Calculate coverages, thickness, roughness, reflectivity"""

    X0 = np.zeros(N)
    X = integrate.odeint(trofimov, X0, t, args=(ThetaC, R123), mxstep=2000) #original
    np.clip(X, None, 1.0, out=X)
    coverages = np.transpose(X)  # Coverages in a.u.
    thickness = sum(coverages)
    roughness = np.sqrt(abs(sum(
            [(coverages[i] - coverages[i + 1]) * (i + 1 - thickness) ** 2 for i in range(N - 1)]
        )
    )
    )
    roughness_f = roughness *16.6  # 16.6 Å (DIP monolayer thickness), the roughness for the experimental dataset
    thickness_f = thickness * 16.6 # 16.6 Å (DIP monolayer thickness), the thickness of the film
    return X, coverages, thickness_f, roughness_f
```

Parameters for the growth model

In [4]:

```python
N = 35  # number of ML
times = np.linspace(0, 16, num=80) #time of the growth
x = np.linspace(0, N, N)

# parameters G - growth rates
G1 = Parameter(0.9, name="G1")
G2 = Parameter(0.7, name="G2")
G3 = Parameter(0.85, name="G3")
G4 = Parameter(1.09, name="G4")

# parameters to generate Theta criticals
a = Parameter(0.28, name="a") # parameter for the tanh() function
b = Parameter(0.6, name="b")  # parameter for the tanh() function
c = Parameter(0.2, name="c")  # parameter when to swich from tanh() to exp()
d = Parameter(-5.75, name="d") # exp() decay
g = Parameter(0.028, name="g") # exp() convergation
```

Theta criticals generation based on tanh() function and an exponential decay

In [5]:

```python
def growth_model_thickness(times, N, G1, G2, G3, G4, a, b, c, d, g):
    G1, G2, G3, G4, a, b, c, d, g = map(float, [G1, G2, G3, G4, a, b, c, d, g])
    x = np.linspace(0, N, N)
    f = 0
    z = a*(((0.5*np.tanh(-0.5*(x+d))+0.5))/((0.5*np.tanh(-0.5*d)+0.5)))
    #print(z)
    for j in range (N):
        if z[j] > c:
            z[j] = z[j]
            f = j
        else:
            z[j] = (c - g)* e ** (-b * x[j-f]) + g
    #print(z)
    gr = [G1, G2, G3, G4]

    thetacrit = z
    out = calc_all(N, times, gr, thetacrit)
    return out
```

In [6]:

```python
pars = growth_model_thickness(times, N, G1, G2, G3, G4, a, b, c, d, g)
```

In [7]:

```python
plt.rcParams["font.size"] = "16"
for i in range(N):
    plt.plot(pars[0][:, i], linewidth=2)
plt.xlabel("Time [Å]", fontsize=20)
plt.ylabel("Coverage", fontsize=20)
plt.savefig("Coverage.svg", format="svg", dpi=1200)
plt.show()


# plt.figure(figsize=(8, 6), dpi=1200)
# Where on earth does this value of 16.6 come from?

plt.plot(times, pars[2], linewidth=2) #16.6 thickness of the one ML. pars[3] * 16.6 = real thickness
plt.xlabel("Time", fontsize=20)
plt.ylabel("Thickness [Å]", fontsize=20)
plt.savefig("Roughness.svg", format="svg", dpi=1200)
plt.show()

plt.plot(times, pars[3]) #16.6 thickness of the one ML. pars[3] * 16.6 = real roughness
plt.xlabel("Time", fontsize=20)
plt.ylabel("Roughness [Å]", fontsize=20)
plt.show()
```

```
print(pars[0].shape, pars[1].shape)
```

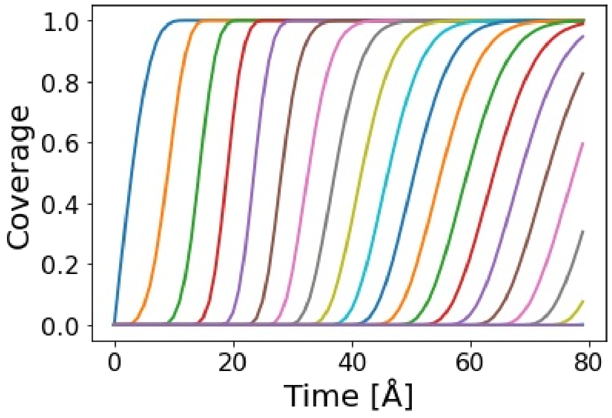

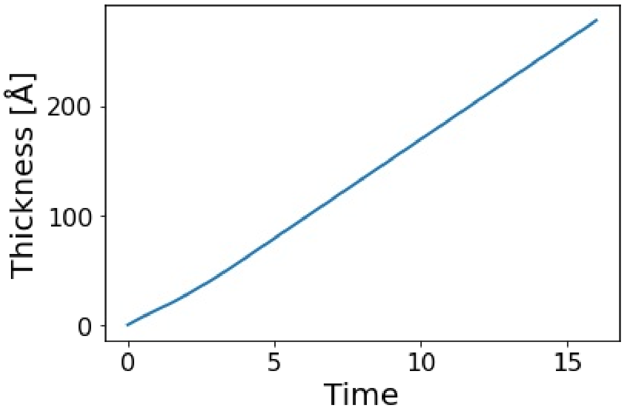

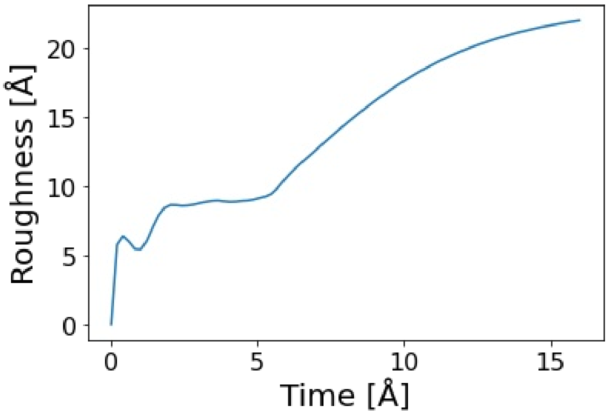


```
(80, 35) (35, 80)
```

In [8]:

```
from refnx.dataset import Data1D
from refnx.analysis import Transform, Objective, GlobalObjective, Parameter, CurveFitter

files = sorted_nicely(glob.glob("data/DIP403_*.*"))
datasets = [Data1D(file) for file in files]
print(datasets[1])
```

```
<DIP403_1>, 109 points
```

In [10]:

```
thicks = Parameters()
sigmas = Parameters()
for t in times:
    p = Parameter(1, name=f"thick - {t}s")
    sigma = Parameter(1, name=f"sigma - {t}s")
    p.t = t
    sigma.t = t
    thicks.append(p)
    sigmas.append(sigma)
```

In [11]:

```
si = SLD(20.0, "Si")
```

```
air = SLD(0, "Air")
sio2 = SLD(19, "SiO2")
film_s = SLD(10, "film")
```

In [ ]:

```
strucs = []
models = []
air_l = air()
sio2_l = sio2(10, 2.5)
si_l = si(0, 1)

for t, thick, sigma in zip(times, thicks, sigmas):
    film_l = Slab(thick, film_s, sigma)
    s = air_l | film_l | sio2_l | si
    strucs.append(s)
    mod = ReflectModel(s)
    mod.threads = 1
    mod.dq.value = 0.0
    models.append(mod)
```

In [13]:

```
q = np.linspace(0.01, 0.14, 109)
for s in strucs:
    plt.plot(q, s.reflectivity(q))

plt.yscale("log")
```

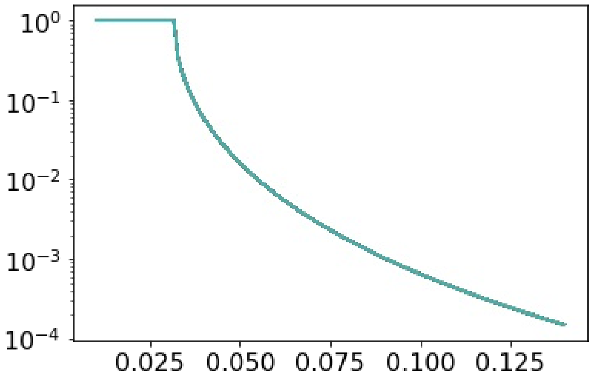

In [14]:

```
G1.setp(vary=True, bounds=(0.5, 1.1))
G2.setp(vary=True, bounds=(0.5, 1.1))
G3.setp(vary=True, bounds=(0.7, 1.2))
G4.setp(vary=True, bounds=(0.9, 1.7))
a.setp(vary=True, bounds=(0.2, 0.6)) # max 0.7, after that the integration is taking too long
b.setp(vary=True, bounds=(0.05, 0.7))
c.setp(vary=True, bounds=(0.11, 0.4))
d.setp(vary=True, bounds=(-7, 0))
g.setp(vary=True, bounds=(0.002, 0.12))

film_l.sld.real.setp(vary=True, bounds=(5, 20))
```

In [15]:

```
t = Transform("logY")

objectives = [
    Objective(model, data, use_weights=False, transform=t)
    for model, data in zip(models, datasets)
]
objectives[0].auxiliary_params = Parameters(
    data=(G1, G2, G3, G4, a, b, c, d, g)
)
global_objective = GlobalObjective(objectives)
```

In [16]:

```
def wrapper(
    global_objective, thicks, sigmas, times, N, G1, G2, G3, G4, a, b, c, d, g
):
    def cost_to_minimise(x):

        global_objective.setp(x)

        _X, _cov, ts, rs = growth_model_thickness(times, N, G1, G2, G3, G4, a, b, c, d, g)

        for thick, sigma, t, r in zip(thicks, sigmas, ts, rs):

            thick.value = t
            assert np.isfinite(t)
            sigma.value = r
            assert np.isfinite(r)
        try:
```

```python
            plp = global_objective.nll()
            return plp
        except RuntimeError as exc:
            print(global_objective.varying_parameters())
            print(ts)
            raise exc

    return cost_to_minimise

bounds = [(p.bounds.lb, p.bounds.ub) for p in global_objective.varying_parameters()]
cost = wrapper(
    global_objective, thicks, sigmas, times, N, G1, G2, G3, G4, a, b, c, d, g
)

def callback(x, *args, **kwds):
    pass
```

In [17]:

```python
cost(global_objective.varying_parameters())
```

Out[17]: 135.58709262755028

In [18]:

```python
%timeit growth_model_thickness(times, N, G1, G2, G3, G4, a, b, c, d, g)
```

301 ms ± 45.2 ms per loop (mean ± std. dev. of 7 runs, 1 loop each)

In [19]:

```python
%timeit cost(global_objective.varying_parameters())
```

367 ms ± 7.16 ms per loop (mean ± std. dev. of 7 runs, 1 loop each)

A large proportion of time seems to be spent in solving the differential equation.

In [20]:

```python
res = differential_evolution(cost, bounds, polish=True, seed=2, callback=callback)
```

In [21]:

```python
objectives[11].plot()
objectives[33].plot()
objectives[77].plot()
plt.legend()
plt.xlabel("Q")
plt.ylabel("logR");
# plt.yscale('log')
```

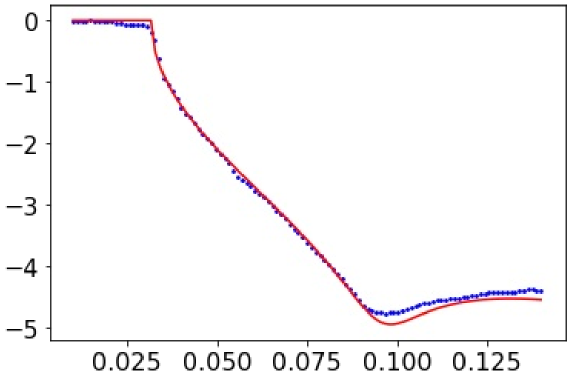

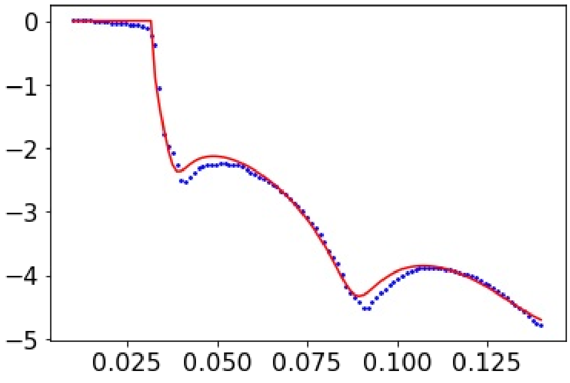

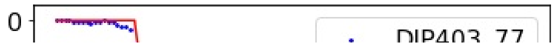

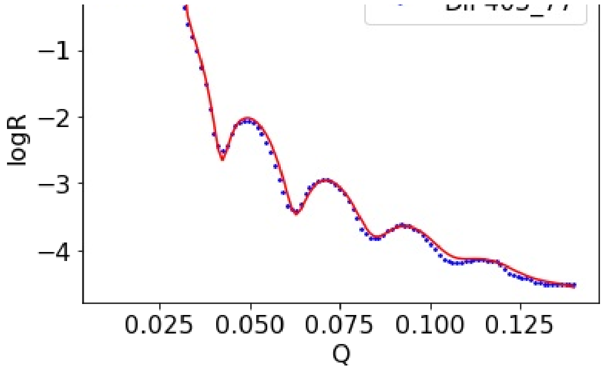

In [22]:
```
objectives[-1].model.structure.slabs()
```

Out[22]:
```
array([[  0.        ,   0.        ,   0.        ,   0.        ,
          0.        ],
       [276.07536797,  11.04124549,   0.        ,  21.77371835,
          0.        ],
       [ 10.        ,  19.        ,   0.        ,   2.5       ,
          0.        ],
       [  0.        ,  20.        ,   0.        ,   0.        ,
          0.        ]])
```

In [24]:
```
print(repr(np.array(thicks)))
print(repr(np.array(sigmas)))
```

```
array([  0.        ,   3.53801459,   6.78722055,   9.79325396,
        12.62280513,  15.35191372,  18.05017715,  20.75474562,
        23.4857939 ,  26.25526201,  29.07155125,  31.94363833,
        34.88121913,  37.89305036,  40.98628202,  44.16540043,
        47.43113822,  50.77989378,  54.20392934,  57.6923022 ,
        61.23229157,  64.81098525,  68.41667641,  72.03979746,
        75.67327806,  79.3123917 ,  82.95428403,  86.59740446,
        90.24100238,  93.8847595 ,  97.52856059, 101.17237129,
       104.81618354, 108.45999597, 112.1038084 , 115.74762083,
       119.39143327, 123.0352457 , 126.67905814, 130.32287057,
       133.96668301, 137.61049544, 141.25430788, 144.89812031,
       148.54193275, 152.18574518, 155.82955762, 159.47337005,
       163.11718249, 166.76099492, 170.40480736, 174.04861979,
       177.69243223, 181.33624466, 184.9800571 , 188.62386953,
       192.26768197, 195.9114944 , 199.55530684, 203.19911927,
       206.84293171, 210.48674414, 214.13055658, 217.77436901,
       221.41818145, 225.06199388, 228.70580632, 232.34961875,
       235.99343119, 239.63724362, 243.28105606, 246.92486849,
       250.56868093, 254.21249336, 257.8563058 , 261.50011823,
       265.14393067, 268.7877431 , 272.43155554, 276.07536797])
array([ 0.        ,  6.03026423,  6.39413024,  6.01192939,  5.83982308,
        6.21894005,  7.01122757,  7.97603698,  8.92488621,  9.74542407,
       10.3825029 , 10.83623006, 11.19174348, 11.52676964, 11.87151301,
       12.20226181, 12.50005159, 12.77869804, 13.03567041, 13.24762912,
       13.38810629, 13.48356854, 13.56529284, 13.64246753, 13.70023674,
       13.75084038, 13.82050225, 13.9133515 , 14.008254  , 14.09762658,
       14.20070674, 14.32258356, 14.44727348, 14.56165225, 14.68252077,
       14.81785379, 14.95731358, 15.0858835 , 15.21674082, 15.3592096 ,
       15.50702458, 15.64525763, 15.78307988, 15.93030038, 16.08354649,
       16.22920701, 16.37266337, 16.52368029, 16.68093757, 16.83265397,
       16.98103927, 17.13542972, 17.29594795, 17.45273774, 17.60557284,
       17.76309681, 17.9264294 , 18.08752599, 18.24440722, 18.40486837,
       18.57070117, 18.73546591, 18.89600818, 19.05920055, 19.22727063,
       19.39515032, 19.558968  , 19.72465769, 19.89471361, 20.06521277,
       20.23191416, 20.39983565, 20.57161843, 20.74428185, 20.9134701 ,
       21.08332945, 21.25656511, 21.43096349, 21.60223853, 21.77371835])
```

In [26]:
```
out = np.array([objective.generative() for objective in objectives])
#plt.figure(figsize=(7, 5), dpi=1200)
plt.imshow(np.log(out))
plt.colorbar()
```

Out[26]:
```
<matplotlib.colorbar.Colorbar at 0x7f5539c2d1f0>
```

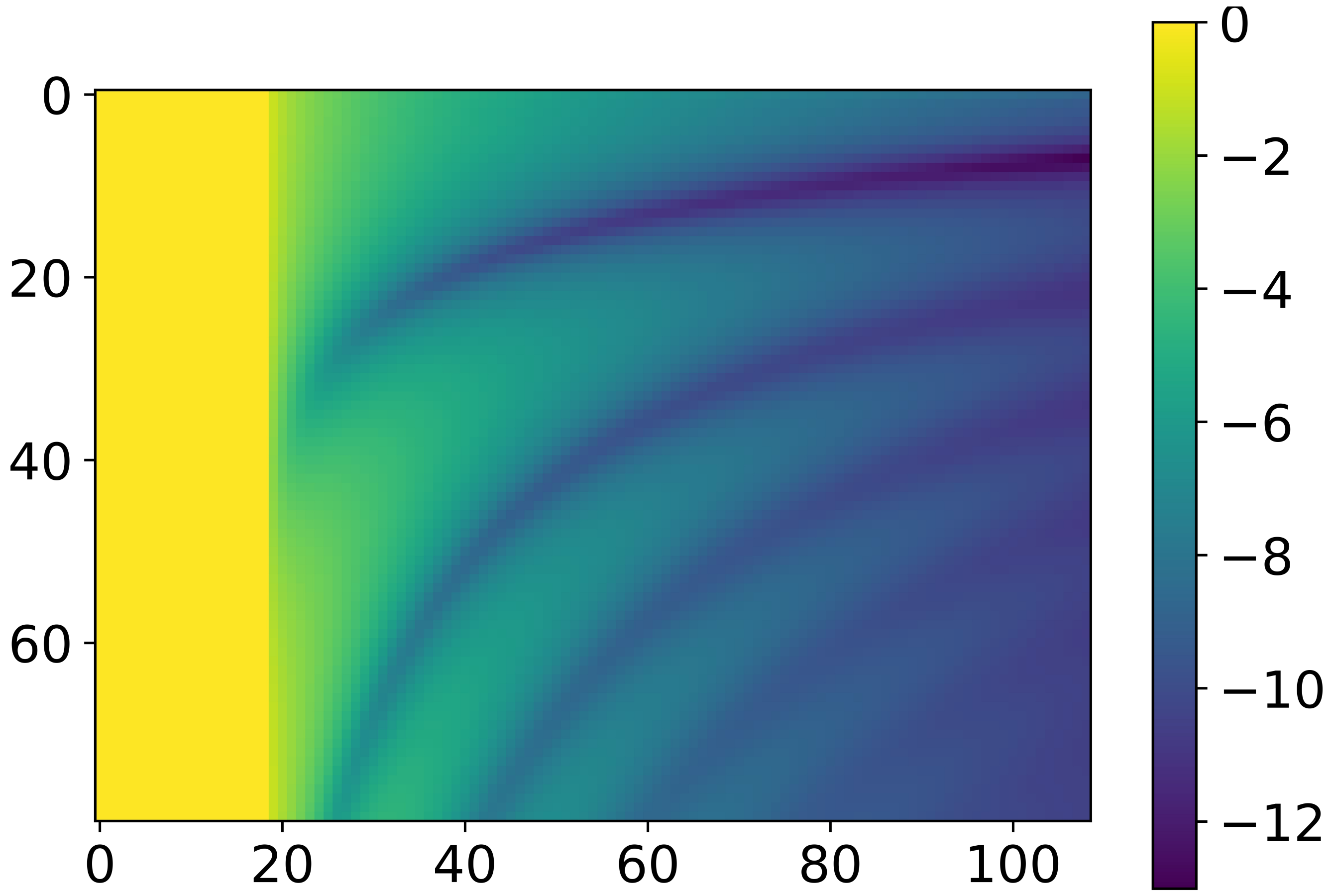

In [ ]:

In [ ]:

In [ ]:

Loading [MathJax]/jax/output/CommonHTML/fonts/TeX/fontdata.js
